# Drug Loading and Release: Development and Characterization of a Novel Therapeutic Agent-Nanographene Conjugate

**DOI:** 10.1021/acs.jpcb.5c04238

**Published:** 2025-09-01

**Authors:** Kaiyue Hu, Agnese Pavan, Alessandro Semeraro, Alberto Ongaro, Luigi Brambilla, Maria Cristina De Rosa, Matteo Tommasini, Chiara Castiglioni, Michele Maggini

**Affiliations:** † Dipartimento di Chimica, Materiali e Ingegneria Chimica Giulio Natta, 18981 Politecnico di Milano, Piazza Leonardo da Vinci 32, 20133 Milano, Italy; ‡ Dipartimento di Scienze Chimiche, Università di Padova, Via F. Marzolo 1, 35131 Padova, Italy; § Dipartimento di Chimica e Tecnologie del Farmaco, Sapienza, Università di Roma, P.le A. Moro 5, 00185 Roma, Italy; ∥ Istituto di Scienze e Tecnologie Chimiche Giulio Natta (SCITEC)-CNR, Roma, L.go F. Vito 1, 00168 Roma, Italy; ⊥ Istituto di Chimica della Materia Condensata e di Tecnologie per l’Energia (ICMATE)-CNR, Padova, Corso Stati Uniti 4, 35127 Padova, Italy

## Abstract

Compound **8**, (5*Z*)-2-hydroxy-4-methyl-6-oxo-5-[(5-phenylfuran-2-yl)­methylidene]-5,6-dihydropyridine-3-carbonitrile, is an effective NEK6 kinase inhibitor with demonstrated anticancer and neuroprotective activity. However, its poor aqueous solubility (3 μg/mL) presents a significant barrier to therapeutic development. To address this limitation, we developed a graphene-based nanocarrier system by conjugating compound **8** and its fluorinated derivatives (**8-F** and **8-CF**_**3**_) onto structurally uniform few-layer graphene nanoparticles (GNPs) obtained via ball-milling and liquid-phase exfoliation (B60). The resulting conjugates (**8**@B60, **8-F**@B60, and **8-CF**_**3**_@B60) were thoroughly characterized by UV–vis, IR, and Raman spectroscopy, as well as by TEM and STEM–EDX analysis. Spectroscopic and elemental data confirmed effective drug loading and structural preservation of the B60 nanocarriers. Drug release experiments further confirmed thermally triggered desorption of **8** from the GNPs surface in aqueous conditions, highlighting the potential of B60-based conjugates as controlled release systems. A key finding of this work is the reversible hydration of compound **8** in aqueous solution, resulting in a colorless, nonconjugated species. Detailed spectroscopic and computational studies revealed that this hydrated form likely represents the dominant species under physiological conditions. Crucially, molecular docking and molecular dynamics simulations demonstrated that hydration does not compromise the binding affinity of compound **8** for the NEK6 active site. These insights provide a molecular-level rationale for the design and evaluation of drug delivery systems based on nanographene platforms in aqueous environments.

## Graphical abstract

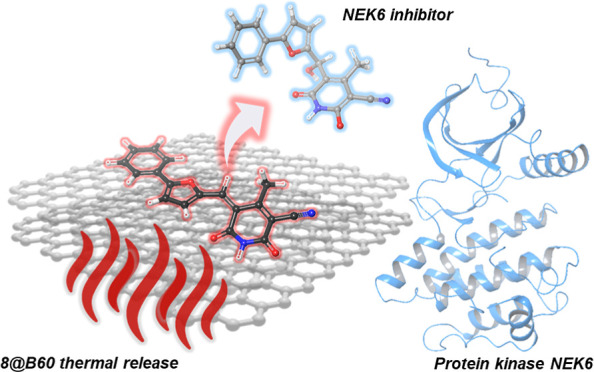

## 1 Introduction

NEK6 (Never In Mitosis A (NIMA) related kinase 6) is a mitotic kinase activated by NEK9 and stress, essential for spindle assembly and chromosomal stability.^1,2^ In cancer, it promotes antioxidant defense, DNA repair, and cell survival through NF-κB2 and ATM/γH2AX pathways.^3^ Overexpressed in cancers like castration-resistant prostate cancer (CRPC) and hepatocellular carcinoma (HCC),^3,4^ NEK6 upregulations are associated with decreased sensitivity to cisplatin in the human ovarian carcinoma cell line A2780.^5^ Targeting NEK6 with the ATP-competitive inhibitor **8** ((5*Z*)-2-hydroxy-4-methyl-6-oxo-5-[(5-phenylfuran-2-yl)­methylidene]-5,6-dihydropyridine-3-carbonitrile, Chart 1) has demonstrated antiproliferative activity across various human cancer cell lines and showed synergistic effects with chemotherapy.^6^ Highlighting the therapeutic potential of compound **8**, recent studies have shown that NEK6 inactivation by **8** alleviates cardiac dysfunction^7^ and may enhance neuronal survival in the late stages of amyotrophic lateral sclerosis (ALS) progression.^8^

**Chart 1:**
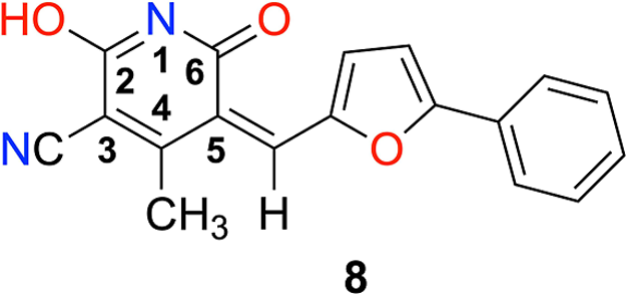
Molecular Structure of NEK6 Inhibitor **8**; The Number is Taken from Reference 6

Despite its therapeutic potential, compound **8** suffers from low aqueous solubility (3 μg/mL), which may limit its development as a viable drug candidate. Improving solubility without affecting biological activity remains a significant challenge in drug development. To address this, several strategies have been explored, including structural modification of the candidate molecule, the application of next-generation drug delivery nanosystems or nanomatrices, or a combination of both approaches.^9,10^ In this context, we propose here the immobilization of compound **8** onto the surface of graphene nanoparticles (GNPs) as a viable strategy to overcome the solubility issue while preserving its biological efficacy. Among carbon-based nanosystems,^11^ GNPs are particularly appealing^12–22^ due to their ease of preparation, small size, low toxicity, high drug-loading capacity, and favorable release profiles.^23–25^ Their surfaces feature a variety of functional groups that (i) enable control over properties such as aqueous solubility, and (ii) facilitate conjugation with drug molecules or targeting structures, such as peptides, through covalent bonds, electrostatic interactions, hydrogen bonding, or π–π stacking.^25–32^ Studies on the cytotoxicity, biocompatibility and biodegradation of GNPs strengthen their applications as drug nanocarriers.^33–40^

GNPs can be produced according to a variety of protocols, usually classified as either chemical or physical methods. The chemical route involves synthesizing graphite oxide from graphite, followed by exfoliation to obtain graphene oxide (GO).^41,42^ Subsequent chemical reduction yields reduced GO (r-GO), a material that retains domains with the hexagonal structure characteristic of graphene. Most studies on graphene-based drug delivery systems rely on GO or r-GO nanoparticles^20–24,43^ and various commercial grades of these GNPs are available. However, the inability to precisely control their structural features hampers accurate performance assessment, particularly in biomedical applications. Key parameters, such as the average lateral size of the graphene sheets ⟨*L*⟩, the GNPs thickness (average number of graphene layers, ⟨*N*⟩), the nature and extent of chemical or structural defects, and the particle size distribution, significantly influence how GNPs interact with biological tissues and cells. These factors determine their cellular uptake and cytotoxicity.^33–40^ Without accurate structural characterization, establishing reliable structure–performance relationships remains challenging. GNPs produced by physical methods, such as ball milling of pure graphite followed by liquid-phase exfoliation, offer a promising alternative to *r*-GO. This approach enables good control over defect density and yields structurally homogeneous GNPs with small size and thickness. We recently developed a protocol^44^ that produces highly uniform GNPs (B60) with ⟨*L*⟩ ≈ 120 nm and ⟨*N*⟩ ≈ 6 layers.^25^ Carboxylic acid groups formed during processing decorate the sheet edges, providing anchoring sites for functional molecules. Spectroscopic analyses confirmed the high structural integrity and reproducibility of B60.^25,40,44^ These GNPs can effectively load π-conjugated small molecules via π–π interactions, enabling thermally triggered release and supporting their potential in controlled drug delivery.^25^

In this work, we demonstrate for the first time that B60 GNPs are effective nanoplatforms for drug loading. Specifically, we report the preparation and comprehensive characterization of conjugates between compound **8** and B60 (**8**@B60), through π–π stacking. UV–vis, IR, and Raman spectroscopies confirm the successful conjugation of the drug to the nanoparticle surface, while transmission electron microscopy (TEM) analysis shows that the morphology of B60 remains unaltered upon loading. The resulting **8**@B60 conjugates are stable in aqueous dispersion, highlighting their potential for drug delivery applications. Moreover, we show that compound **8** can be released in water under mild heating conditions, supporting the feasibility of a thermally controlled release system. To understand the spectroscopic features of the **8**@B60 conjugate, a thorough analysis of compound **8** was conducted in both solution and solid state. This investigation provided a comprehensive overview of its chemical and physical properties and enabled interpretation of the spectra of the conjugates. Reference spectra of free **8** and of B60 were used as benchmarks to rationalize the optical and vibrational profiles of **8**@B60, offering insight into the structural form adopted by the drug upon immobilization. Density functional theory (DFT) calculations supported the experimental results, showing that compound **8** preferentially adopts the (*Z*)-2,6-dipyridone form (vide infra) in the solid state and when loaded onto GNPs. Interestingly, UV–vis spectroscopy revealed a previously unreported phenomenon: the characteristic HOMO–LUMO transition of compound **8**, which accounts for its bright orange color in DMSO and CH_3_OH solutions, progressively diminishes over time, leading to complete discoloration. A series of targeted experiments suggests that this behavior arises from a reversible reaction between **8** and water, yielding a colorless hydrated species. The potential implications of this equilibrium for the pharmacological behavior of compound **8**, particularly under aqueous conditions relevant to drug delivery, were therefore taken into consideration.

Independently, to support the spectroscopic investigation of molecular immobilization onto GNPs, we also synthesized the fluorinated derivatives **8-F** and **8-CF**_**3**_ (Chart 2) and prepared the corresponding conjugates, **8-F**@B60 and **8-CF**_**3**_@B60. These samples were analyzed by energy dispersive X-ray spectroscopy (EDX) coupled with TEM to detect and quantify fluorine content. The observed increase in EDX fluorine signal correlates with the degree of fluorination, providing indirect but complementary evidence, alongside optical spectroscopic data, of successful immobilization on the GNPs surface.

**Chart 2:**
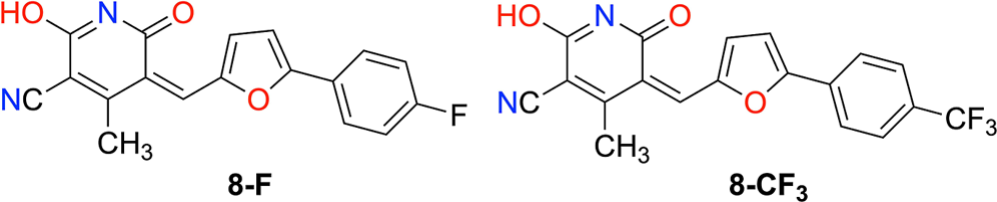
Molecular Structure of the Fluorinated Derivatives of Inhibitor **8**

## 2 Materials and Methods

### 2.1 Synthesis

Chemicals were purchased from Merck and used without further purification. Compound **1** and 5-phenylfuran-2-carbaldehyde are commercially available reagents. 5-(4-Fluorophenyl)­furan-2-carbaldehyde^5,45^ -(4-(trifluoromethyl)­phenyl)­furan-2-carbaldehyde^46^ were prepared as described in the literature. Solvents were analytical grade products. NMR spectra were recorded on a Varian 400 MHz NMR (^1^H: 400 MHz, ^13^C: 101 MHz). DMSO-*d*_6_ was purchased from Sigma-Aldrich and used as received. Chemical shifts are given in ppm at room temperature and are referenced to residual protic impurities in the solvent (^1^H: DMSO: 2.50 ppm), or to deuterated solvent itself (^13^C­{^1^H}: DMSO-*d*_6_: 39.52 ppm). The resonance multiplicities are indicated as “s” (singlet), “d” (doublet), “m” (multiplet). High resolution mass spectra (HRMS) were performed in positive mode (ESI) on an Agilent 6550 IFunnel Q-TOF MS system, via fast-flow-injection technique using a methanolic or aqueous eluent system. The purity of final compounds was assessed via UPLC analysis on an Agilent 1290 Infinity system equipped with a DAD detector (λ = 190–600 nm) under the following conditions: column ZORBAX Eclipse XDB-C18 (2.1 × 50 mm, 1.8 m) at 25 °C, mobile phase A water + 0.1% TFA, mobile phase B CH_3_CN + 0.1% TFA, gradient starting from 5% B and reaching 100% B in 10 min, detection λ = 220 and 254 nm, injection volume 5 ml, compound solution 0.1–0.5 mM in DMSO or water/CH_3_CN. UV–vis spectroscopy was carried out on a Varian Cary 50 Bio spectrophotometer in a quartz cuvette (edge length = 1 cm) thermostated at 25 °C. For the UV–vis characterization of compound **8** at different pH, aqueous buffers (50 mM) at various pH values were prepared by dissolving the appropriate buffering agents in deionized water: sodium acetate for pH 4 and 5, Na_2_HPO_3_ for pH 7, and Tris-HCl for pH 8 and 9. The pH of each solution was adjusted as needed using 1 M NaOH or HCl. A defined volume (3 μl) of a stock solution of compound **8** in DMSO (10 μM) was added to 1 ml of the selected buffer in a quartz cuvette, and the UV–vis spectrum was recorded. The morphology and microstructure of the **8**@B60, **8-F**@B60, and **8-CF**_**3**_@B60 conjugates were investigated by transmission electron microscopy (TEM) and high-angle annular dark-field scanning transmission electron microscopy (HAADF-STEM), using a JEOL F200 microscope operated at 200 kV. Elemental analysis and mapping were carried out with a 100 mm^2^ silicon drift detector (SDD) energy-dispersive X-ray spectrometer (EDX) from JEOL. Samples were prepared by depositing an aqueous suspension of the conjugates onto a 400-mesh lacey carbon grid. Full experimental details for the synthesis of compound **8**, **8-F** and **8-CF**_**3**_ are reported in the Supporting Information.

#### 2.1.1 Preparation of B60 GNPs

Graphene nanoparticles were prepared by the method previously reported.^44^ 500 mg of a microcrystalline graphite rod (Sigma-Aldrich 496561-240.5G, 99.995% carbon), was grounded with a Retch Mixer Mill MM 400 for 200 min. 80 mg of the obtained powder was added to 24 ml of sterile water (molecular biology reagent grade water, Sigma-Aldrich: W4502-1L) in a vial for sonication (5 min). The sonication was performed at room temperature using an Ultrasonic Processor GEX750 with a flat head tip, 450 W, at 20 kHz. A fraction of small-sized GNPs was selected by a first centrifugation procedure (5000 rpm for 10 min). By a second centrifugation cycle (5000 rpm for 60 min), GNPs were separated in two fractions: the supernatant named T60, and the sediment named B60. B60 were used for subsequent loading of compound **8** and its fluorinated derivatives **8-F** and **8-CF**_**3**_.

#### 2.1.2 Loading of B60 General Procedure

**8**@B60 was prepared by mixing B60 (6.2 mg) and compound **8** (1.24 mg) in CH_3_OH (4.3 ml) and sonicating the mixture for 45 min in an ultrasonic bath. Subsequently, 12.24 ml of sterile water was added to the dispersion, and sonication was continued for 24 h. The resulting aqueous dispersions (**8**@B60_0W) was subjected to several washing cycles to remove excess unbound compound **8** and methanol. The washing procedure, described in detail in ref 25, was carried out repeatedly (M cycles, M=1-4), yielding the final dispersions referred to as **8**@B60_MW. The conjugates **8-F**@B60 and **8-CF**_**3**_@B60 were obtained using the same protocol.

#### 2.1.3 UPLC-HRMS Determination of Compound **8** Loaded on B60

An aqueous suspension (1 ml) of **8**@B60 containing a concentration of 9.45 μg/ml of B60 was heated at 70 °C for 1 h in a 2 ml glass-stoppered vial. The suspension was then centrifuged at 15,000 rpm, and the resulting supernatant was analyzed by UPLC coupled to a HRMS instrument (see above for the description). The chromatographic conditions were as follows: ZORBAX Eclipse XDB-C18 column (2.1 × 50 mm, 1.8 μm), maintained at 25 °C; mobile phase A: water + 0.1% TFA; mobile phase B: CH_3_CN + 0.1% TFA; linear gradient from 5% to 100% B over 10 min; detection wavelength: 480 nm; injection volume: 3 μl. The concentration of compound **8** in the supernatant was determined using a previously established calibration curve.

### 2.2 Spectroscopic Analyses of the Conjugates

Water dispersions of **8**@B60, **8-F**@B60, **8-CF**_**3**_@B60 conjugates were analyzed by UV–vis absorption spectroscopy (JASCO V-570 spectrophotometer, Japan) in quartz cuvettes (volume of 1 ml, optical path of 1 cm). IR spectra of the conjugates and of bare B60 were recorded in specular reflection (SR) mode with a Thermo Nicolet 6700 FT-IR spectrometer, coupled to a ThermoElectron-Nicolet Continuμm FT-IR microscope (15× Infinity Corrected Cassegrain objective, 512 scan, and 4 cm^–1^ resolution). The SR measurements were performed on a flat and uniform layer of dried sample. It is mandatory to prepare films with a thickness higher than few hundreds microns to collect only the specular reflected IR signals. To convert the SR spectrum in an absorption spectrum, we applied the Kramer–Kronig (KK) transformation^47^ by means of OMNIC 8.0 software [see 269-032217-Ver 8.0-OMNIC User Guide.pdf]. IR spectra of solid **8**, **8-F** and **8-CF**_**3**_ were recorded with the same instrument using a diamond anvil cell (DAC) accessory; CHCl_3_ solutions were analyzed in transmission mode, using an IR liquid cell with a KBr window. Raman Spectroscopy of conjugates were carried out with a Jobin Yvon LabRam HR800 Raman spectrometer, coupled to an Olympus BX41 microscope, with a 50× objective and a He–Ne laser at 633 nm for the excitation. The powdered conjugates were deposited on an aluminum foil fixed on a glass slide (scan time 120 s with a spectral resolution of 2 cm^–1^). Raman spectra of pure **8**, **8-F** and **8-CF**_**3**_ were recorded with a Nicolet NXR 6500 FT-Raman spectrometer (excitation laser at 1064 nm and 2048 scans). In each experiment, the power of the laser was carefully set to prevent degradation or photoinduced changes of the samples during the acquisition of the spectrum.

### 2.3 Molecular Modeling

The early conformational analysis of compounds (*Z*)-2,6-dipyridone, (*Z*)-6-pyridone, (*Z*)-2-pyridone was carried out with the CREST code using default settings and the GFN2-xTB Hamiltonian.^48^ The subsequent calculations aimed at simulating the vibrational spectra were carried out with the Gaussian code^49^ using the B3LYP/6-311++G­(d,p) DFT method including empirical D3BJ dispersion. We fully optimized the molecular structures indicated in the main text by DFT, using the lowest energy conformations determined by CREST as a starting point. The IR and Raman spectra were simulated using a linear combination of Lorentzian functions centered at the computed wavenumber and possessing integrated areas proportional to the intensities calculated by DFT. The fwhm was arbitrarily set to 10 cm^–1^. When indicated, the positions of the wavenumbers calculated by DFT were scaled by the factor 0.98, as customary when comparing experimental vibrational spectra with DFT simulations.^50^ The simulation of the UV–vis spectra was carried out considering the same minima obtained for simulating the vibrational spectra and the same functional, adopting the time-dependent DFT method (TDDFT) as implemented in the Gaussian code.^49^ The spectra were simulated from the TDDFT outputs (100 computed states) using the polar code [https://github.com/matteo-maria-tommasini/polar] and the theoretical approach described in ref 51.

We computed the Fukui function^52^ of (*Z*)-6-pyridone suitable for interpreting nucleophilic attacks, namely *f*_+_(**r**) = *n*_anion_(**r**) – *n*_neutral_(**r**), directly from its definition as the difference between the respective cube files of the electron densities computed by Gaussian, considering the equilibrium structure of the neutral form.

Molecular docking calculations were performed using AutoDock version 4.2.6,^53^ following the protocol described by De Donato et al.^6^ The docking grid was centered on the binding site of compound **8** and defined with dimensions of 60 × 60 × 60 grid points and a spacing of 0.375 Å. The Lamarckian genetic algorithm was employed for the docking process. Default parameters were applied, except for the “number of GA runs,” “population size,” and “maximum number of evaluations,” which were set to 10, 50, and 2.5 × 10^5^, respectively. Molecular dynamics (MD) simulations were conducted to evaluate the conformational stability and receptor flexibility of the top-ranked docked complexes of NEK6 using the Desmond module in the Schrödinger 2024-3 software suite, as implemented in Maestro. Each system was simulated in triplicate, with each run lasting 500 ns. Each system was solvated in an orthorhombic SPC/E water box with a 10 Å buffer and neutralized with 0.15 M NaCl. Long-range electrostatics were computed using the particle-mesh Ewald method, and a 9.0 Å cutoff was applied for short-range interactions. Systems were energy-minimized and equilibrated by restrained *NVT* and *NPT* simulations. Production MD runs were then performed in the *NPT* ensemble using the OPLS4 force field, maintaining a temperature of 300 K and pressure of 1 atm via the Nosé–Hoover thermostat and Martyna–Tobias–Klein barostat.^54^ Rmsd analyses were performed using Maestro’s Simulation Interaction Diagram tool.

## 3 Results and Discussion

### 3.1 Compound **8** and Its Fluorinated Derivatives: Synthesis, Structure, and DFT Theoretical Modeling

#### 3.1.1 Synthesis and Structure

The synthesis of compound **8** and of the fluorinated derivatives **8-F** and **8-CF**_**3**_ can be illustrated through the retrosynthetic analysis shown in Scheme 1 which involves a Suzuki coupling reaction between 2-formylfuran-5-boronic acid and the corresponding aromatic bromide, followed by a Knoevenagel reaction between the product of the Suzuki reaction and compound **1**.

**Scheme 1:**
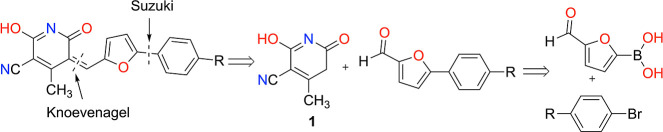
Retrosynthetic Analysis for the Preparation of Inhibitor **8** and Its Derivatives **8-F** and **8-CF**_**3**_

Compound **8** was synthesized by condensation of 5-phenyl-2-furaldehyde with 2-pyridone **1** in the presence of β-alanine in acetic acid (Scheme 2).^55^ Derivatives **8-F** and **8-CF**_**3**_ were prepared analogously from the corresponding substituted furfurals^45,46^ in high yields (≥90%) and >95% purity (UPLC). The three products were characterized by ^1^H NMR, ^13^C NMR, optical spectroscopies, and high-resolution mass spectrometry (Figure S1–S6).

**Scheme 2:**
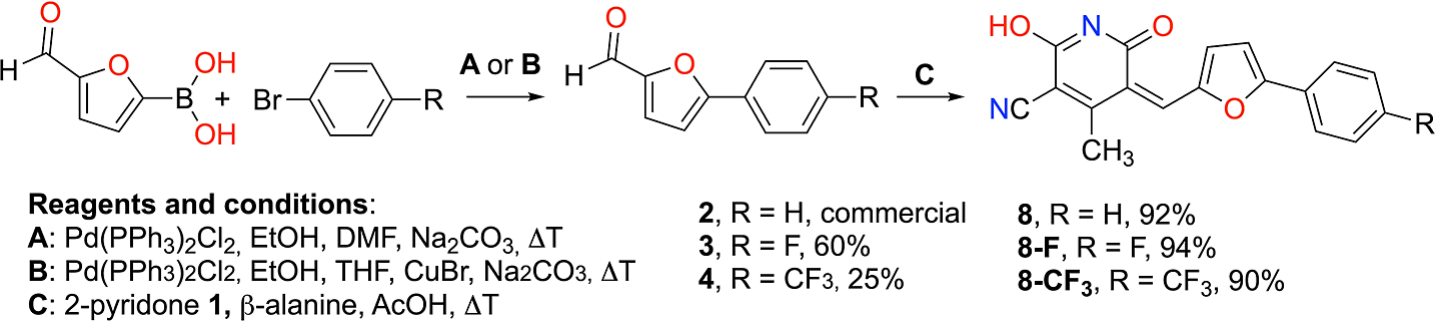
Synthesis of Inhibitor **8** and Its Derivatives **8-F** and **8-CF**_**3**_

Compound **8**, **8-F** and **8-CF**_**3**_ can exist as two diastereoisomers, *E* and *Z*. Each of these can, in turn, interconvert between three possible tautomeric forms, as shown in Scheme 3. Each tautomer can assume several stable conformations characterized by distinct values of the torsional angles around the two inter-ring C–C bonds. Further torsional degrees of freedom determine the –OH and –CH_3_ orientation. Effective π-conjugation across the entire molecule favors planar structures, suggesting that *Z* diastereoisomers are preferred over *E* diastereoisomers, which instead undergo significant distortion from planarity due to steric hindrance between the methyl group and the furan ring. This hypothesis was confirmed by a two-dimensional NOESY NMR experiment performed on compound **8**, shown in Figure S7. The observed correlation between the signals of the vinyl proton and the methyl protons on the heterocyclic ring reveals a clear NOE effect, indicating that these nuclei are spatially close.

**Scheme 3:**
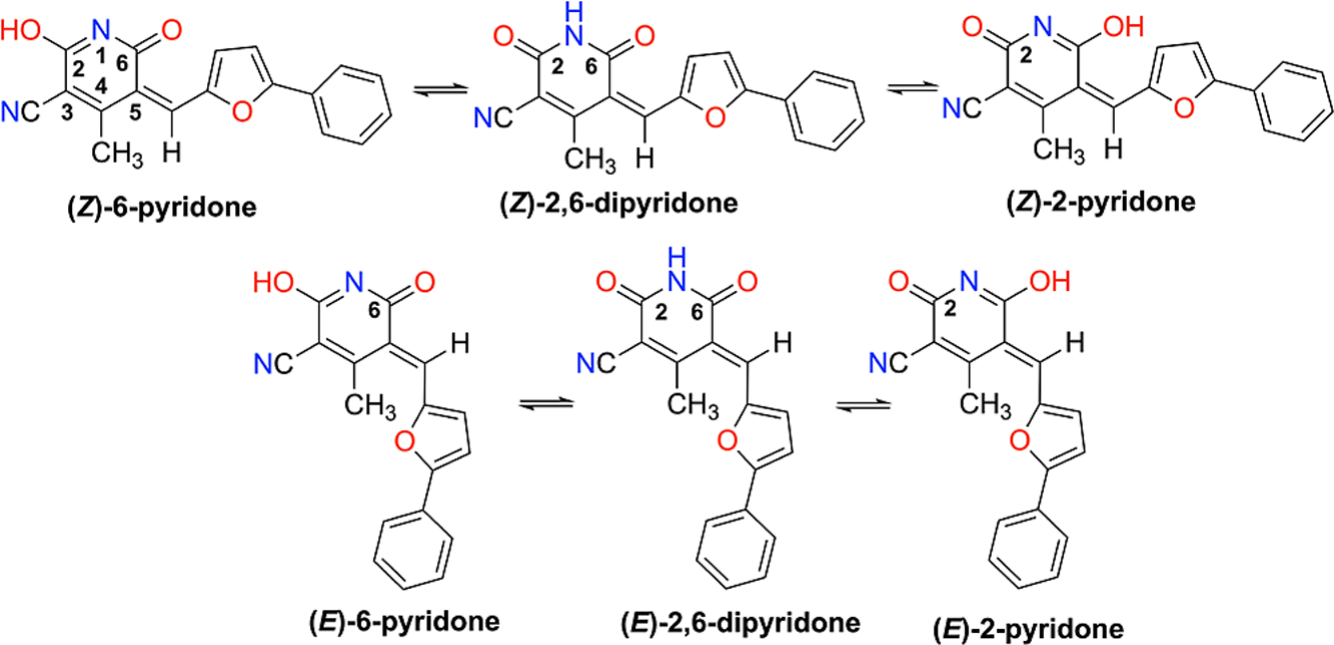
Diastereomeric (*E*/*Z*) Pairs of Compound **8** in the Three Tautomeric Forms Hydroxypyridine-Pyridone

#### 3.1.2 DFT Modeling: Conformations, Ground State Energies and Spectroscopic Response

The existence of multiple tautomers and molecular conformations can reasonably be assumed also for derivatives **8-F** and **8-CF**_**3**_, as substitution on the phenyl ring has only a minor influence on the heterocyclic ring and the torsional angle between the rings. Consequently, theoretical modeling is limited to the parent compound **8**. Since the (*Z*)-tautomers are expected to be the most stable, we carried out a detailed conformational study on those structures, aimed at screening their equilibrium conformations. A quick tight binding approach (GFN2-xTB method) enabled the selection of the five lowest energy geometries for compound **8**, which were subsequently optimized via B3LYP/6-311++G­(d,p) DFT calculations, including empirical D3BJ dispersion—see Materials and Methods for details.

Figure 1 presents the five optimized (*Z*)-structures of compound **8**, all exhibiting planar or nearly planar equilibrium geometries that enable extended π-conjugation across the molecular framework. In particular, the (*Z*)-6-pyridone and (*Z*)-2,6-dipyridone tautomers are further stabilized by intramolecular interactions between the carbonyl oxygen and a proximal hydrogen atom on the furan ring. Interestingly, the (*Z*)-2,6-dipyridone tautomer can also form dimers, stabilized by strong intermolecular NH···O=C hydrogen bonds. The two optimized dimeric structures, also shown in Figure 1, are especially relevant for interpreting the vibrational response observed in solid-state spectroscopic experiments (vide infra). Both dimers feature pairs of quasi-linear hydrogen bonds, leading to notable stabilization energies: Δ*E* ((*Z*)-2,6-dipyridone *d*_1_) = 13.8 kcal/mol and Δ*E* ((*Z*)-2,6-dipyridone *d*_2_) = 16.7 kcal/mol.

**Figure 1:**
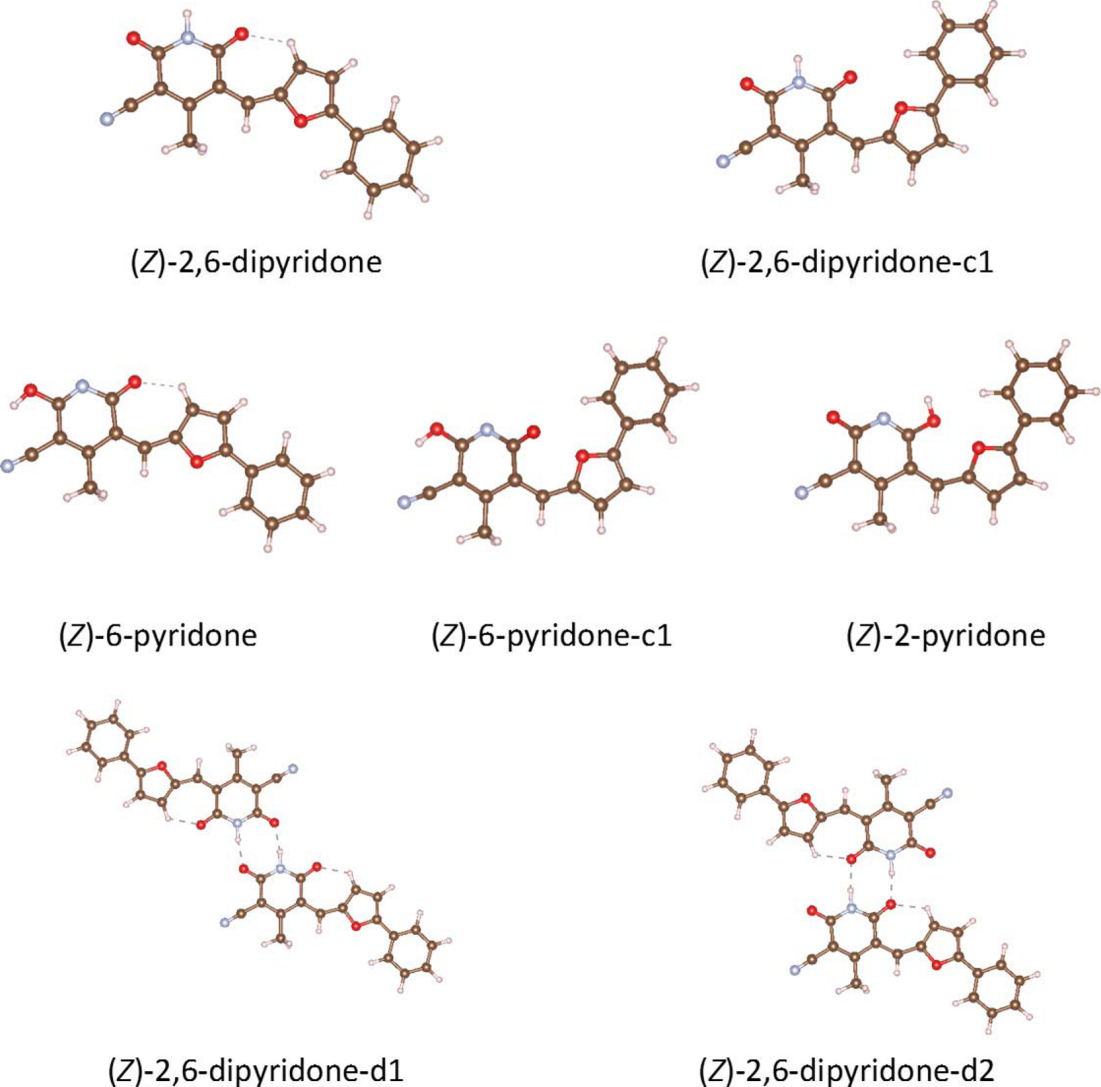
DFT-optimized structures of the low-energy (*Z*)-tautomers and selected conformers of compound **8** and of two hydrogen-bonded dimers of (*Z*)-2,6-dipyridone. DFT calculations have been performed on the isolated molecules (or dimers) at the B3LYP/6-311++(d,p) level, including D3BJ empirical dispersion.

Table 1 summarizes the ground state relative energies of the five optimized (*Z*)-structures reported in Figure 1 and of two conformers of (*E*)-2,6-dipyridone (Figure S8) that were considered to check the effects of deviation from planarity. As expected, the resulting reduced π-conjugation makes these two conformers less stable than those of the (*Z*)-2,6-dipyridone counterpart.

**Table 1:** Relative Ground State Energies of Five (*Z*)-Structures and of Two (*E*)-Tautomers of Compound **8** from DFT Calculations (See Scheme 3 and Figure 1 for the Names)^a^

	(*Z*)-2,6-dipyridone	(*Z*)-2,6-dipyridone-c1	(*Z*)-6-pyridone	(*Z*)-6-pyridone-c1	(*Z*)-2-pyridone	(*E*)-2,6-dipyridone-c1	(*E*)-2,6-dipyridone
Δ*E* (kcal/mol)	0.0	4.3	20.0	24.2	22.2	4.0	5.7
λ (nm)	434	441	453	465–481	448	451	453

a The wavelength (λ) corresponding to the lowest energy electronic transition obtained from TDDFT calculations is also reported. DFT and TDDFT calculations have been carried out in the gas phase at the B3LYP/6-311++G­(d,p) level, including D3BJ empirical dispersion.

For the structures of compound **8** reported in Figure 1, we calculated the spectroscopic observables relevant for interpreting the experimental data. The UV–vis absorption spectra simulated by TDDFT of the isolated molecules (Figure S9) and the computed wavelength of the lowest energy electronic transition (Table 1) support in all cases a strong HOMO–LUMO character.

As reported in Table 1, the HOMO → LUMO transition occurs in the 430–480 nm range for the entire set of different structures of compound **8**. The position of the predicted absorption features accurately accounts for the intense orange/red color of solutions and crystals of **8**.

The IR and Raman spectra predicted by DFT for the five lowest energy (*Z*)-structures of **8** are reported in Figures S10 and S11. A comparison of the theoretical spectra (see discussion in Supporting Information) indicates that there are distinctive features, particularly in the IR, which help to identify the molecular structure by the presence or absence of several vibrational transitions. The relevant marker bands of the different tautomers of compound **8** will be illustrated in detail in Section 3.2, showing the comparison with the experimental vibrational spectra.

### 3.2 Spectroscopic Characterization of Compound **8**

#### 3.2.1 UV–Vis Spectra of Compound **8** in Different Solvents

UV–vis spectroscopy has been successfully employed in the past for characterizing GNPs dispersions in water and, more recently, for detecting the conjugation with a small molecule.^25,44^ The spectra of the conjugates are analyzed by comparing them to the reference spectra of bare GNPs dispersions and the solution of the guest molecule. Thus, this section is dedicated to the UV–vis characterization of compound **8** in different solvents and in the solid state.

The UV–vis spectra of compound **8** in CH_3_OH, CHCl_3_, DMSO, and CH_3_CN, shown in Figure 2, reveal two distinct behaviors. In CH_3_OH and DMSO, two main absorption bands are observed: band I is a structured feature spanning 250–350 nm, with a lower-energy peak centered around 330 nm; band II appears as a broad band with a maximum near 480 nm. The relative intensity of band I vs. band II is larger in methanol than in DMSO. In CHCl_3_ and CH_3_CN, compound **8** exhibits a single prominent absorption band (band II) centered around 480 nm. A similar spectral feature is observed in the solid state, where band II remains the dominant transition but is noticeably red-shifted, with a maximum at 580 nm. Additionally, weak absorption features are detected in the 250–350 nm range for both CHCl_3_ and CH_3_CN solutions.

**Figure 2:**
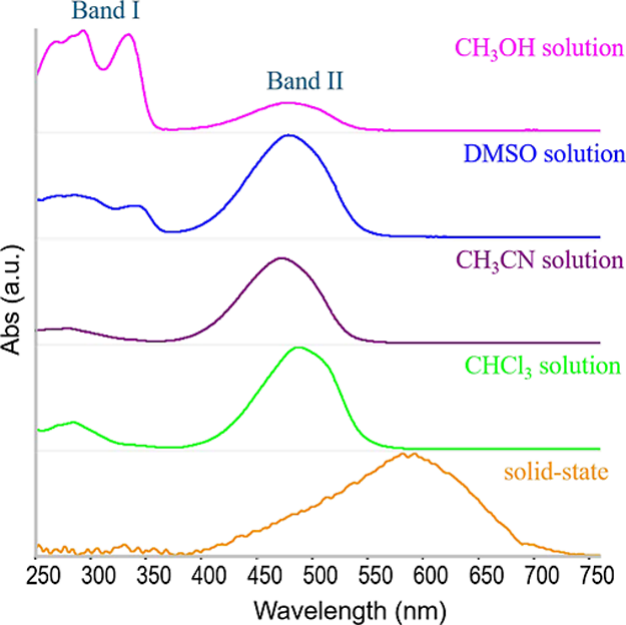
UV–vis absorption spectra of compound **8** in different solvents and in the solid state.

According to TDDFT calculations, band II corresponds to the HOMO → LUMO transition of compound **8**. Since the transition lies in the blue-green region of the visible spectrum, it accounts for the orange color observed in solutions of **8** and for the reddish hue of the crystalline powder. The UV–vis spectrum does not allow discrimination among the various tautomers or conformers of **8**, as the calculated λ_max_ values of band II are too close in energy. Moreover, solvent effects may shift the band position more significantly than the subtle differences predicted by DFT calculations for the individual structures of compound **8**.

The assignment of band I is more puzzling, since no strong transitions are predicted by TDDFT for any tautomers or conformers of **8** in this energy range. Interestingly, an increase of the intensity of band I at the expense of band II is observed in CH_3_OH and DMSO solutions over time, and by heating above ambient temperature, which suggest that the intensity modulation of the two bands results from the equilibrium between different species.

An initial hypothesis attributed band I to highly distorted conformations of compound **8**; however, TDDFT calculations did not support this interpretation, as conformational changes produced only a minimal blue-shift in the HOMO–LUMO transition. In contrast, experiments performed in CH_3_OH/H_2_O and DMSO/H_2_O mixtures suggested an alternative explanation, prompting consideration of a possible reaction between **8** and water. When 3 μl of a solution 6.9 mM of **8** in DMSO were added to 1 ml of CH_3_OH, the initial orange color faded within minutes without any visible precipitation of **8**. As shown in Figure 3, the initial UV–vis spectrum shows both band I and band II. After 12 min, band II has practically disappeared, and only band I remained, in the 250–350 nm range. The same behavior is observed also for compounds **8-F** and **8-CF**_**3**_. Figure S12 shows the respective UV–vis spectra in CH_3_OH recorded immediately after preparation and at increasing time intervals. This reaction was reversible: upon addition of a few μl of acetic acid, the color reappeared immediately, and the UV–vis spectrum once again displayed a strong band II centered at 480 nm. Simultaneously, band I weakened significantly in the acidic medium (Figure 3). Interestingly, if compound **8** is dissolved in dry methanol under inert atmosphere, it keeps its orange color for days without any noticeable discoloration (Figure S13).

**Figure 3:**
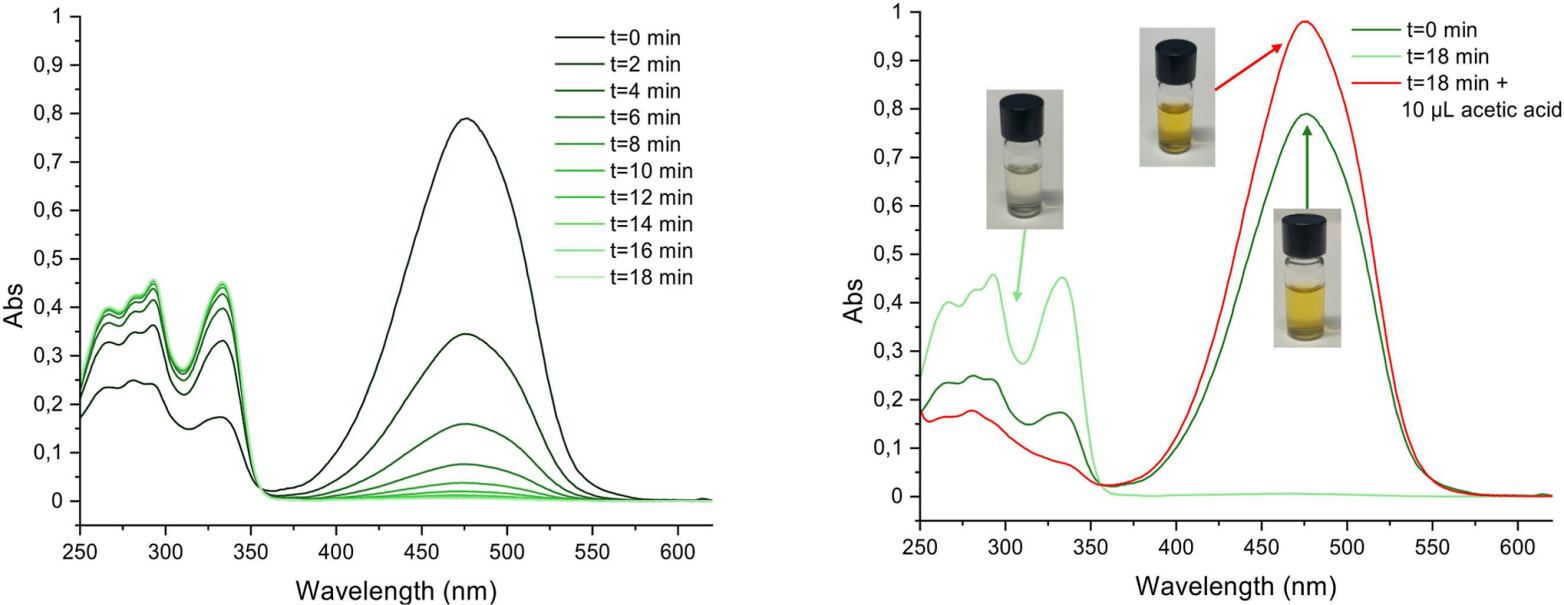
UV–vis spectra of compound **8** in CH_3_OH under different conditions. Left panel: spectra recorded immediately after sample preparation and at increasing time intervals, showing the evolution of the absorption profile over time. Right panel: spectrum of the same solution before and after the addition of acetic acid, highlighting the reversibility of the spectral changes under acidic conditions with photographic representation of the solution color corresponding to each condition.

The above experiments indicate that the discoloration phenomenon is associated with the presence of water and that band I arises from a reversible transformation of compound **8** upon reaction with water present in reagent-grade methanol. Discoloration is also observed in an aqueous solution of compound **8**, which restores its original orange color upon addition of acetic acid (Figure S14). In addition, the process is accelerated by heating. Although molecular aggregation might influence the absorption profile, the persistence of the spectral changes in band II after both sonication and mild heating suggests that aggregation is unlikely to be responsible for the observed effects. The hydration of **8** must somehow disrupt the π-electron delocalization in its structure to account for the loss of color. Moreover, the process has to be both reversible and acid-catalyzed. The mechanism reported in Scheme 4 represents a possible transformation involving a nucleophilic attack of water on the (*Z*)-6-pyridone tautomer, leading to the formation of a nonconjugated hydrated species, which is potentially responsible for the loss of absorption in the visible region.

**Scheme 4:**
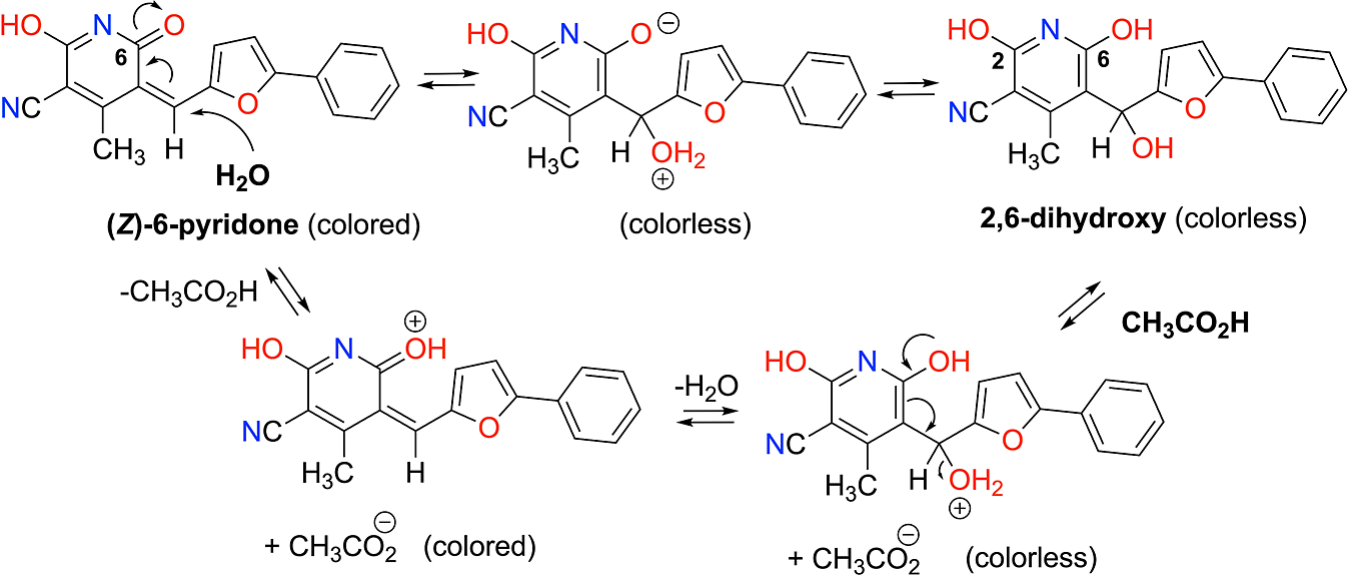
Hydration of Compound **8**, Reversible upon Acid Addition

Indeed, the DFT calculation of the Fukui function *f*_+_(**r**) = *n*_anion_(**r**) – *n*_neutral_(**r**)—see Materials and Methods for details—as the difference between the electron density of the anion of **8** and its neutral form (both evaluated at the optimized structure of the neutral form) provides evidence of a region suitable for nucleophilic attack at the vinyl site, as indicated in Scheme 4 (Figure S15). Furthermore, the C=C bond length calculated for compound **8** (1.38 Å) is noticeably longer than that of ethylene (1.33 Å), which is considered the prototypical double bond. In addition, the double bond in compound **8** is less rigid, as indicated by a lower force constant (7.8 mdyne/Å vs 9.8 mdyne/Å in ethylene). Together, these findings point to a weakened double bond, which is more susceptible to undergoing the reaction proposed in Scheme 4.

Dehydration under acidic conditions restores the colored parent compound. Similar conclusions can be drawn starting from the quinoid tautomer (*Z*)-2-pyridone; however, we focused on (*Z*)-6-pyridone because, according to TDDFT calculations, it adopts a planar conformation and is more stabilized relative to (*Z*)-2-pyridone due to the presence of an intramolecular hydrogen bond (see Figure 1).

Although methanol and water are comparable nucleophiles, only water reacts with compound **8** to form the hydrated adduct. This selectivity likely arises from water’s superior hydrogen-bonding ability, which stabilizes both the charged or polar intermediates involved in the reaction and the final 2,6-dihydroxy hydrated product. In contrast, dry methanol lacks the capacity to promote efficient proton transfer or to stabilize the nonconjugated adduct, thereby preventing the reaction from proceeding.

To further characterize the system, the UV–vis spectrum of compound **8** was recorded as a function of pH (Figure S16). At pH 9, band II (400–550 nm) is absent. As the acidity increases (from pH 9 to pH 4), a progressive increase in the intensity of band II is observed. Simultaneously, a decrease in absorption in the 250–350 nm region (band I) is recorded, in agreement with previous observations.

The formation of the hydrated adduct of compound **8** was confirmed by NMR spectroscopy after stirring an aqueous solution of **8** at pH 9 for 1 h, followed by lyophilization. ^1^H NMR in DMSO-*d*_6_ is reported in Figure S17.

Fast flow injection (FFI) ESI-MS analysis of compound **8** in negative ion mode, using a pH 9 aqueous buffer as both solvent and eluent, revealed the water adduct [M – H + H_2_O]^−^ at *m*/*z* 321.0847 (calcd 321.0881) as the predominant species, accompanied by a strong UV–vis absorption at 330 nm. When methanol was used instead, as both solvent and eluent, the major signal shifted to the methanol adduct [M – H + MeOH]^−^ at *m*/*z* 335.1044 (calcd 335.1037), with the water adduct observed only as a minor component (integrated area: water adduct = 0.716 × 10^8^; MeOH adduct = 6.252 × 10^8^). To further investigate, MS analysis was performed on the isolated hydrated adduct of compound **8**, previously obtained under basic aqueous conditions, using methanol as both solvent and eluent. In this case, the water adduct remained the major species (integrated area = 8.062 × 10^8^) while the methanol adduct signal was less intense (integrated area = 4.455 × 10^8^) than the water adduct, suggesting that it likely forms from partial dehydration during ionization, followed by methanol addition. These results indicate that compound **8** undergoes addition of water or methanol under MS conditions, depending on the elution solvent. Importantly, the persistence of the water adduct during elution with methanol, when the preformed hydrated species is analyzed, strongly supports the NMR evidence for the formation of a stable, structurally defined hydrated species (Figure S18).

The optimized structure and TDDFT-calculated UV–vis spectrum of the hydrated form of compound **8** (i.e., the 2,6-dihydroxy derivative; see Scheme 4) reveal that the sp^3^ hybridization of the methine carbon bridging the pyridine and furan rings markedly disrupts π-electron delocalization across the conjugated framework (Figure S19).

The HOMO → LUMO transition exhibits a calculated blue shift of the lowest energy electronic transition at 300 nm that aligns well with the experimental observation of band I. The UV–vis absorption spectrum simulated by TDDFT of the isolated 2,6-dihydroxy derivative is reported in Figure S20.

The conclusions drawn in this section are particularly relevant for interpreting the drug loading and release behavior of compound **8** and its fluorinated derivatives when conjugated to B60 graphene nanoparticles in aqueous or methanolic dispersions. Understanding the pH- and solvent-dependent behavior of these compounds provides a mechanistic framework for rationalizing the optical spectroscopy profiles of both immobilized and free molecules during uptake and release processes involving B60. In light of these findings, an important question arises regarding the behavior of compound **8** under physiological conditions: does its transformation in aqueous environments affect its interaction with NEK6? While the initial computational investigation of NEK6 binding was performed using the (*Z*)-6-pyridone tautomer, our results indicate that compound **8** undergoes hydration at near-neutral pH (Figure S13). To determine whether this transformation influences its binding properties, molecular docking and molecular dynamics (MD) simulations were carried out on the hydrated species. Binding pose prediction and affinity estimates were complemented by MD simulations to assess the conformational stability of the ligand within the NEK6 active site. For comparison, the same computational protocol was applied also to the other two (*Z*)-tautomers of **8** presented in Scheme 3.

#### 3.2.2 Binding Modes within NEK6 Active Site

To investigate how the hydrated (2,6-dihydroxy) form and the (*Z*)-tautomers of compound **8** interact with NEK6 kinase, each structure was independently docked into the enzyme’s active site. In this step, the substrate was flexible while the enzyme was kept rigid in geometry. To account for protein flexibility and to allow the system to relax, in a second step the low energy docked conformations were subjected to molecular dynamics simulations. Analysis of the docking scores indicates that all the forms examined have comparable binding affinities for NEK6 (Table 2).

**Table 2:** Summary of AutoDock Results for Docking to NEK6

	predicted affinity (kcal/mol)	rmsd (Å)
(*Z*)-6-pyridone	–7.5	
(*Z*)-2-pyridone	–7.4	2.49
(Z)-2,6-dipyridone	–7.4	7.64
2,6-dihydroxy derivative	–7.0	2.37

The root-mean-square deviation (rmsd), which measures the average distance between two sets of atoms, was calculated for the heavy atoms of the ligands by comparing the predicted poses of the observed forms to the coordinates of (*Z*)-6-pyridone. The results show that both the (*Z*)-2-pyridone and the 2,6-dihydroxy form adopt the same binding mode as the (*Z*)-6-pyridone tautomer, with rmsd values below 2.5 Å. In contrast, the (*Z*)-2,6-dipyridone is shifted within the ATP-binding pocket compared to the predicted position for compound **8**, as indicated by its significantly higher rmsd of 7.64 Å. Predicted binding modes are shown in Figure 4, highlighting the ligand’s interactions with critical amino acids in the active site.

**Figure 4:**
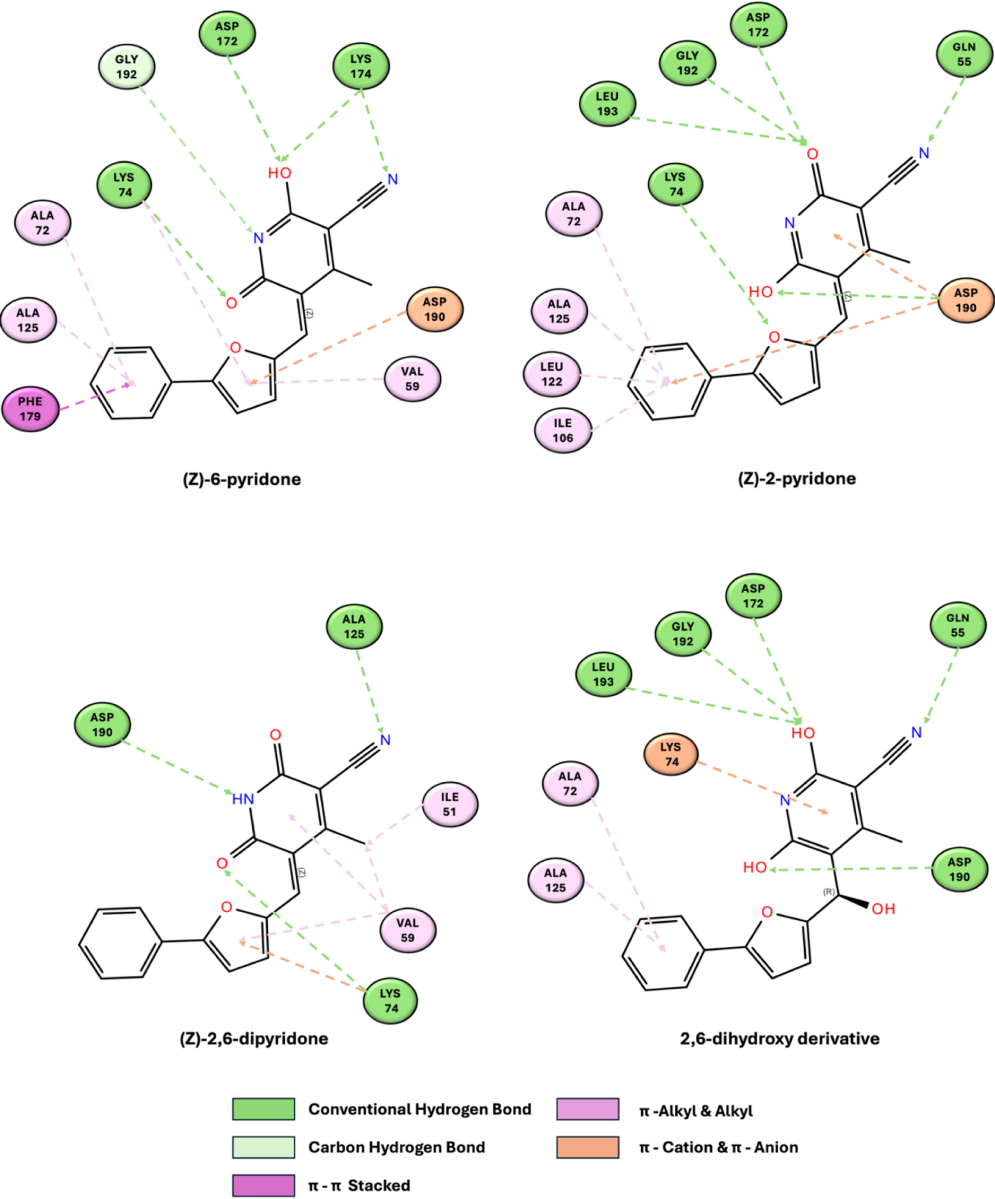
Representation of the docking pose interactions between NEK6 and the four analyzed structures. Dark green indicates hydrogen bonds, light green represents carbon–hydrogen interactions, magenta denotes π–π stacked interactions, pink highlights π–alkyl and alkyl interactions, and orange corresponds to π–cation and π–anion interactions. The figures were generated using BIOVIA Discovery Studio 2025 (BIOVIA, Dassault Systèmes, Discovery Studio, Discovery Studio 2025, San Diego: Dassault Systèmes, 2025).

The (*Z*)-6-pyridone, (*Z*)-2-pyridone, and the 2,6-dihydroxy derivative engage in essentially the same interactions with NEK6, consistent with those previously characterized for the (*Z*)-6-pyridone.^6^ In contrast, the (*Z*)-2,6-dipyridone adopts a distinct binding mode within the ATP-binding pocket, primarily due to a different interaction pattern involving Ala125, Asp190, and Lys74 (Figure 4).

The stability of the predicted complexes was assessed through molecular dynamics (MD) simulations and is reflected by the rmsd fluctuations during time. Scheme S1 illustrates the time evolution of the Cα-rmsd of NEK6, as well as the changes in the ligand rmsd throughout the simulations, providing insight into the dynamic behavior of both the protein and the bound structures. The rmsd values of NEK6 backbone atoms remained consistently stable throughout the 500 ns simulation period, exhibiting a fluctuation in the range of 1.2–4.8 Å (Scheme S1). This behavior indicates that the overall protein structure remained stable in all examined systems. We also analyzed the stability of the ligands bound within the catalytic site. Among all the structures analyzed, only the (*Z*)-6-pyridone tautomer exhibited a stable binding mode, with an average rmsd below 3 Å (specifically, 2.8 Å). In contrast, both the 2,6-dihydroxy and (*Z*)-2,6-dipyridone forms remained bound within the catalytic site but underwent changes in binding orientation during the simulation. The (*Z*)-2-pyridone, by comparison, exhibited even lower stability within the catalytic pocket, sampling multiple orientations throughout the simulation and ultimately dissociating from the binding site (Scheme S1).

#### 3.2.3 Vibrational Spectra of Compound **8**

Figures S21 and S22 compare the IR and Raman experimental spectra of **8** in the solid state (crystalline powder) and in CHCl_3_, the only solvent that provides a suitable spectral window for detecting most IR transitions of **8**. In the fingerprint region (Figure 5), the profiles of the spectra of the crystal and the solution display similar features, with a very good match in the wavenumbers of the main peaks. However, the IR spectra show noticeable differences in the intensity pattern. In CHCl_3_, the NH stretching band appears at 3379 cm^–1^, consistent with the presence of isolated molecules in the (*Z*)-2,6-dipyridone tautomeric form (Figure S21). In the solid state, this band shifts to lower wavenumbers, revealing multiple peaks in the 3200–2800 cm^–1^ range. This shift is attributed to intermolecular hydrogen bonding, and the band multiplicity suggests a complex crystal packing, likely involving several inequivalent molecules of **8** engaged in a hydrogen bond network. Given that the rest of the spectrum closely matches that observed in solution, we conclude that the molecular structure remains essentially unchanged in the crystal and the chloroform solution. The good agreement with the DFT-calculated spectrum for the (*Z*)-2,6-dipyridone form further supports that, in both environments, compound **8** adopts the lowest-energy configuration among those considered.

**Figure 5:**
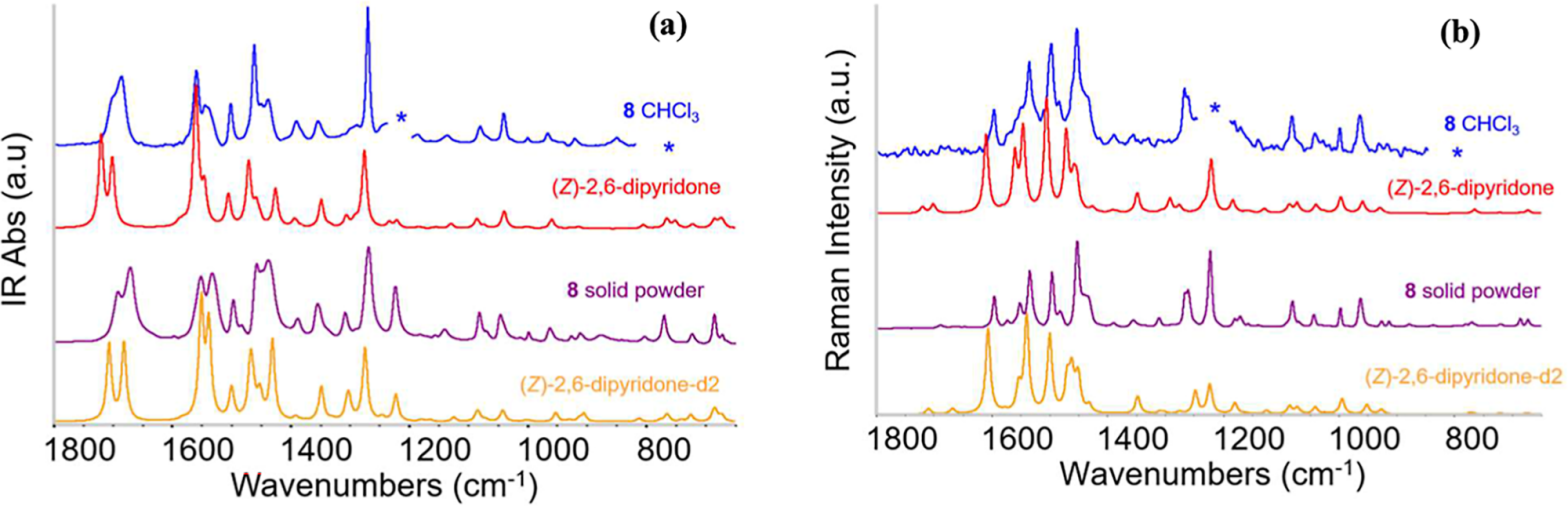
(a) IR spectra of **8** in CHCl_3_ (blue line) and in the solid state (violet line), compared with DFT-calculated spectra (wavenumbers scaled by a factor of 0.98) for the isolated molecule (*Z*)-2,6-dipyridone and for the hydrogen-bonded dimer (*Z*)-2,6-dipyridone-*d*_2_. (b) Raman spectra of **8** in CHCl_3_ (blue line) and in the solid state (violet line), compared with DFT-calculated spectra (wavenumbers scaled by a factor of 0.98), for the isolated molecule (*Z*)-2,6-dipyridone and for the hydrogen-bonded dimer (*Z*)-2,6-dipyridone-*d*_2_. The spectra have been vertically stacked for clarity; the asterisk indicates the position of a band of the solvent, which has been removed.

Figure 5 compares the experimental spectra with those predicted by DFT calculations (after appropriate wavenumber scaling), showing that the changes in intensity pattern observed from solution to solid state can be attributed to strong intermolecular interactions. In particular, the most stable hydrogen-bonded dimer of **8** ((*Z*)-2,6-dipyridone *d*_2_, Figure 1) accounts for these effects. The accuracy of the DFT predictions is detailed in Tables S1 and Table S2.

As shown in Figure S23, we compared the experimental spectra of compound **8** in CHCl_3_ and in the solid state with DFT-simulated spectra of its possible tautomeric structures. The results strongly support that the most stable structure is the (*Z*)-2,6-dipyridone: several diagnostic bands observed experimentally are well reproduced by the computed spectrum of this species. In contrast, the spectral features predicted for the alternative tautomers show limited agreement with the experimental data, lacking key features and overall spectral consistency.

### 3.3 Graphene Conjugates of Compound **8** and Its Fluorinated Derivatives

#### 3.3.1 Preparation and Morphology of the Conjugates

The structural features of the GNPs considered in this work (B60), namely their average size and shape, along with the low density of chemical and structural defects, make them well-suited as a drug delivery platform for **8** and its fluorinated derivatives. B60 forms stable water dispersions, a crucial property for enhancing the transport in biological fluids of compounds with limited solubility in water. Furthermore, conjugation with B60 significantly improves the stability of the drug. Among the various graphene-based nanocarriers available, B60 offers distinct advantages, particularly in terms of enabling detailed characterization of the resulting conjugates, an essential step for elucidating drug transport and release mechanisms. The production protocol for B60 has proven highly reproducible,^44^ supporting the development of several strategies, based primarily on spectroscopic methods, to assess its quality. Through a combination of UV–vis, IR, and Raman spectroscopies, we can confirm whether the key features of B60 are preserved, following chemical or physical modifications, such as covalent functionalization or π–π stacking interactions in conjugate synthesis. In our case, the –COOH/–OH groups on GNPs, that form during the ball milling processing of graphite, promote water dispersibility but do not chemically react with compound **8** at room temperature and pH 7.3 (vide infra), corroborating that π–π stacking interactions are the main driving force for conjugate formation. The UV–vis spectrum of B60 in water shows the characteristic absorption/extinction profile of graphene nanoparticles, enabling a quantitative estimate of the average thickness (with an average number of layers ⟨*N*⟩ = 6), as described in ref 56. Monitoring the UV–vis spectrum of B60 over time, including after drying and redispersion, allows us to verify the absence of restacking phenomena. Multiwavelength Raman spectroscopy provides insight into the spatial confinement of graphene domains and the complex structural features at the sheet edges. In parallel, IR spectroscopy is diagnostic for detecting the native carboxylic groups that decorate the edges of the graphene sheets. The presence of intense IR marker bands corresponding to grafted functional groups or loaded molecules is fundamental for assessing the success and nature of conjugation.^25^

In ref 25 we demonstrated the ability of B60 to form π–π complexes with small molecules featuring extended conjugated π-systems, such as pyrene-carboxylic acid (PyCA). Building on the successful loading of PyCA onto B60, we adopted the same method^57^ to prepare B60 conjugates of compound **8** (**8**@B60). The **8**@B60 system was obtained by mixing B60 and compound **8** in methanol, a solvent that ensures good solubility of **8** and stable dispersions of B60. Upon subsequent addition of water, which poorly solubilizes compound **8**, the formation of **8**@B60 conjugates is promoted and the π–π interactions stabilized. Prolonged sonication prevents aggregation of the conjugates and, if present, disrupts residual B60 clusters. Methanol and unbound **8** were largely removed by an initial centrifugation step, while the effect of additional washing cycles with water was monitored by UV–vis, IR and Raman spectroscopies. These analyses confirmed that the amount of **8** loaded onto B60 remained constant throughout the washing process, and that the resulting dispersion of the conjugates in water was stable. The same procedure was successfully applied to prepare **8-F**@B60 and **8-CF**_**3**_@B60. TEM imaging confirmed that drug loading does not alter the morphology of the nanoparticles. Notably, for the fluorinated conjugates (**8-F**@B60 and **8-CF**_**3**_@B60), TEM was complemented by EDX analysis, which effectively confirmed the presence of fluorine.

Figure 6 displays the TEM images of **B60**, **8**@B60, **8-F**@B60, and **8-CF**_**3**_@B60. The unmodified B60 sample shows a network of GNPs with a uniform size distribution and limited aggregation. Upon loading with compound **8** and fluorinated derivatives of compound **8**, the resulting conjugates retain the original morphology of B60, indicating that the loading process does not significantly alter the nanoparticle structure. At high magnification (10 nm scale), the TEM images of B60, **8**@B60, **8-F**@B60, and **8-CF**_**3**_@B60 reveal short-range lattice fringes within the GNPs. These fringes indicate residual graphitic order and partial crystallinity, consistent with the nanoparticles’ origin by ball milling of graphite. This top-down method typically produces few-layer graphene materials that preserve crystalline graphitic domains, interspersed with amorphous or turbostratic regions caused by shear-induced defects and edge disorder. In particular, the presence of the (002) plane in the diffraction pattern (see insets to Figure 6) confirms a multilayer structure, as previously noted, while the (101) reflection, clearly visible, indicates in-plane periodicity. The (110) reflection is also faintly discernible, but it was not labeled in the insets to preserve readability. The presence of fragmented and curved graphitic areas further reflects the exfoliation and structural disruption introduced during the milling process.

**Figure 6:**
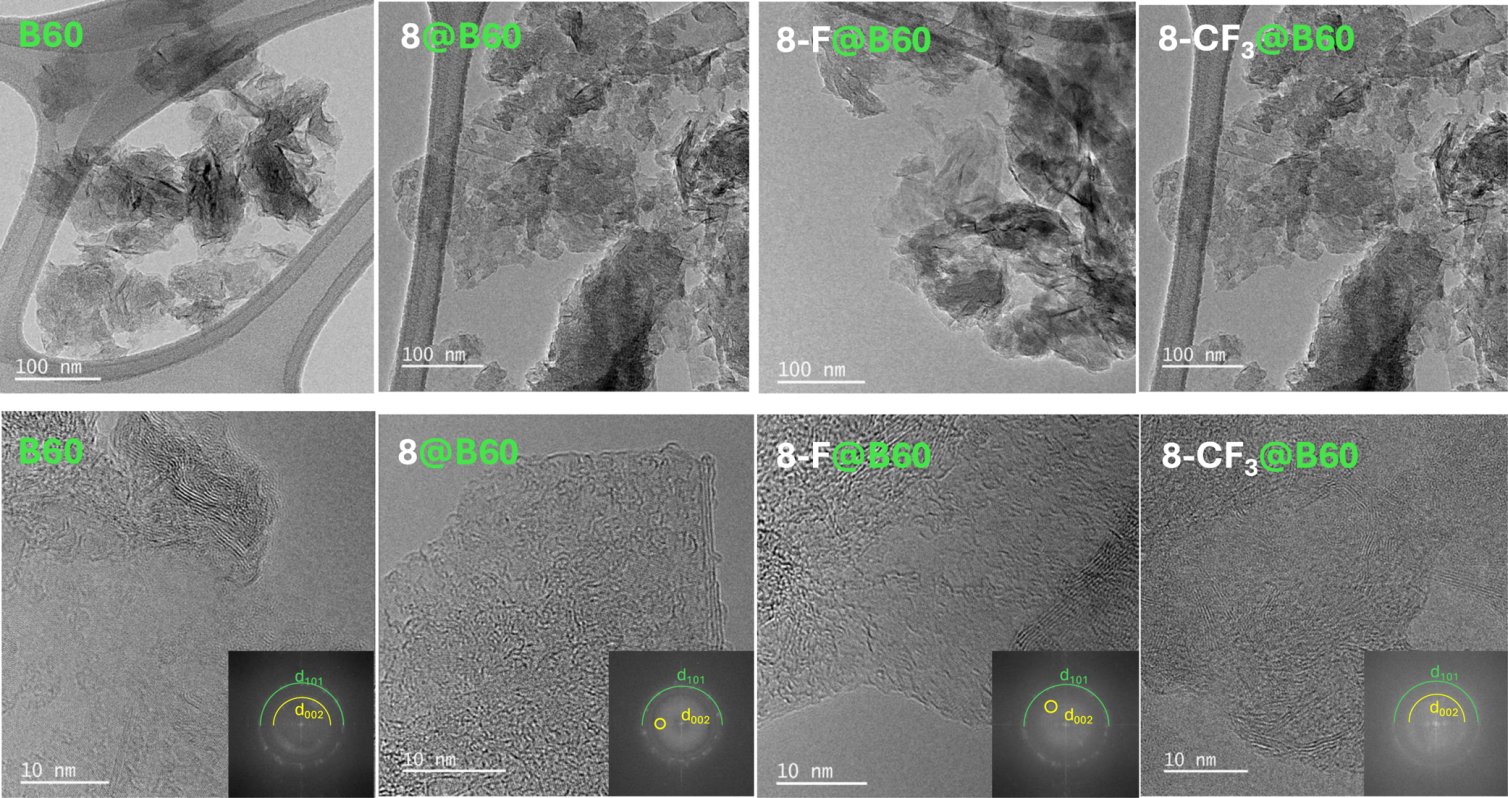
TEM images of B60, **8**@B60, **8-F**@B60, and **8-CF**_**3**_@B60 at low and high magnification. The morphology of the nanoparticles is preserved after loading with compound **8** and its fluorinated derivatives. High-magnification images reveal short-range lattice fringes, indicative of residual graphitic order originating from the ball-milled graphite. The diffraction patterns (see insets) show the presence of the (002) and (101) planes, confirming a multilayer structure and in-plane periodicity. STEM–EDX analysis confirms increasing fluorine content upon functionalization (see Figures S24–S26).

To confirm supramolecular B60 modification, STEM–EDX analysis was conducted to quantify the fluorine content in the samples. Pristine B60 showed a minimal fluorine contamination (0.07 at. %), whereas **8-F**@B60 and **8-CF**_**3**_@B60 exhibited increased fluorine contents of 0.19 at. % and 0.30 at. %, respectively. These values represent the mean of four measurements taken from different areas of each sample. Notably, the increase in fluorine content is not proportional to the number of fluorine atoms in the molecular structure, likely reflecting differences in loading efficiency, molecular orientation or steric hindrance that affect the degree of surface coverage. A representative STEM–EDX analysis is provided in Figures S24–S26.

#### 3.3.2 Spectroscopic Characterization of the Conjugates

Figure 7 presents the UV–vis spectrum of the aqueous dispersions of the **8**@B60 conjugate, while the UV–vis spectra of the **8-F**@B60 and **8-CF**_**3**_@B60 conjugates are presented in Figure S27. In all cases, the HOMO–LUMO transition (band II) appears as a distinct shoulder around 500 nm, superimposed on the characteristic absorption profile of bare B60 in water. For reference, the spectra of the free molecules **8-F** and **8-CF**_**3**_ in solution are reported in Figure S28. The noticeable redshift of band II in the conjugates, compared to the free molecules, confirms that the compounds are effectively loaded onto the B60 surface. However, the band does not extend to the wavelength range typical of the crystalline solids, where intermolecular interactions dominate. This suggests that the molecules are immobilized on B60 as isolated species, without forming aggregates that would replicate the supramolecular organization of the crystal.

**Figure 7:**
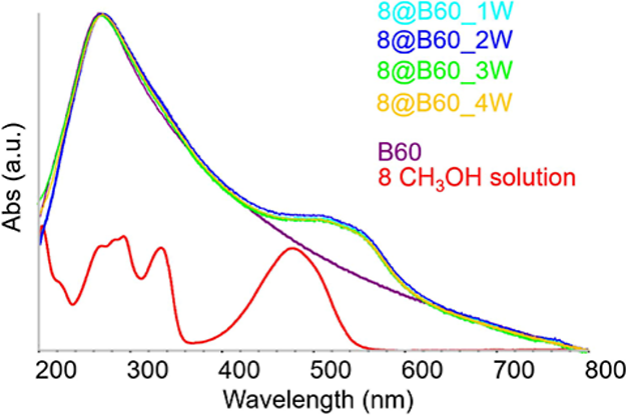
UV–vis spectra of **8**@B60 aqueous dispersions after successive washing steps compared with the spectrum of bare B60 in water (violet line) and of compound **8** in CH_3_OH (red line).

The spectra in Figure 7 indicate that the conjugates dispersions are highly stable, from the second washing step onward. This implies that unbound or weakly associated molecules of compound **8** are almost entirely removed during the initial centrifugation, and additional cycles do not cause desorption or release of the drug. The long-term stability of the aqueous dispersions over time is illustrated in Figure S29.

An approximate estimate of the drug loading efficiency of the conjugation process can be obtained by measuring the concentration of free **8**, or the hydrated derivative, in the H_2_O/CH_3_OH suspension of conjugates, prior to the washing procedure. After centrifugation at 18,000 rpm, the clear supernatant, referred to as “first water” (FW), was analyzed. Its UV–vis spectrum revealed the presence of a few nanoparticles and a distinct absorption peak at 325 nm, attributed to compound **8** in its hydrated form (Figure S30). To quantify the conjugation yield, a reference sample was prepared by dissolving compound **8** in an H_2_O/CH_3_OH mixture at the same initial concentration used for the conjugate formulation. This solution underwent the same processing as that used for the preparation of the **8**@B60 conjugate, resulting in a colorless solution with a strong absorption band at 325 nm. By comparing the intensity (peak height) of this band in the FW spectrum with that of the reference, the fraction of free **8** remaining in the FW after conjugation was estimated relative to the total amount of compound **8** initially used for **8**@B60 preparation. The resulting value, δ = 0.23, corresponds to a conjugation yield η = 0.77.

IR spectra of the conjugates were obtained from solid-state samples using a specular reflection (SR) setup. Figure 8 shows the IR absorption spectrum after Kramers–Kronig transformation of compound **8**@B60; the raw SR spectra and the effect of the transformation are presented in Figure S31. The IR absorption spectra after Kramers–Kronig transformation of compound **8-F**@B60 and **8-CF**_**3**_@B60 are reported in Figure S32. Figure 8 shows a direct comparison between the IR spectrum of **8**@B60 and that of compound **8**. Several characteristic absorption bands of compound **8** are clearly retained in the conjugate, appearing as sharp peaks superimposed on the broad absorption features of B60. The wavenumbers of the observed IR bands corresponding to compound **8** are listed in Table S1, showing close agreement with those of the (*Z*)-2,6-dipyridone tautomer either as a crystal or in chloroform solution. However, the C=O stretching bands of compound **8** are not detectable in the conjugate spectra due to overlap with the broad C=O stretching band of native carboxylic acid groups located at the edges of the graphene sheets.^44^ The IR spectral patterns of the fluorinated derivatives, **8-F** and **8-CF**_**3**_ (Figure S33), closely resemble that of **8**, both in peak positions and in the intensity distribution of the main transitions, with minor deviations attributable to vibrational modes associated with the substituent groups. These observations support the conclusion that, in the solid state, both **8-F** and **8-CF**_**3**_ are also present in the (*Z*)-2,6-dipyridone form. Furthermore, the IR spectra of **8-F**@B60 and **8-CF**_**3**_@B60 (Figure S32) prove that they are loaded onto B60 in this tautomeric form. It is worth noting that graphene complexation of **8**, and its fluorinated derivatives, prevents hydration tipically observed in aqueous environment for the uncomplexed molecules.

**Figure 8:**
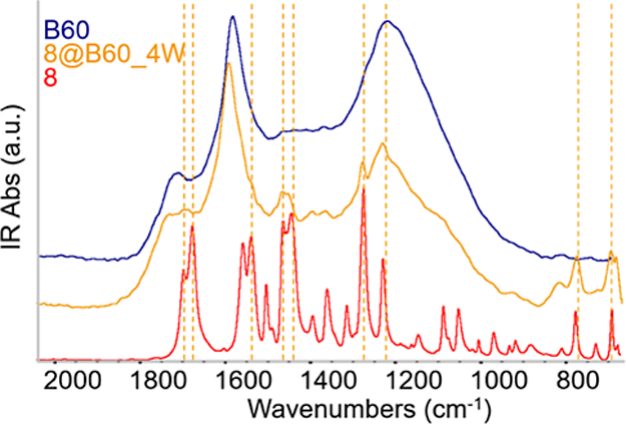
IR spectra of **8**@B60: comparison with the IR spectra of compound **8** and with the spectrum of bare B60.

Figure 9 illustrates the Raman spectrum of **8**@B60 and Figure S34 the Raman spectra of **8-F**@B60 and **8-CF**_**3**_@B60 conjugates. Importantly, the Raman profile of B60 remains unchanged after conjugation, indicating that the graphene-like structure is preserved throughout the functionalization process. As in the IR spectra, several peaks characteristic of the (*Z*)-2,6-dipyridone form are clearly observed in the conjugates through direct comparison with the Raman spectrum of compound **8**. Similarly, the Raman spectra of **8-F**@B60 and **8-CF**_**3**_@B60 show several peaks of **8-F** and **8-CF**_**3**_. Interestingly, high-quality Raman spectra of both bare B60 and the conjugates can be obtained using a red laser excitation source (λ_exc_ = 633 nm). In contrast, under the same excitation conditions, the spectra of the free molecules **8**, **8-F**, and **8-CF**_**3**_ are dominated by strong fluorescence, which masks the Raman signal. To overcome this limitation, their FT-Raman spectra were recorded using a near-infrared laser (λ_exc_ = 1064 nm) and are also reported in Figures 9 and S35. The fluorescence quenching observed upon conjugation provides additional evidence of π–π interaction with the graphene surface.^58,59^

**Figure 9:**
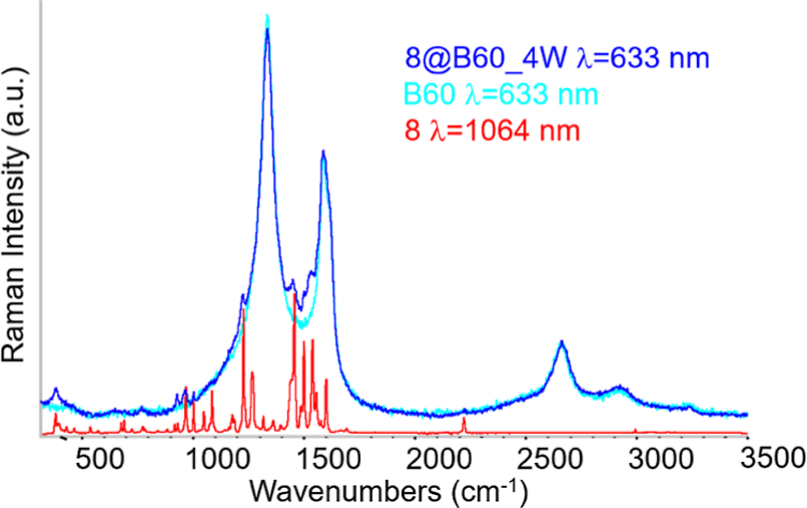
Raman spectrum of **8**@B60: comparison with the Raman spectrum of compound **8** and with the spectrum of bare B60.

A comparison of the FT-Raman spectra of the three molecules is presented in Figure S35. The spectra show close agreement in both the position and intensity of the major Raman bands, with minor differences attributable to vibrational modes involving the fluorinated substituents.

In conclusion, the analysis of the vibrational spectra confirms the successful formation of the three conjugates and demonstrates that the molecules loaded onto B60 adopt the same low-energy structure observed in their pure crystalline form. Figures S36 and S37 provide additional evidence of the conjugates’ stability across multiple washing cycles. From the second washing step onward, the IR and Raman bands attributed to compound **8** remain remarkably consistent in intensity, indicating strong retention of the molecules on the B60 surface. This behavior also highlights the role of B60 as a tautomeric selector, stabilizing the (Z)-2,6-dipyridone form of compound **8** through π–π interactions, even though this is not the dominant species in aqueous solution.

#### 3.3.3 Drug Release Experiments

A series of experiments was conducted to investigate the thermally induced release of compound **8** from **8**@B60 conjugates in aqueous solution. These results demonstrate that UV–vis absorption spectroscopy is a reliable technique for monitoring drug detachment from GNPs. The diagnostic approach relies on two key observations: (i) the appearance of a distinct shoulder in the B60 spectrum around 500 nm, corresponding to the HOMO–LUMO transition of compound **8** when loaded onto B60; and (ii) the tendency of free compound **8**, once released into water, to either transform into a colorless species (Sections 1 and 3.1) or to precipitate due to its limited solubility. In both scenarios the disappearance or weakening of the ∼500 nm shoulder is expected upon drug release. This behavior is clearly illustrated in Figure 10, which reports the UV–vis spectra of an **8**@B60 aqueous dispersion heated to 40 °C and monitored over time. As the temperature-induced release proceeds, the HOMO–LUMO shoulder gradually fades. Concurrently, subtle spectral changes are observed at shorter wavelengths, including the emergence of a weak band around 300 nm. This latter feature may correspond to band I of the free, water-modified (colorless) form of compound **8**. Figure S38 illustrates the release of compounds **8-F** and **8-CF**_**3**_ by heating aqueous dispersions of their conjugates, showing the evolution of the UV–vis spectra with temperature, which parallels the release behavior observed for compound **8**.

**Figure 10:**
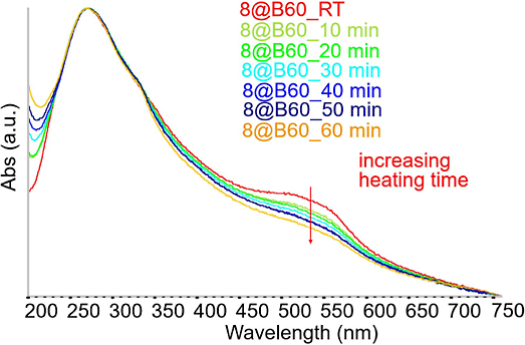
UV–vis of an **8**@B60 aqueous dispersion recorded at ambient temperature (red line) and after heating at 40 °C. Spectra were collected at different time intervals under heating, from 10 to 60 min.

The amount of compound **8** released from the B60 GNPs was determined by heating 1 ml of an aqueous **8**@B60 dispersion (containing 9.45 μg/ml of conjugate) at 70 °C, followed by centrifugation. Although drug release is already observed at 40 °C, the higher temperature was used to ensure complete detachment. UPLC-HRMS analysis of the supernatant (Figure S39) revealed a compound **8** concentration of 1.3 μM, corresponding to a loading of approximately 3.9% w/w relative to the total mass of the **8**@B60 conjugate. For this study, the analysis was limited to the representative case of **8**@B60, although the same procedure can be also extended to its derivatives.

## 4 Conclusions

In this study we present a nanographene-based strategy to mitigate the limitations associated with the poor aqueous solubility of the NEK6 inhibitor compound **8** by embedding it into stable, water-dispersible conjugates. Through noncovalent conjugation of compound **8** and its fluorinated derivatives (**8-F** and **8-CF**_**3**_) onto structurally uniform few-layer graphene nanoparticles (B60), we obtained well-defined nanohybrids (**8**@B60, **8-F**@B60, and **8-CF**_**3**_@B60) that form stable dispersions in water. Spectroscopic and microscopic analyses confirmed efficient drug loading, preservation of B60s structural integrity, and excellent colloidal stability. A central finding of this work is that immobilization on B60 selectively stabilizes the (*Z*)-2,6-dipyridone tautomeric form of compound **8**, identified as the lowest-energy structure in the solid state and in the conjugates, but not dominant in aqueous solution. This underscores the dual role of graphene surfaces, which act not only as carriers but also as (i) tautomeric selectors-favoring the (*Z*)-2,6-dipyridone tautomer-and (ii) conformational selectors-promoting planar, fully π-conjugated structures-enabling a control over molecular stability and release dynamics at the nanoscale. Notably, UPLC-HRMS analysis confirmed that the amount of drug immobilized on B60 corresponds to approximately 3.9% w/w relative to the total conjugate mass, supporting the reliability of our loading protocol and providing a quantitative benchmark for future biological applications. We also uncovered a reversible hydration process of compound **8** in aqueous media, yielding a nonconjugated, colorless species. Although this transformation disrupts the π-electron system and suppresses the visible HOMO–LUMO absorption band, molecular modeling confirms that NEK6 binding affinity is preserved. The pH-dependent and reversible nature of this equilibrium suggests new avenues for environmentally responsive drug delivery. Notably, UV–vis spectroscopy emerged as a powerful tool to monitor both drug loading and thermally triggered release from the graphene nanoparticles’ surface. The incorporation of fluorine atoms in **8-F** and **8-CF**_**3**_ further enabled quantitative surface analysis by STEM–EDX, offering a dual spectroscopic-elemental approach for tracking molecular immobilization.

Looking ahead, several directions merit exploration. In vitro and in vivo studies will be essential to evaluate cellular uptake, intracellular trafficking, and therapeutic efficacy. The excellent stability of the conjugates in water, combined with the responsive release behavior, supports their potential for translation to biological systems. Building on prior demonstrations of B60 peptide functionalization, future work could integrate targeting moieties or stealth coatings using biocompatible polymers or oligomers. Importantly, such modifications should not hinder drug loading by introducing steric or electrostatic interference, as the B60 surfaces need accessibility for π–π interactions. The distinctive ability of B60 to stabilize, in the case of compound **8**, a specific tautomeric and conformational state, while also enabling modular surface functionalization, opens new avenues for the design of next-generation nanocarriers with built-in structural control, targeted delivery, and real-time traceability. These features establish B60-based systems as promising platforms for smart drug delivery, particularly in precision therapies involving NEK6 inhibitor **8** and its active derivatives.

More broadly, the codelivery of multiple therapeutic agents and the prolongation of circulation times remain key challenges in nanomedicine. Our findings contribute to this field by suggesting a design strategy that avoids the need for complex chemical modification of compound **8**, while still achieving effective drug immobilization, stabilization, and release. This streamlined approach helps address synthetic complexity and lays the groundwork for developing practical and scalable solutions in advanced drug delivery. It also provides a comprehensive framework for understanding nanosystem behavior at the molecular level.

## Supplementary Material



## Notes

Includes detailed characterization and computational analyses supporting the structural, spectroscopic, and functional properties of compound **8** and its fluorinated derivatives (**8-F** and **8-CF**_**3**_): synthesis and structural characterization synthetic procedures for the preparation of compounds **8**, **8-F**, and **8-CF**_**3**_. ^1^H and ^13^C NMR spectra, HRMS, and UPLC chromatograms of compounds **8**, **8-F**, and **8-CF**_**3**_. NOESY-NMR of compound **8** to confirm the (*Z*)-configuration. DFT-optimized structures for key (*E*)-tautomers and of the 2,6-dihydroxy derivative. Spectroscopic analyses TDDFT-simulated UV–vis spectra of different tautomers and of the hydrated 2,6-dihydroxy derivative. DFT-computed IR and Raman spectra for key (*Z*)-tautomers, with comparison to experimental spectra in solution and solid state. Assignment tables for IR and Raman bands, and vibrational eigenvector visualizations. Hydration and reactivity studies UV–vis spectrum of compound **8** recorded as a function of pH. UV–vis and ^1^H NMR spectra of the hydrated 2,6-dihydroxy derivative. ESI-MS with UV–vis detection of hydration and methanol addition products. Fukui function analysis to identify electrophilic sites for water addition. Conjugate characterization UV–vis spectra of aqueous dispersions of **8-F**@B60 and **8-CF**_**3**_@B60. UV–vis spectrum of the supernatant after centrifugation of the **8**@B60 dispersion. IR spectra of **8-F**@B60 and **8-CF**_**3**_@B60. Raman spectra of 8-F@B60 and 8-CF_3_@B60. STEM–EDX mapping of B60, **8-F**@B60, and **8-CF**_**3**_@B60. UV–vis, IR, and Raman spectra of 8@B60 and fluorinated conjugates, including stability across washing steps. UV–vis spectra of aqueous dispersions **8-F**@B60, and **8-CF**_**3**_@B60 after thermal releasing. UPLC-HRMS quantification of released compound **8** after thermal treatment. Molecular dynamics and docking time evolution of Cα-rmsd and ligand rmsd (rmsd_lig_) for different (*Z*)-tautomers and the hydrated 2,6-dihydroxy derivative within NEK6 binding pocket (PDF)
