# Supplementary material for: Drug Loading and Release: Development and Characterization of a Novel Therapeutic Agent-Nanographene Conjugate
Source: J Phys Chem B. 2025 Sep 1;129(36):9097–112. doi: 10.1021/acs.jpcb.5c04238 (PMC12434661; doi:10.1021/acs.jpcb.5c04238)
Supplement: Supplementary file 1 [file jp5c04238_si_001.pdf]

Supporting Information

for

Drug Loading and Release: Development and Characterization of a Novel Therapeutic Agent-Nanographene Conjugate

Kaiyue Hu,[1] Agnese Pavan,[2] Alessandro Semeraro,[3] Alberto Ongaro,[2] Luigi Brambilla,[1] Maria Cristina De Rosa,[4] Matteo Tommasini,[1] Chiara Castiglioni,[1]* Michele Maggini[2,5]

[1] Dipartimento di Chimica, Materiali e Ingegneria Chimica Giulio Natta, Politecnico di Milano, Piazza Leonardo da Vinci 32, 20133, Milano, Italy

[2] Dipartimento di Scienze Chimiche, Università di Padova, Via F. Marzolo 1, 35131 Padova, Italy

[3] Dipartimento di Chimica e Tecnologie del Farmaco, Sapienza, Università di Roma, P.le A. Moro 5, 00185, Roma, Italy.

[4] Istituto di Scienze e Tecnologie Chimiche Giulio Natta (SCITEC)-CNR, Roma, L.go F. Vito 1, 00168, Roma, Italy

[5] Istituto di Chimica della Materia Condensata e di Tecnologie per l'Energia (ICMATE)-CNR, Padova, Corso Stati Uniti 4, 35127, Padova, Italy

TABLE OF CONTENT

Synthetic procedures for the preparation of inhibitor **8** and its derivatives **8-F** and **8-CF$_3$**.

Figure S1. ^{1}H NMR and ^{13}C NMR spectra of compound **8**.
Figure S2. HRMS spectra and UPLC chromatogram of compound **8**.
Figure S3. ^{1}H NMR and ^{13}C NMR spectra of compound **8-F**.
Figure S4. HRMS spectra and UPLC chromatogram of compound **8-F**.
Figure S5. ^{1}H NMR and ^{13}C NMR spectra of compound **8-CF$_3$**.
Figure S6. HRMS spectra and UPLC chromatogram of compound **8-CF$_3$**.
Figure S7. NOESY-NMR (400 MHz, DMSO-d_6) of compound **8**.
Figure S8 DFT-optimized structures of two low-energy conformers of (*E*)-2,6-dipyridone.
Figure S9. UV-vis TDDFT spectra of different isomers/tautomers of **8.**
Figure S10. Comparison of the calculated (DFT) IR spectra of the different lowest energy *Z* tautomers/conformers of compound **8**.
Figure S11. Comparison of the calculated (DFT) Raman spectra of the different lowest energy *Z* tautomers/conformers of compound **8** in the fingerprint region.
Figure S12. UV-vis spectra of compounds **8-F** and **8-CF$_3$** in CH_3OH recorded at increasing time intervals.
Figure S13. Comparison between the UV-vis spectra of compound **8** in reagent-grade CH_3OH and in dry CH_3OH under inert atmosphere at increasing time intervals.
Figure S14. UV-vis spectra of compound **8** in H_2O:DMSO 90:10 v/v at increasing time intervals and UV-vis spectrum of the same solution before and after the addition of acetic acid.
Figure S15. The Fukui function $f_+(\mathbf{r}) = n_{anion}(\mathbf{r}) - n_{neutral}(\mathbf{r})$ computed as the difference between the electron density of the anion of **8** and its neutral form.
Figure S16. UV-vis spectra of compound **8** (6.9 μM final concentration) recorded immediately after addition to aqueous buffer solutions at different pH values (4-9).
Figure S17. ^{1}H NMR (400 MHz, DMSO-d_6) of the hydrated adduct of compound **8** (2,6 dihydroxy derivative).
Figure S18. FFI ESI-MS analysis of compound **8** coupled with UV-vis detection.
Figure S19. DFT-optimized structure of the 2,6-dihydroxy derivative.
Figure S20. UV-vis TDDFT spectrum of the isolated hydration product of compound **8** (2,6-dihydroxy derivative).
Figure S21. IR spectra of compound **8** in $CHCl_3$ and in the solid state (survey).

Figure S22. FT-Raman spectra of **8** in $CHCl_3$ and in the solid state (survey). Exciting laser line 1064 nm.
Figure S23. Comparison of the experimental IR and Raman spectra of compound **8** in chloroform solution, with the DFT computed spectra for different tautomers/isomers of compound **8**.
Figure S24. STEM-EDX elemental mapping of pristine B60.
Figure S25. STEM-EDX elemental mapping of **8-F**@B60.
Figure S26. STEM-EDX elemental mapping of **8-CF$_3$**@B60.
Figure S27. UV-vis spectra of aqueous dispersions **8-F**@B60 and **8-CF$_3$**@B60, compared with the spectrum of bare B60 in water and of the free molecule in CH_3OH.
Figure S28. UV-vis spectra of compounds **8**, **8-F** and **8-CF$_3$** in CH_3OH.
Figure S29. UV-vis spectra of **8**@B60, **8-F**@B60 and **8-CF$_3$**@B60 water dispersion: evolution over time.
Figure S30. UV-vis spectrum of the supernatant (FW) after centrifugation of the **8**@B60 dispersion, before the washing steps. Comparison with the spectrum of the CH_3OH/H_2O solution of compound **8**, subjected to the same procedure.
Figure S31. IR spectrum of B60 and **8**@B60: Specular Reflection (SR) spectrum and absorption spectrum after Kramers-Kronig transformation.
Figure S32. IR spectra of **8-F**@B60 **(a)** and **8-CF$_3$**@B60 **(b)**: comparison with the IR spectra of **8-F**, **8-CF$_3$** and of bare B60.
Figure S33. Infrared spectra of **8**, **8-F** and **8-CF$_3$**. Solid-state samples.
Figure S34. Raman spectra of **8-F**@B60 **(a)** and **8-CF$_3$**@B60 **(b)**: comparison with the Raman spectra of compounds **8-F** and **8-CF$_3$** and with the Raman spectrum of bare B60.
Figure S35. FT-Raman spectra (λ_{exc} = 1064 nm) of **8**, **8-F** and **8-CF$_3$**. Solid-state samples.
Figure S36. IR spectra of **8**@B60 after several washing cycles.
Figure S37. Raman spectra of **8**@B60 after several washing cycles.
Figure S38. Drug release of: **8-F**@B60 and **8-CF$_3$**@B60: UV-vis spectra of aqueous dispersions of the conjugates recorded at ambient temperature and after heating.
Figure S39. UPLC-HRMS (ESI+) analysis of the supernatant after heating an aqueous dispersion of **8**@B60 at 70 °C for 1 hour followed by centrifugation.

Scheme S1. Time evolution of the Cα-RMSD (blue) and $RMSD_{lig}$ (red) of different (*Z*)-tautomers of compound **8** and of its hydrated 2,6-dihydroxy derivative.

Table S1. Wavenumbers of the IR transitions of **8**: experimental data from a $CHCl_3$ solution, solid-state sample, and of the **8**@B60 conjugate; DFT-calculated wavenumbers and IR intensities.
Table S2. Raman transitions wavenumbers of compound **8**: experimental data for $CHCl_3$ solution, solid-state sample, and the **8**@B60 conjugate. DFT-calculated wavenumbers and Raman activities.

Comments on the DFT-predicted vibrational spectra of the Z tautomers of compound 8

Scheme S2. Sketch of the DFT computed vibrational eigenvectors associated to C=O stretching modes of (*Z*)-6-dipyridone and (*Z*)-2,6-dipyridone-c1 tautomers of **8**.

Synthesis of inhibitor **8** and its derivatives **8-F** and **8-CF$_3$**.

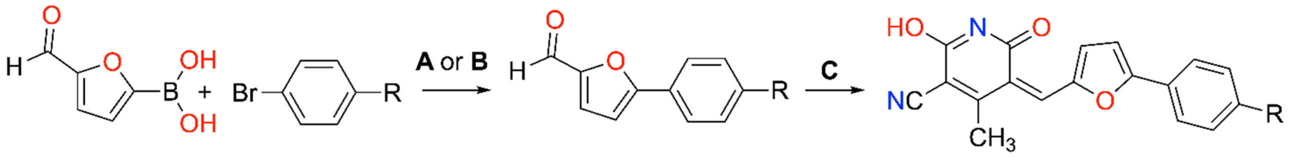

Reagents and conditions:
A: $Pd(PPh_3)_2Cl_2$, EtOH, DMF, Na_2CO_3, ΔT
B: $Pd(PPh_3)_2Cl_2$, EtOH, THF, CuBr, Na_2CO_3, ΔT
C: 2-pyridone **1**, β-alanine, AcOH, ΔT

2, R = H, commercial
3, R = F, 60%
4, R = CF_3, 25%

8, R = H, 92%
8-F, R = F, 94%
8-CF$_3$, R = CF_3, 90%

Compound 8. 5-phenylfuran-2-carbaldehyde (0.18 g, 1.0 mmol, 1.0 equiv.), compound **1** (0.30 g, 2.0 mmol, 2.0 equiv.) and β-alanine (0.18 g, 2.0 mmol, 2.0 equiv.) were dissolved in acetic acid (4.0 ml) and heated at 90 °C for 90 minutes. The precipitated was filtered and washed with water and dried under vacuum to obtain **8** as an orange/red solid (0.28 g, 0.9 mmol, 92%). ^{1}H NMR (400 MHz, DMSO-*d6*) δ (ppm): 11.72 (s, 1H), 8.65 (d, J=2.7 Hz, 1H), 8.02-7.99 (m, 2H), 7.88 (s, 1H), 7.59-7.49 (m, 4H), 2.63 (s, 3H). ^{13}C NMR (101 MHz, DMSO-*d6*) δ (ppm): 162.81, 162.24, 161.64, 161.30, 151.16, 135.97, 131.33, 130.97, 129.79, 128.72, 125.94, 118.49, 115.86, 112.83, 103.27, 19.67. HRMS (ESI) for ($C_{18}H_{12}N_2O_3$): *m/z* calc. 305.09207 $[M+H]^+$, found: 305.09787.

Compound 8-F. 5-(4-fluorophenyl)furan-2-carbaldehyde (0.10 g, 0.5 mmol, 1.0 equiv.), compound **1** (0.16 g, 1.1 mmol, 2.0 equiv.) and β-alanine (0.09 g, 1.1 mmol, 2.0 equiv.) were dissolved in acetic acid (2.5 ml) and heated at 90 °C for 90 minutes. The precipitated was filtered and washed with water and dried under vacuum to obtain **8-F** as an orange/red solid (0.16 g, 0.5 mmol, 94%). ^{1}H NMR (400 MHz, DMSO-*d6*) δ (ppm): 11.71 (s, 1H), 8.63 (d, J=4.0 Hz, 1H), 8.08-8.05 (m, 2H), 7.87 (s, 1H), 7.51 (d, J=4.1 Hz, 1H), 7.45-7.40 (m, 2H), 2.62 (s, 3H). ^{13}C NMR (101 MHz, DMSO-*d6*) δ (ppm): 164.94, 162.78, 162.18, 161.62, 160.36, 151.14, 135.88, 131.37, 128.45, 128.37, 125.49, 125.46, 118.46, 117.11, 115.83, 112.66, 19.65. HRMS (ESI) for ($C_{18}H_{11}F_1N_2O_3$): *m/z* calc. 323.08265 $[M+H]^+$, found: 323.08671.

Compound 8-CF$_3$. 5-(4-(trifluoromethyl)phenyl)furan-2-carbaldehyde (0.05 g, 0.2 mmol, 1.0 equiv.), compound **1** (0.06 g, 0.4 mmol, 2.0 equiv.) and β-alanine (0.04 g, 0.4 mmol, 2.0 equiv.) were dissolved in acetic acid (1.0 ml) and heated at 90 °C for 90 minutes. The precipitated was filtered and washed with water and dried under vacuum to obtain **8-CF$_3$** as a red solid (0.07 g, 0.5 mmol, 90%).^{1}H NMR (400 MHz, DMSO-*d6*) δ (ppm): 11.77 (s, 1H), 8.61 (d, J=4.0 Hz, 1H), 8.20-8.18 (m, 2H), 7.93-7.89 (m, 3H), 7.67 (d, J=4.0, 1H), 2.63 (s, 3H). ^{13}C NMR (101 MHz, DMSO-*d6*) δ (ppm): 162.77, 162.12, 161.54, 158.77, 151.78, 135.96, 132.40, 130.36, 129.96, 126.73, 126.69, 126.35, 119.75, 115.69, 114.33, 104.04, 19.67. HRMS (ESI) for ($C_{19}H_{11}F_3N_2O_3$): *m/z* calc. 373.07218 $[M+H]^+$, found: 373.08094.

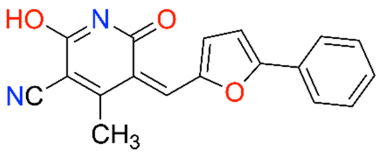

8

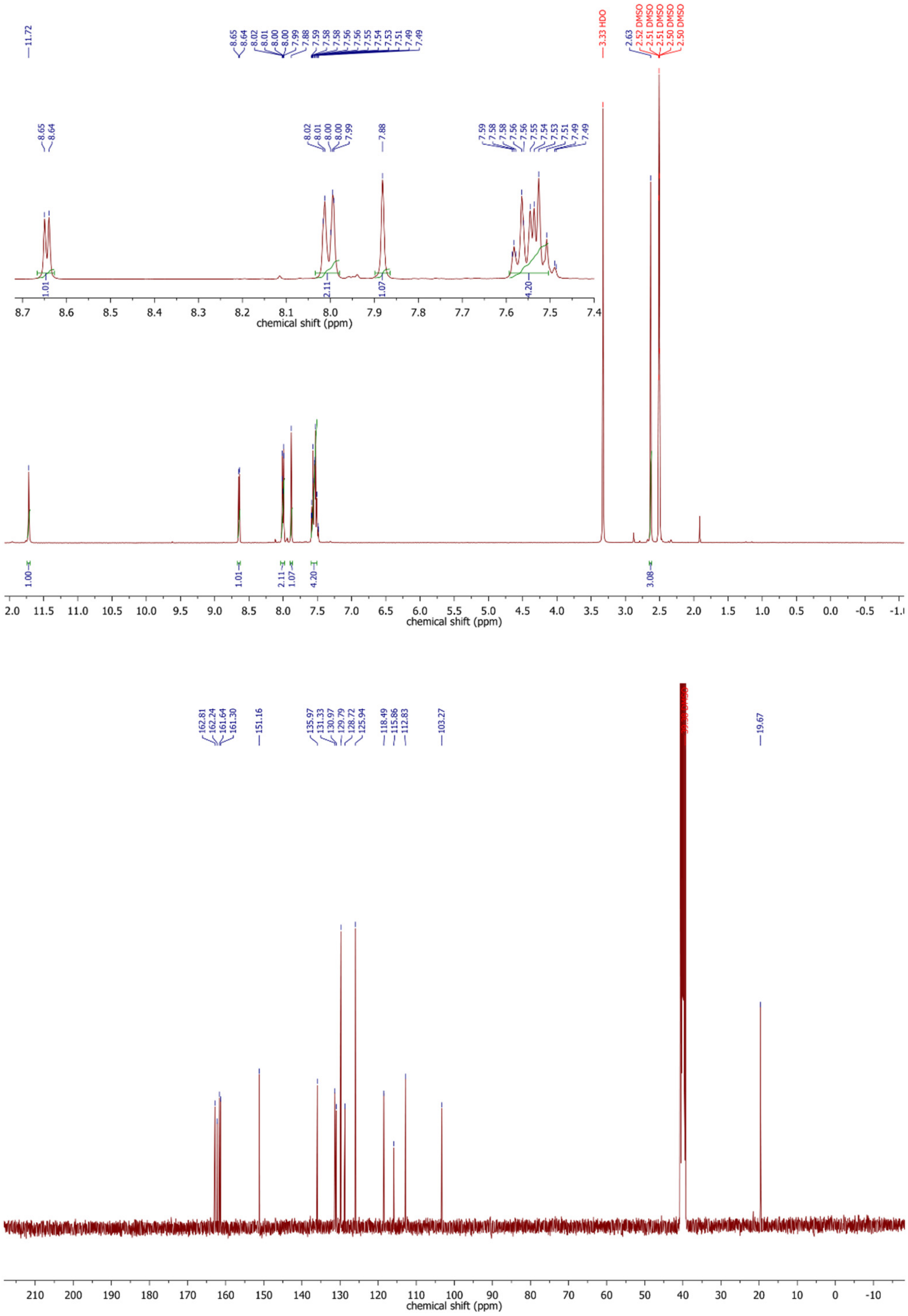

Figure S1. Top: ^{1}H NMR (400 MHz, DMSO-*d6*); bottom: ^{13}C NMR (101 MHz, DMSO-*d6*) of compound **8**.

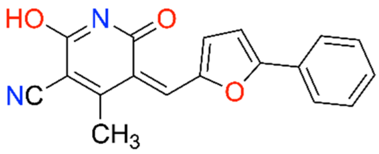

8

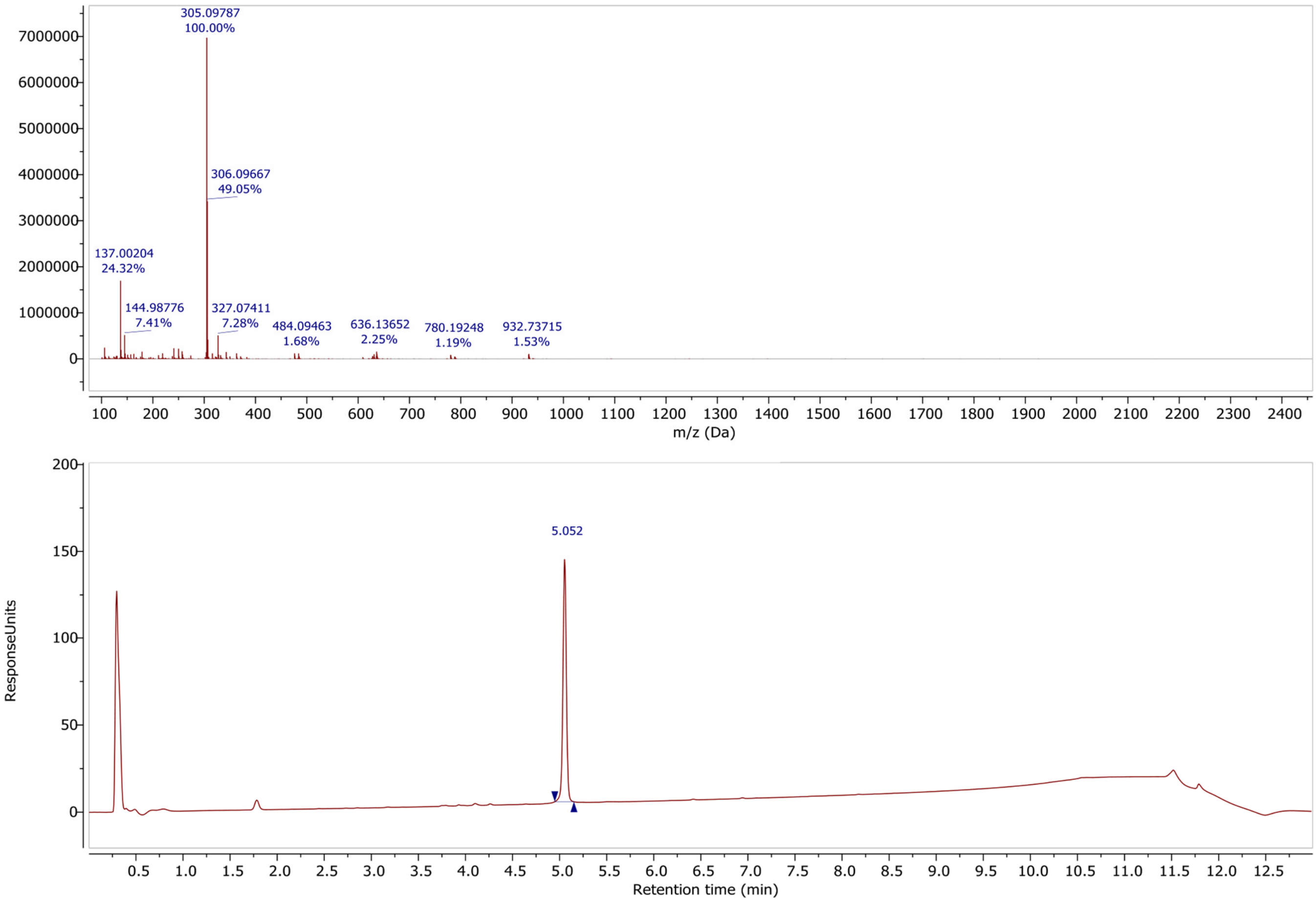

Figure S2. Top: HRMS (ESI$^+$); bottom: UPLC chromatogram recorded at 254 nm of compound **8** ($C_{18}H_{12}N_2O_3$). Calc. $[M+H]^+$ 305.09207, found 305.09787.

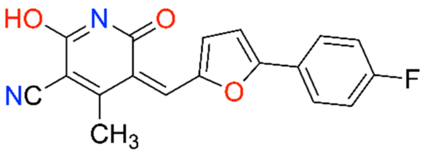

8-F

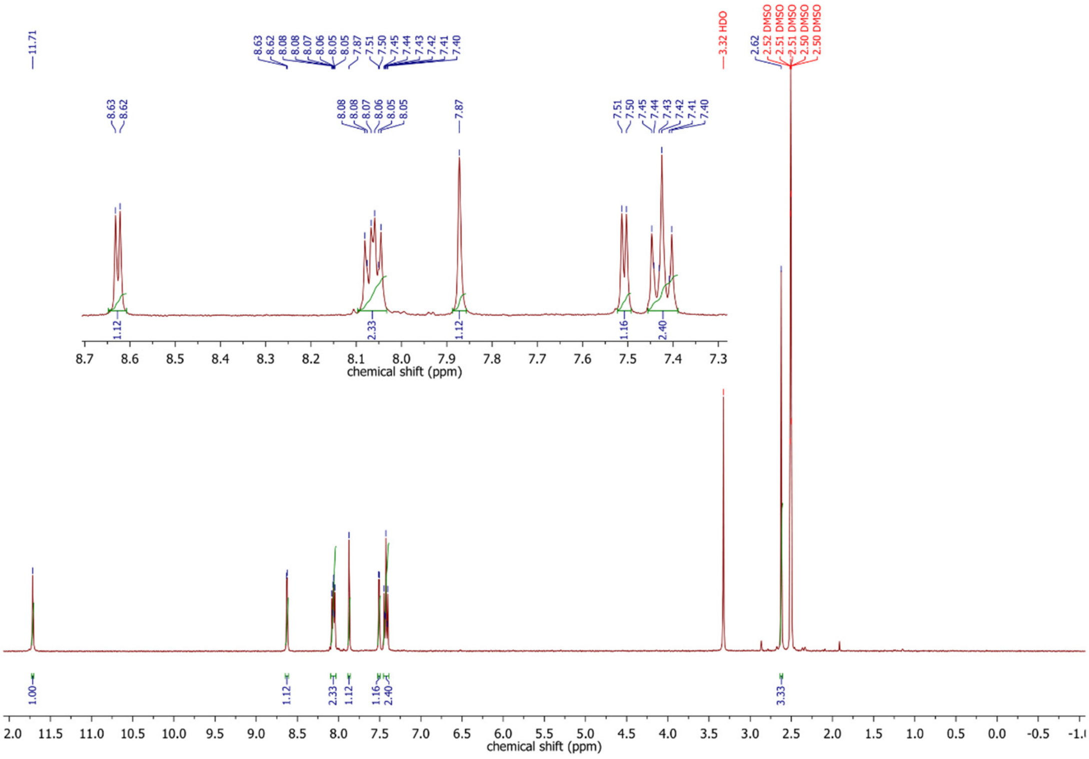

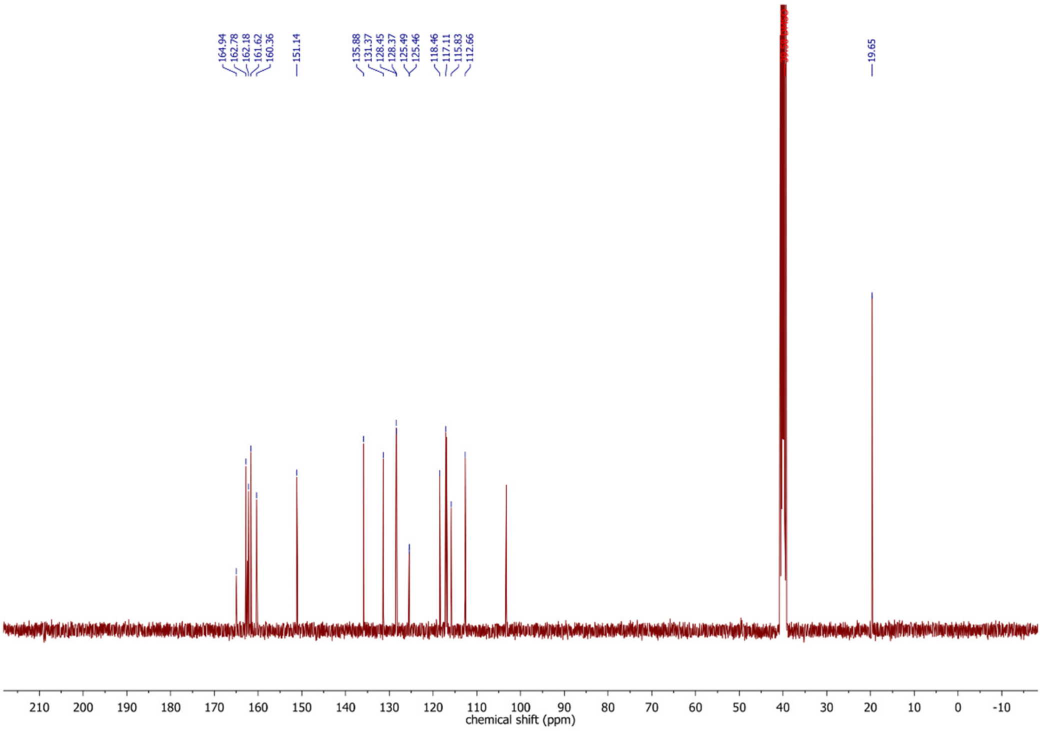

Figure S3. Top: ^{1}H NMR (400 MHz, DMSO-d6); bottom: ^{13}C NMR (101 MHz, DMSO-*d6*) of **8-F**.

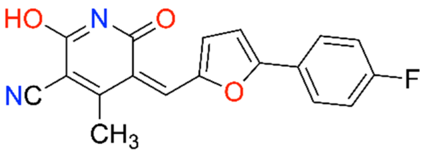

8-F

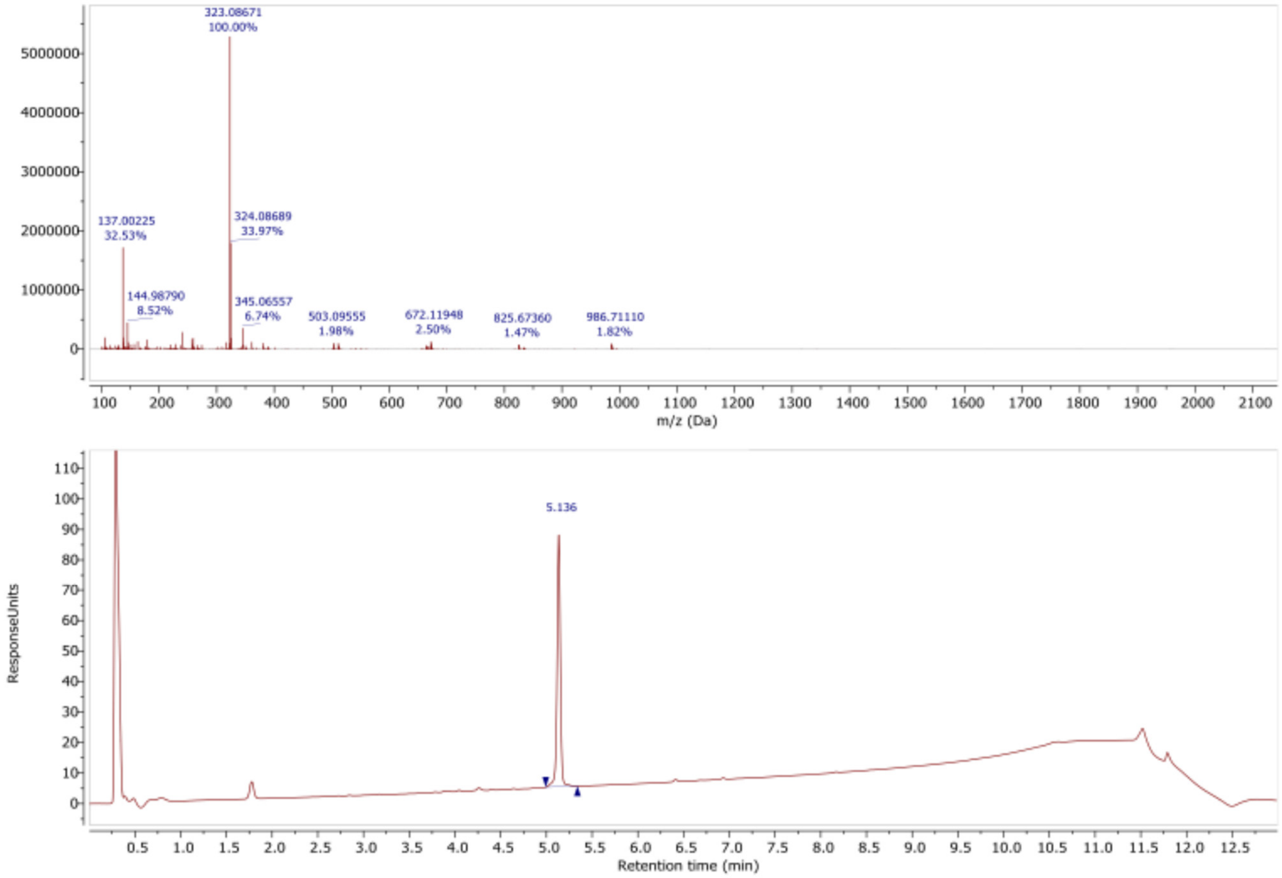

Figure S4. Top: HRMS (ESI^+); bottom: UPLC chromatogram recorded at 254 nm of compound **8-F** ($C_{18}H_{11}F_1N_2O_3$). Calc. $[M+H]^+$ 323.08265, found 323.08671.

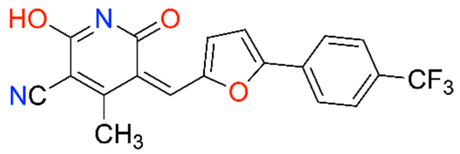

8-CF$_3$

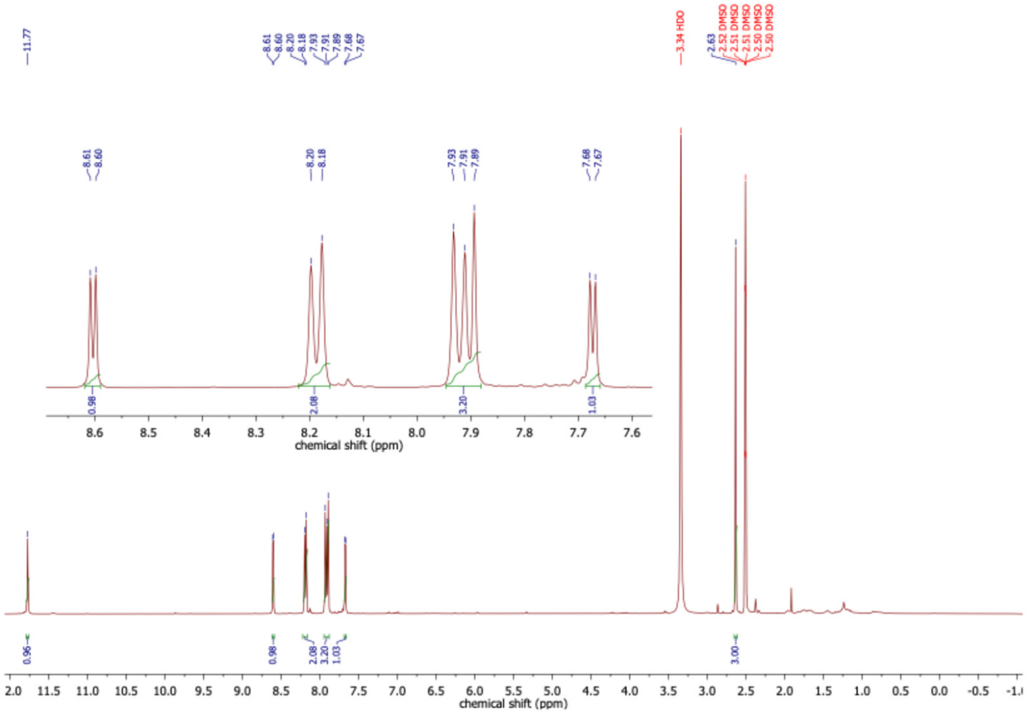

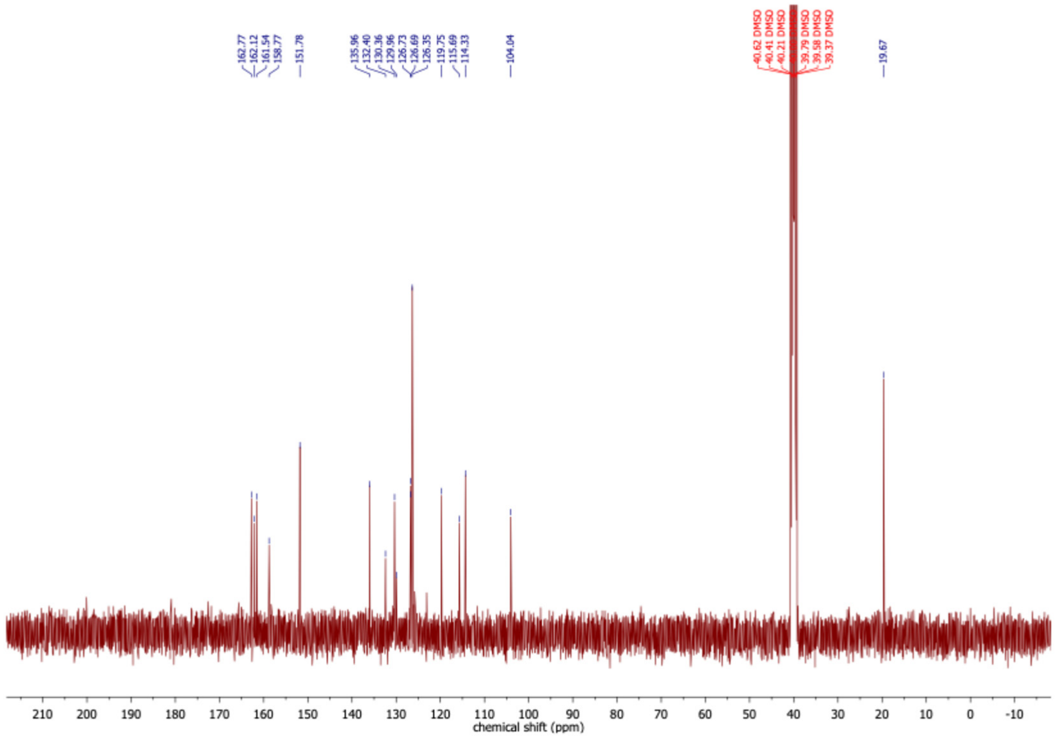

Figure S5. Top: ^{1}H NMR (400 MHz, DMSO-*d6*); bottom: ^{13}C NMR (101 MHz, DMSO-*d6*) of compound **8-CF$_3$**.

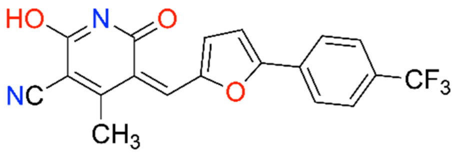

8-CF$_3$

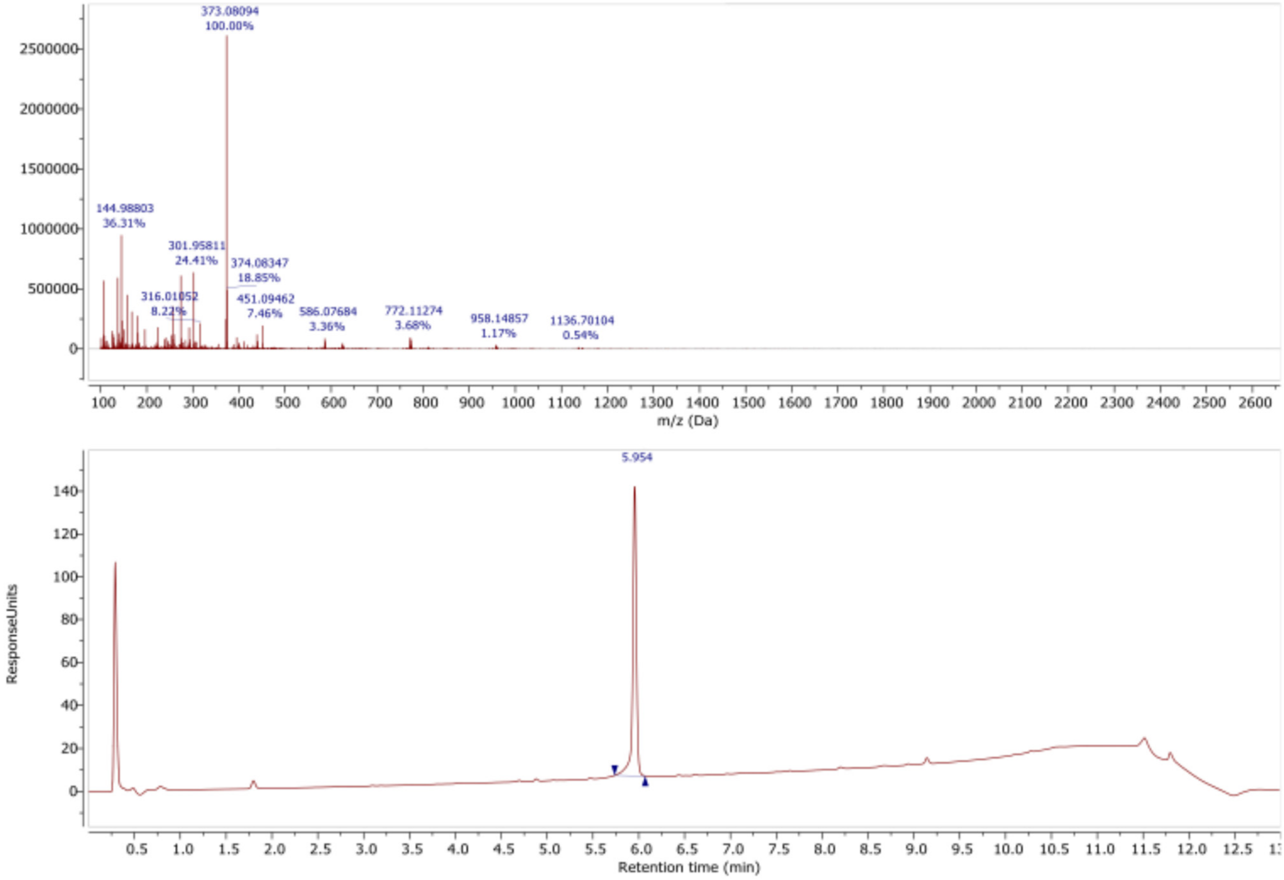

Figure S6. HRMS (ESI$^+$); bottom: UPLC chromatogram recorded at 254 nm of compound **8-CF$_3$** ($C_{19}H_{11}F_3N_2O_3$). Calc. $[M+H]^+$ 373.07218, found 373.08094.

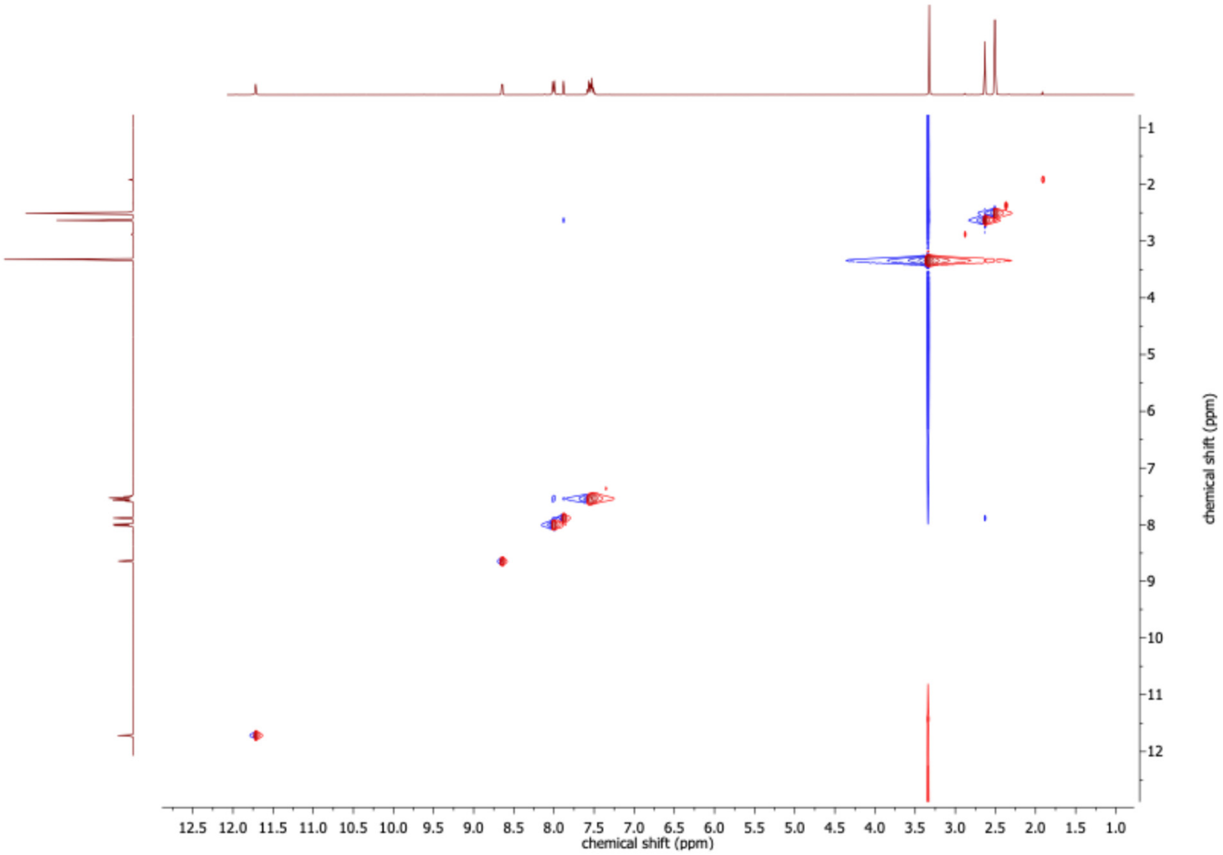

Figure S7. NOESY-NMR (400 MHz, DMSO-d_6) of compound **8**.

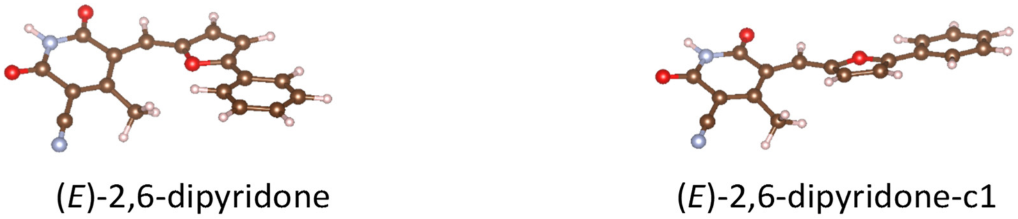

Figure S8. DFT-optimized structures of two low-energy conformers of (*E*)-2,6-dipyridone. Calculations were performed on the isolated molecules at the B3LYP/6-311++G(d,p) level, including D3BJ empirical dispersion.

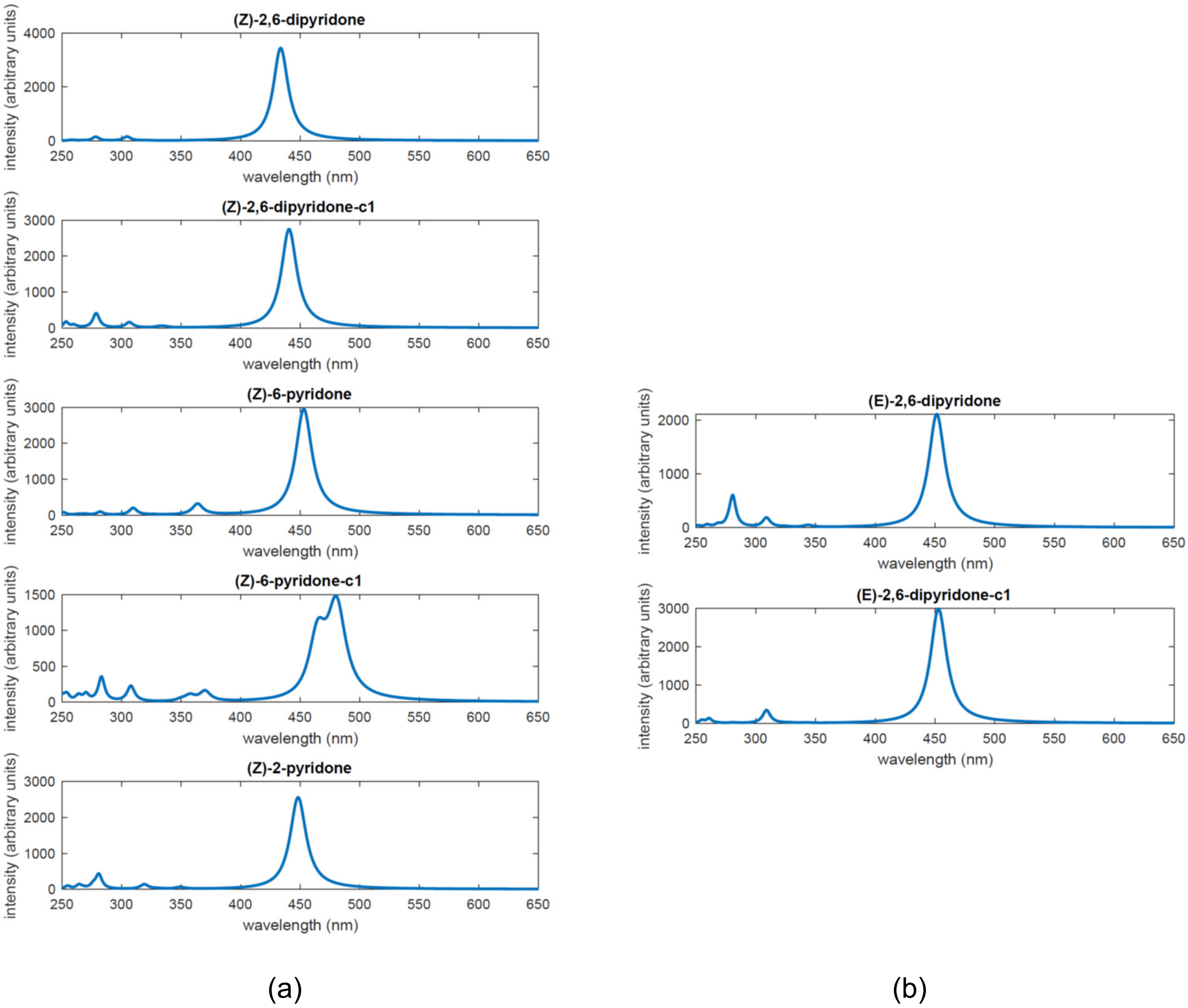

Figure S9. UV-vis TDDFT spectra of different isomers/tautomers of **8.** The calculations have been performed on isolated molecules, corresponding to the structures of Figure 1 (panel a), and Figure 2 (panel b) of the manuscript.

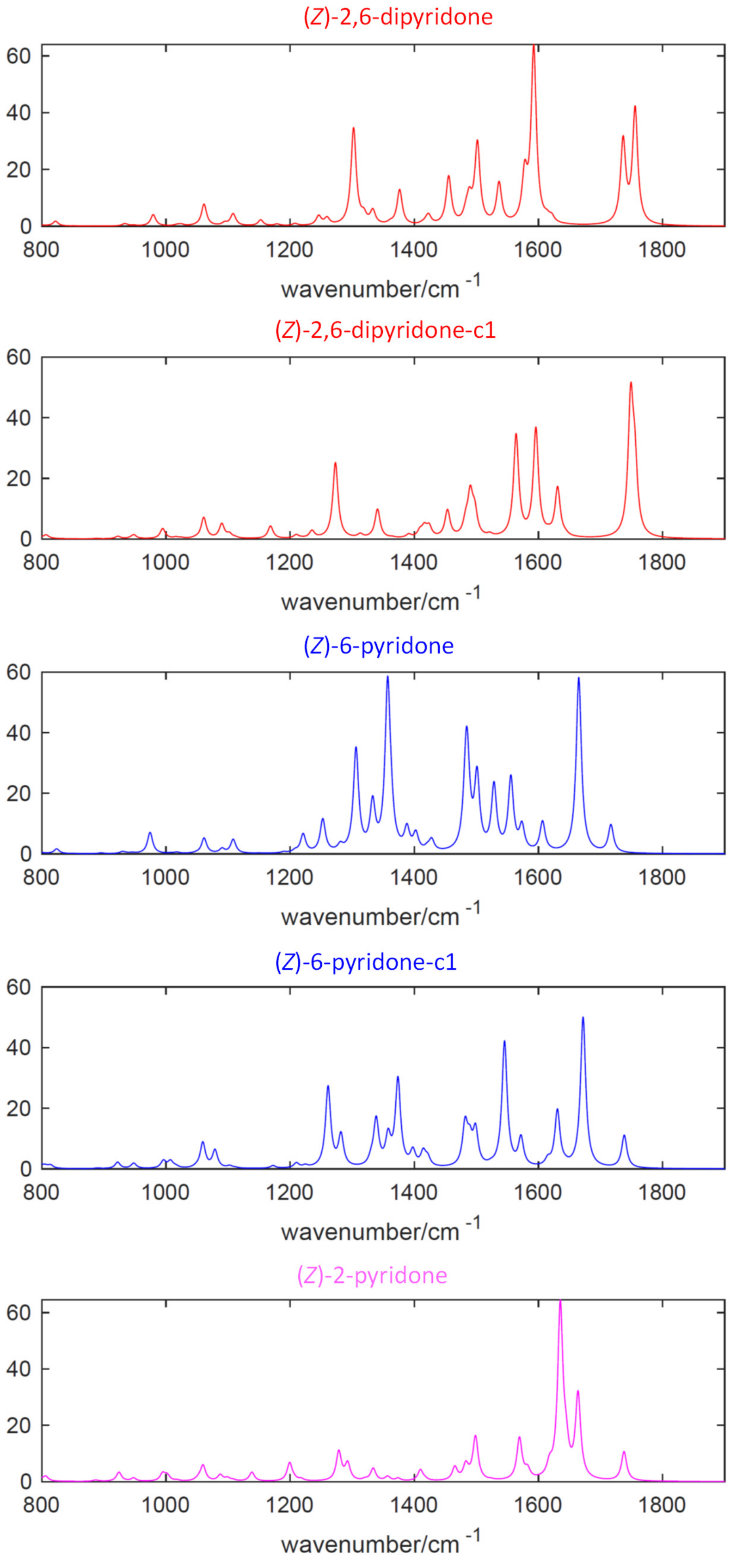
(Z)-2,6-dipyridone
(Z)-2,6-dipyridone-c1
(Z)-6-pyridone
(Z)-6-pyridone-c1
(Z)-2-pyridone
0
20
40
60
800
1000
1200
1400
1600
1800
wavenumber/cm -1

(a)

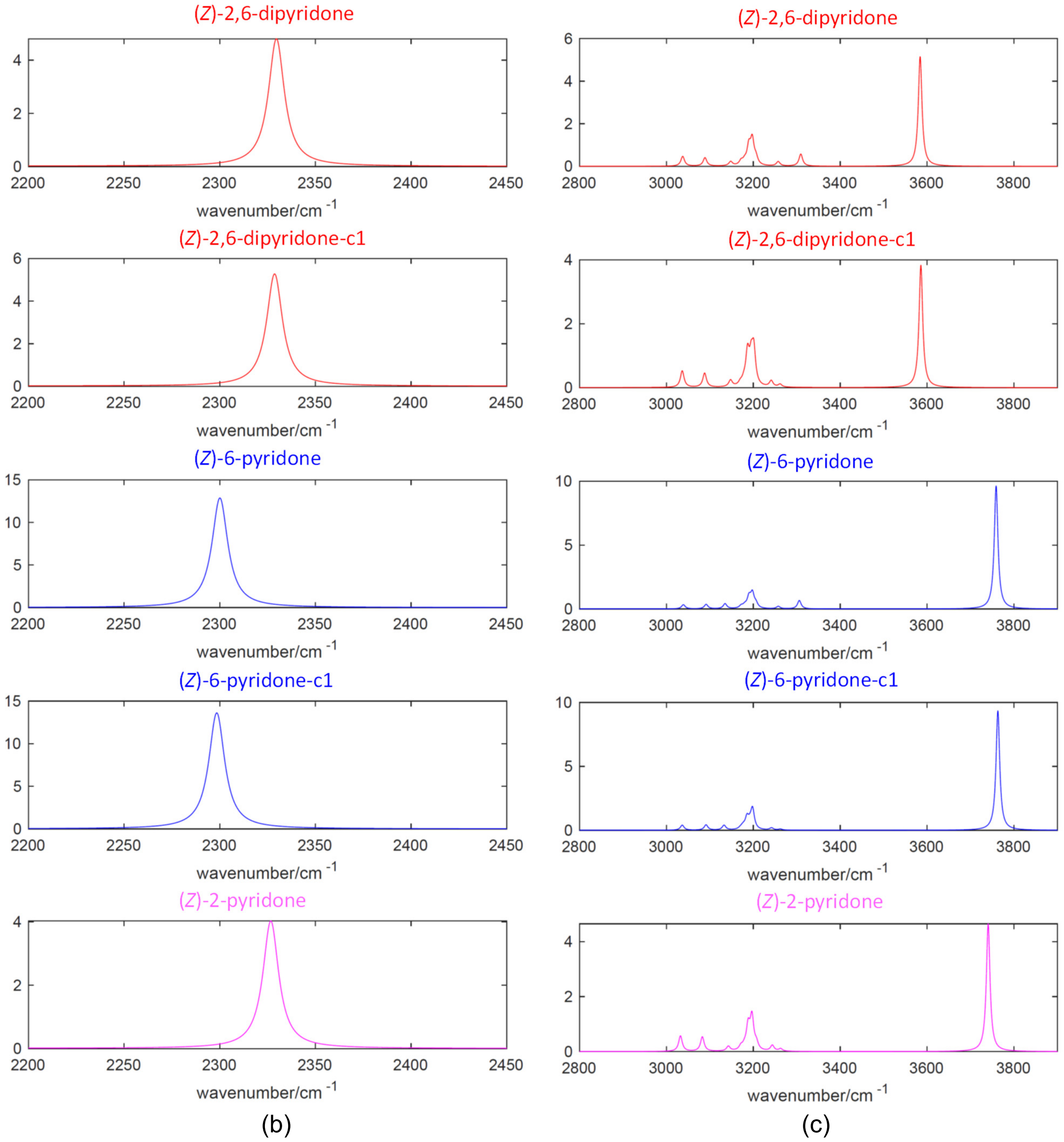

Figure S10. Comparison of the calculated (DFT) IR spectra of the different lowest energy *Z* tautomers/conformers of compound **8**. Panel (a): Fingerprints region from 800-1900 cm^{-1}; Panel (b): CN stretching region; Panel (c): High wavenumber region (region 2800 – 3900 cm^{-1}) showing OH, NH, and CH stretching transitions.

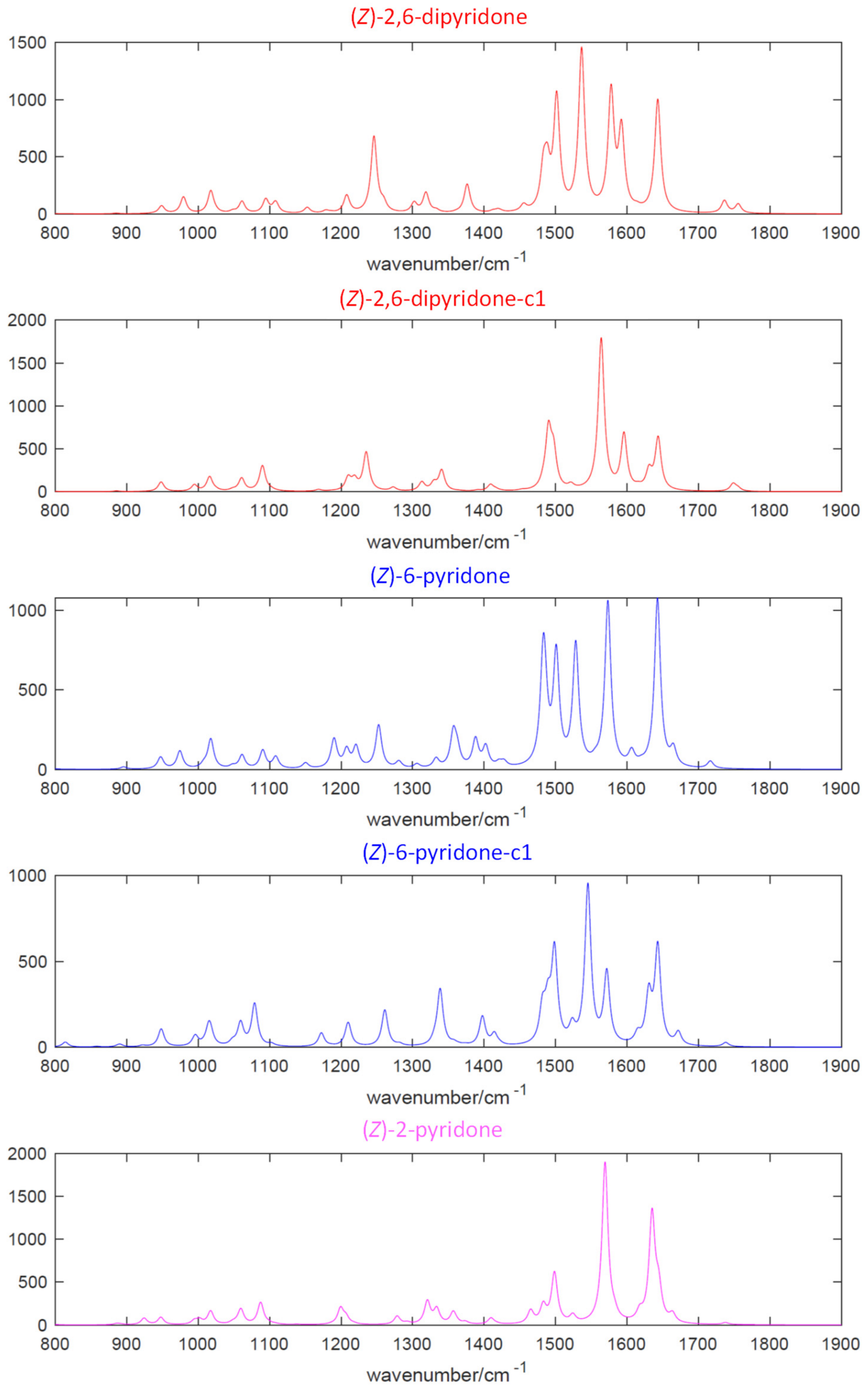

Figure S11. Comparison of the calculated (DFT) Raman spectra of the different lowest energy (*Z*)-tautomers/conformers of compound **8** in the fingerprint region from 800-1900 cm^{-1}.

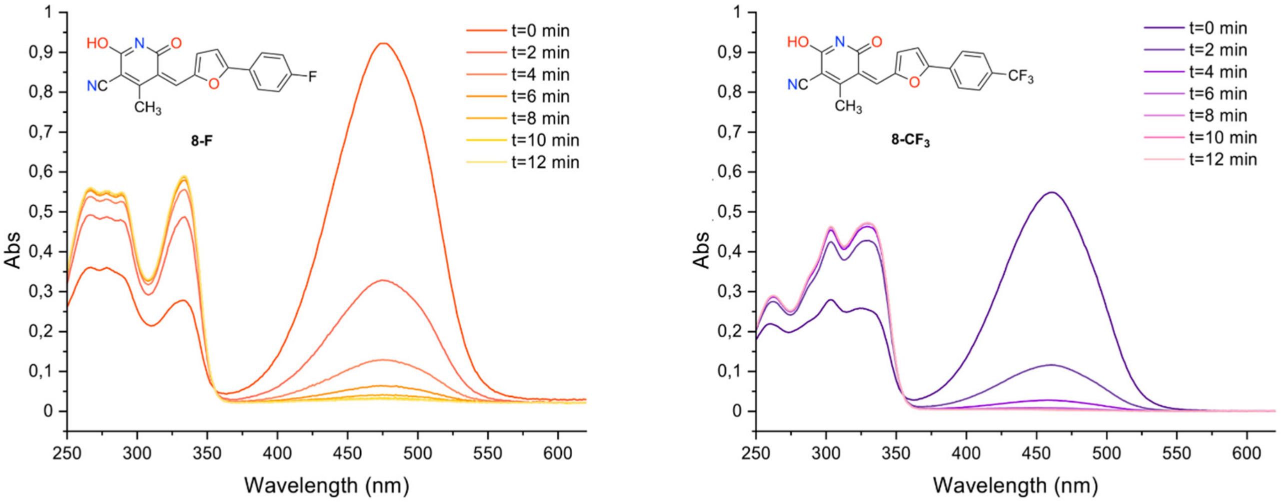

Figure S12. UV-vis spectra of compounds **8-F** (left panel) and **8-CF$_3$** (right panel) in CH_3OH recorded immediately after preparation and at increasing time intervals.

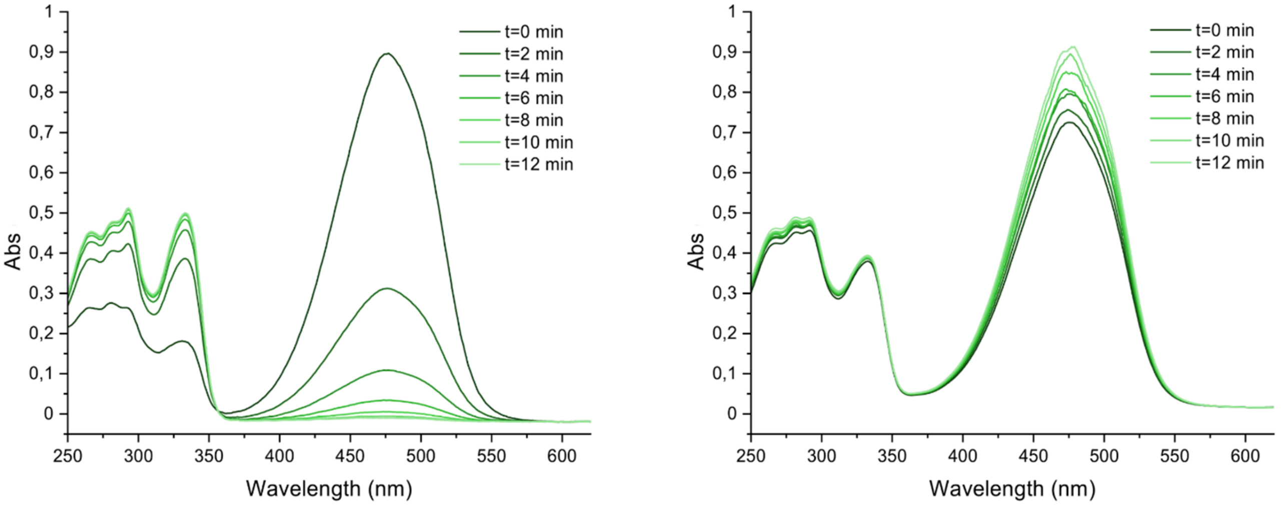

Figure S13. Comparison between the UV-vis spectra of compound **8** in reagent-grade CH_3OH (left panel) and in dry CH_3OH under inert atmosphere (right panel) recorded immediately after preparation and at increasing time intervals.

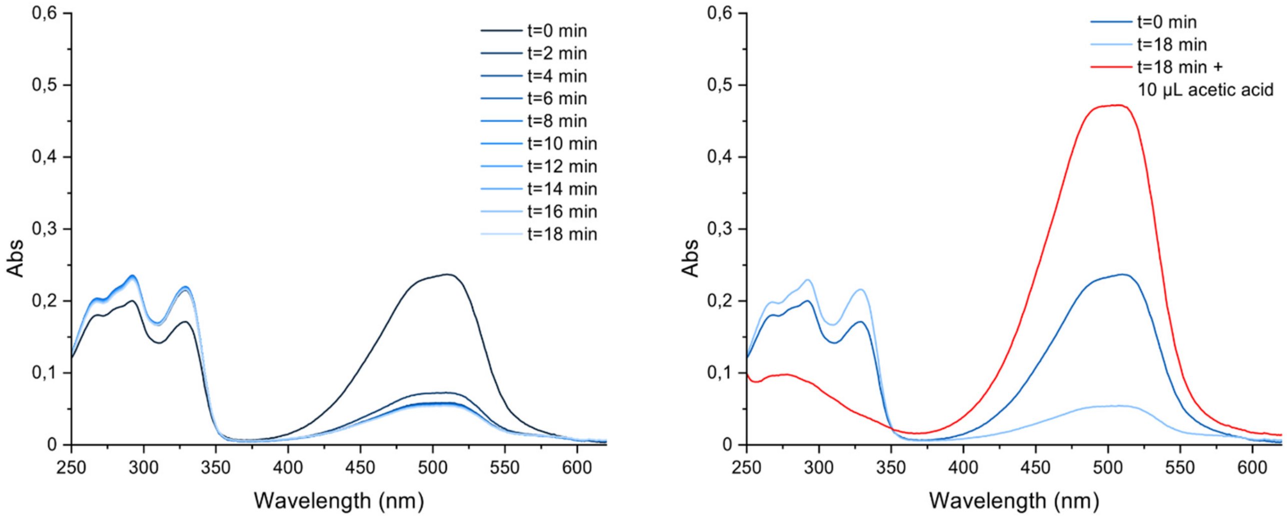

Figure S14. UV-vis spectra of compound **8** (10 µM) in H_2O:DMSO (90:10 v/v; MilliQ water, slightly acidic probably due to dissolved CO_2, pH ≈ 6) recorded immediately after preparation and at increasing time intervals (left panel). Notably, band II does not disappear completely, indicating that under the experimental conditions, the equilibrium is not fully shifted toward the hydrated form, and a fraction of the compound remains in its non-hydrated form. Right panel: UV-vis spectrum of the same solution before and after the addition of acetic acid.

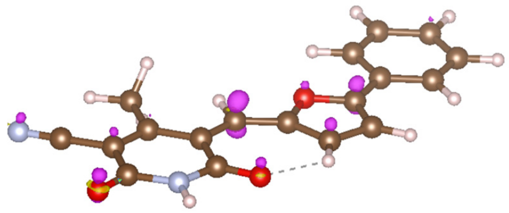

Figure S15. The Fukui function $f_+(\mathbf{r}) = n_{anion}(\mathbf{r}) - n_{neutral}(\mathbf{r})$ computed as the difference between the electron density of the anion of **8** and its neutral form (both evaluated at the optimized structure of the neutral form, from DFT B3LYP/6-311++G(d,p) calculations). The isosurface reported in magenta corresponds to the value of 0.01 atomic units of electron density.

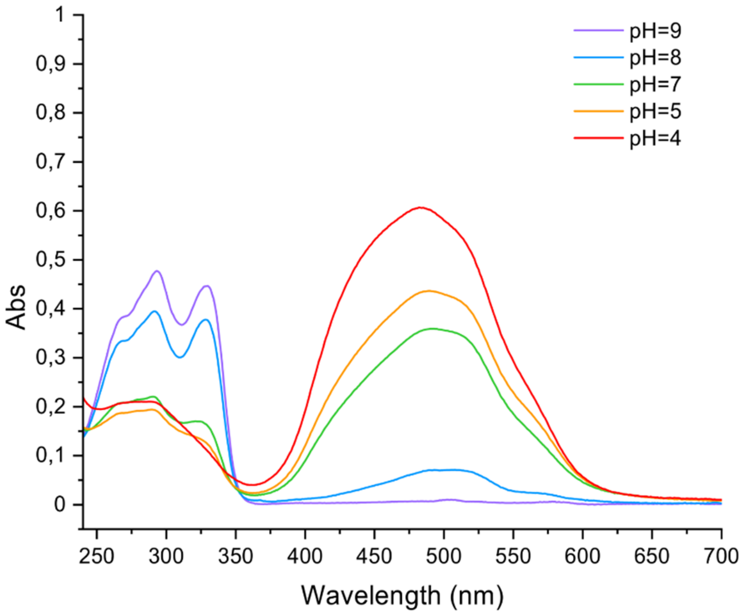

Figure S16. UV-vis spectra of compound **8** (6.9 µM final concentration) recorded immediately after addition to aqueous buffer solutions at different pH values (4-9). Each spectrum was obtained by adding 3 µl of a 6.9 mM solution of **8** in DMSO to 1 ml of buffer. The progressive increase in intensity of band II (450-550 nm) and the concurrent decrease of band I (250-350 nm) reflect the pH-dependent structural modification of the compound. It is worth noting that band II (400–550 nm) appears distinctly non-symmetrical, which may reflect multiple contributing factors. The presence of shoulders in a band that would be expected to display a bell-shaped profile, as previously observed, could arise from interactions between compound **8** and components of the buffer system, leading to micro-heterogeneous environments that affect the electronic transition. Additionally, given the limited aqueous solubility of compound **8**, partial precipitation or aggregation may occur under certain pH conditions, resulting in light scattering or baseline distortions that contribute to the observed asymmetry.

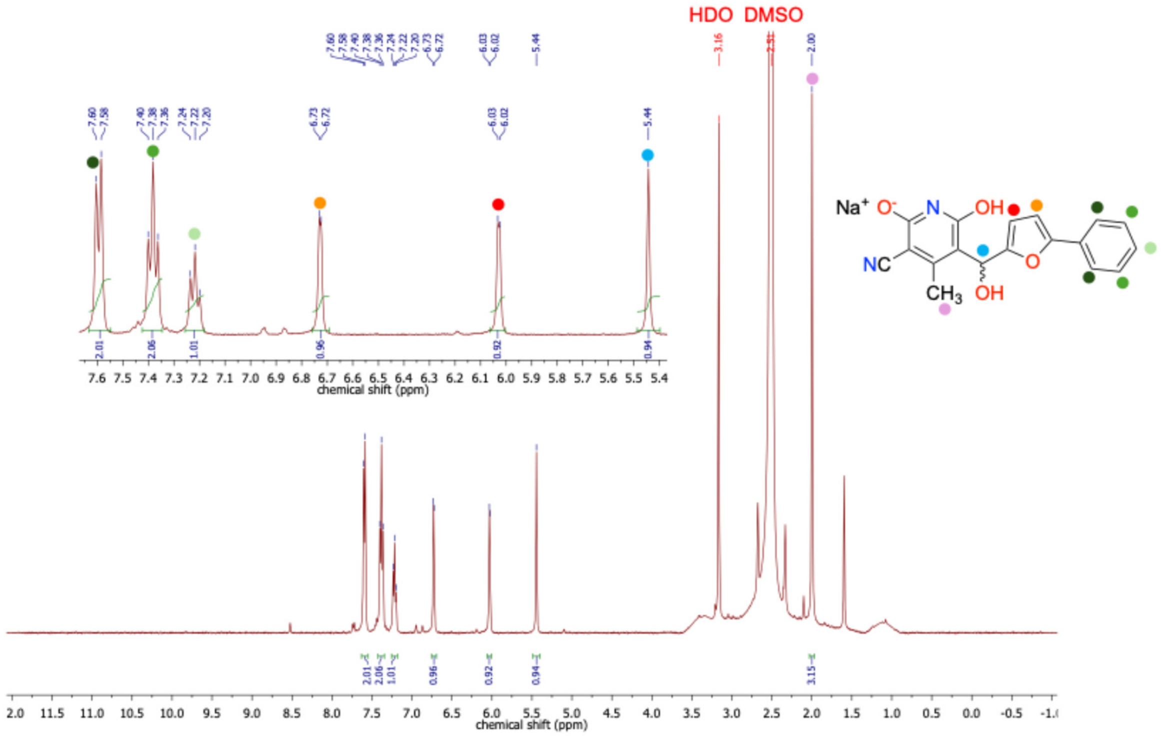

Figure S17. ^{1}H NMR (400 MHz, DMSO-d_6) of the hydrated adduct of compound **8** (2,6 dihydroxy, see Scheme 4 and Figure 6 in the manuscript). The spectrum reveals the expected singlet for the methyl protons at 2.0 ppm, a singlet at around 5.4 ppm for the methine proton and two resonances, at around 6.0 and 6.7 ppm respectively, for the two furan protons. The aromatic region displays two broad triplets, and one doublet centered at approximately 7.2 (1H), 7.4 (2H), and 7.6 (2H) ppm respectively, that account for the phenyl ring protons.

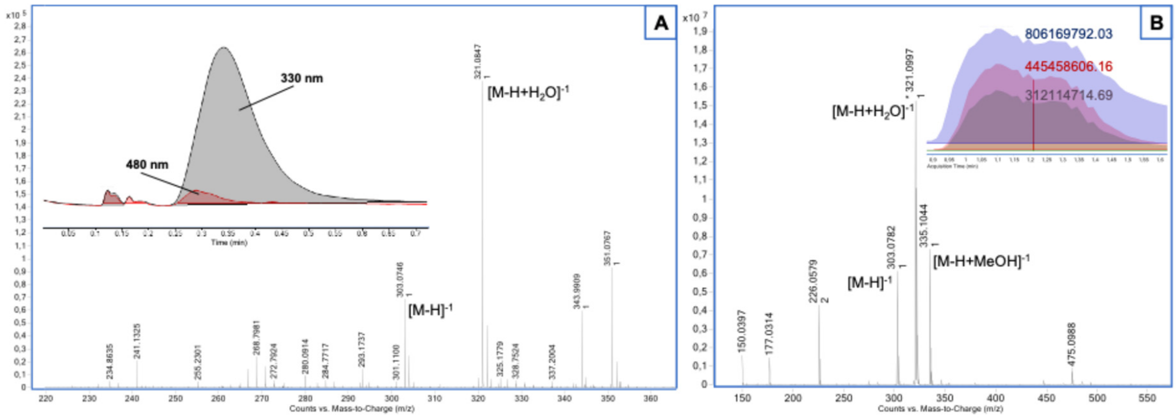

Figure S18. Fast flow injection (FFI) ESI-MS analysis of compound **8** in negative ion mode, coupled with UV-vis detection, using a pH 9 buffer as both solvent and eluent. The main peak observed at *m/z* 321.0847 corresponds to the water adduct ion [M-H + H_2O]$^-$ (calc. 321.0881). The inset (Panel A) shows UV-vis absorption at 330 and 480 nm, with a marked predominance of the band at 330 nm. Panel B shows the analysis of compound **8** previously treated under basic aqueous conditions, lyophilized and redissolved in methanol (used as both solvent ad eluent). The water adduct ion [M-H + H_2O]$^-$ is the major species, accompanied by a signal for the methanol adduct [M-H + MeOH]$^-$ at *m/z* 335.1044 (calc. 335.1037). The inset displays the extracted ion chromatograms for the three detected species, from bottom to top: [M-H]$^-$, [M-H + H_2O]$^-$ and [M-H + MeOH]$^-$.

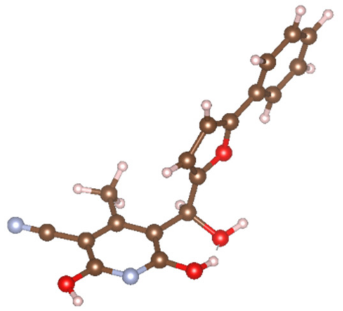

Figure S19. DFT-optimized structure of the 2,6-dihydroxy derivative. Calculations were performed on the isolated molecule at the B3LYP/6-311++(d,p) level, including D3BJ empirical dispersion.

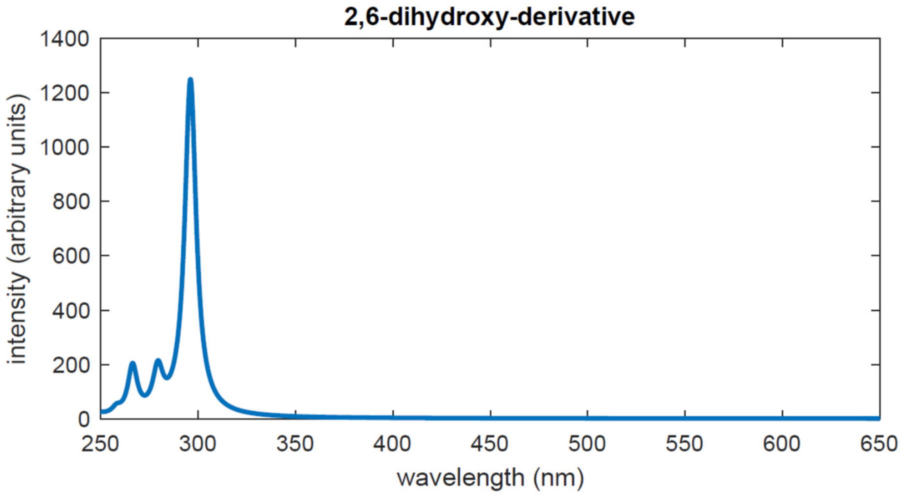

Figure S20. UV-vis TDDFT spectrum of the isolated hydration product of compound **8** (2,6-dihydroxy derivative).

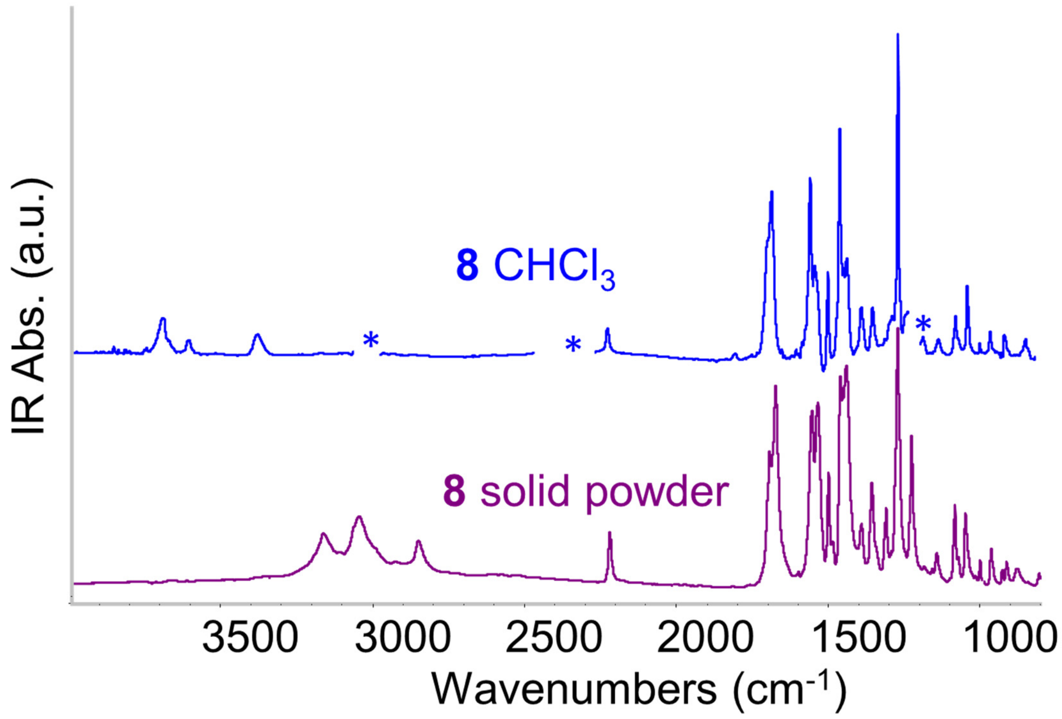

Figure S21. IR spectra of compound **8** in $CHCl_3$ and in the solid state (survey).

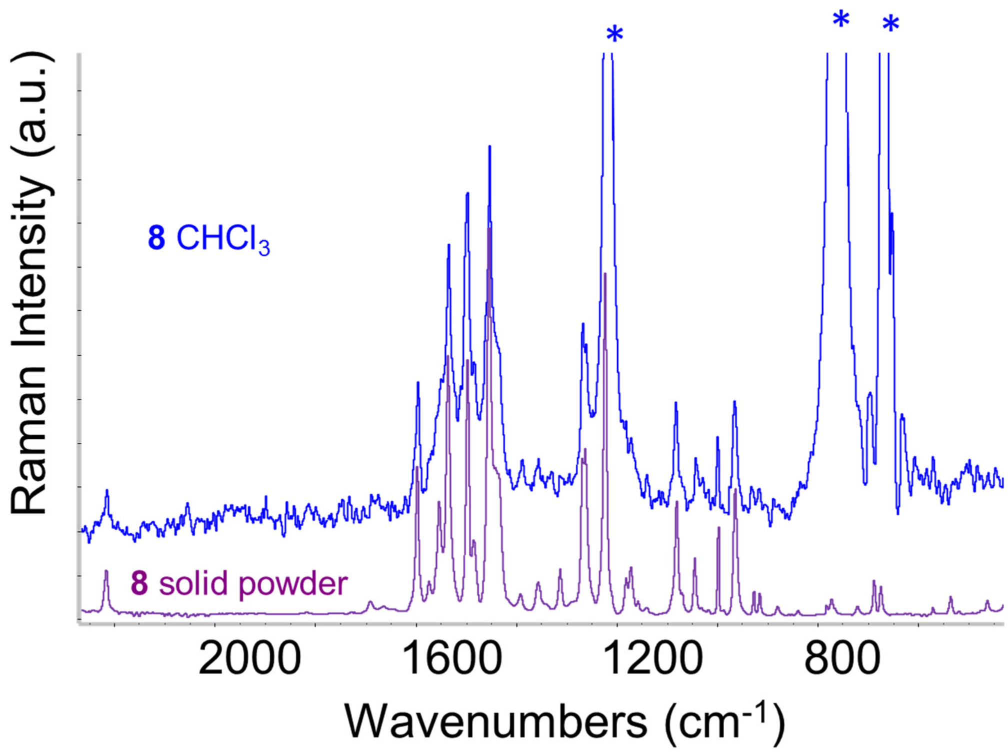

Figure S22. FT-Raman spectra of compound **8** in $CHCl_3$ and in the solid state (survey). Exciting laser line 1064 nm.

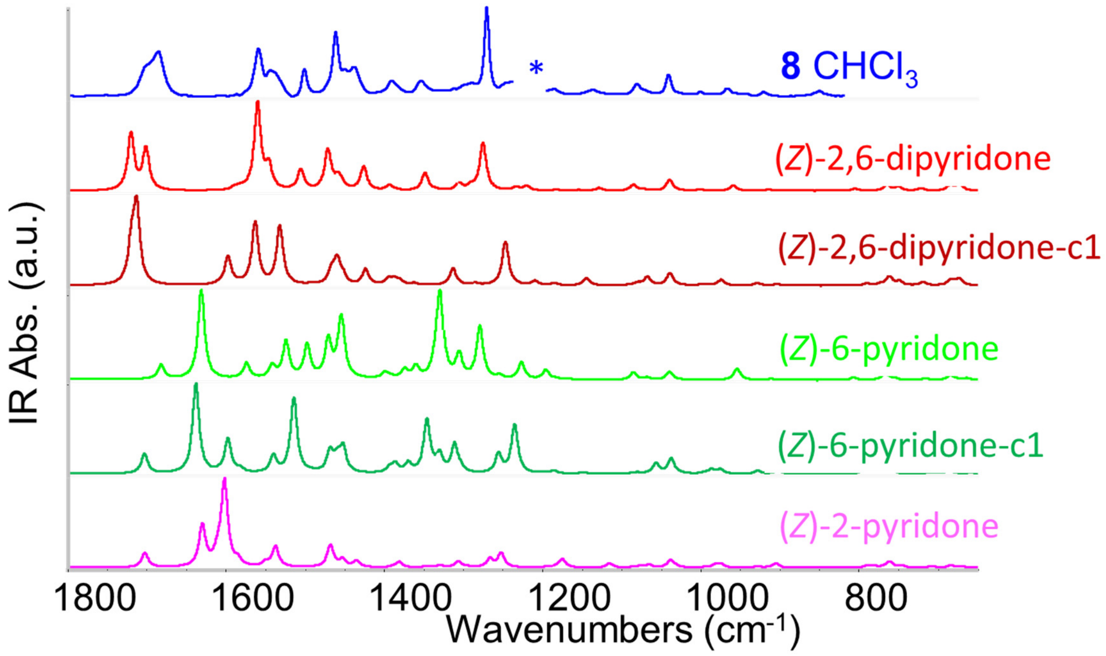

(a)

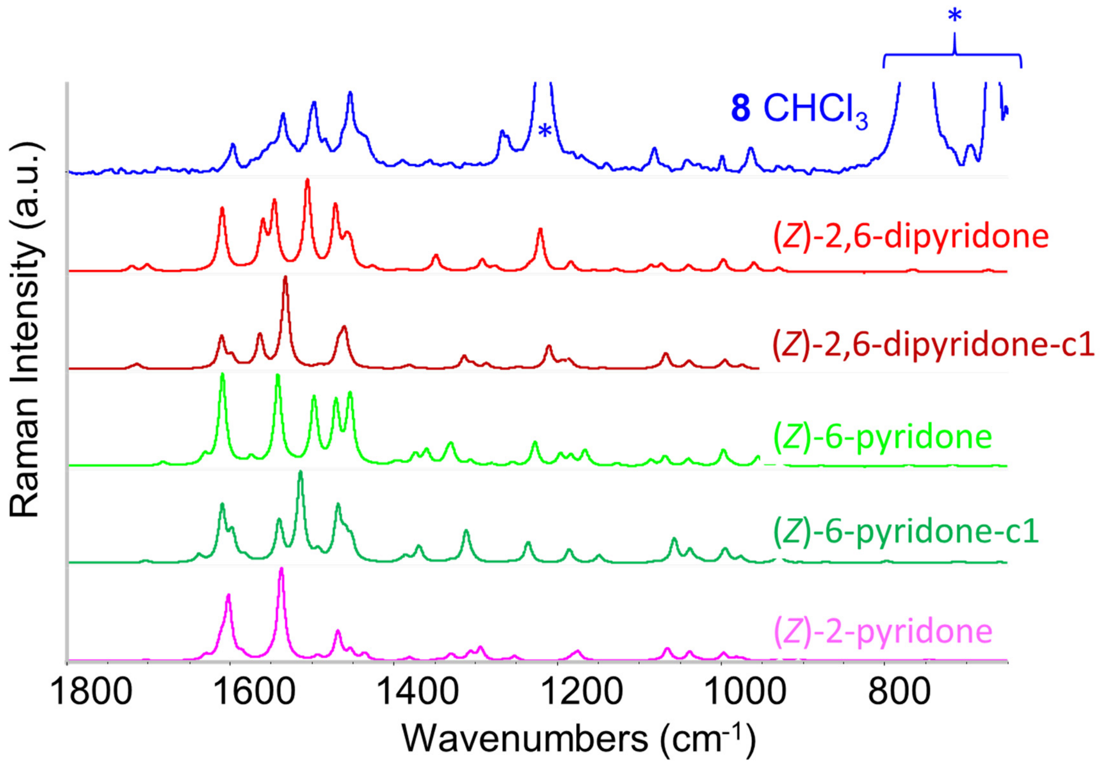

(b)

Figure S23. Comparison of the experimental IR – panel (a) – and Raman – panel (b) spectra of compound **8** in $CHCl_3$, with the DFT computed spectra for its different tautomers. The asterisks indicate bands of the solvent (removed in the IR spectrum).

View002

JEM Administrator 1/1

Title	:	IMG1
Instrument	:	JEM-F200(HRP)
Volt	:	200.00 kV
Mag.	:	x 300,000
Date	:	2025/03/03
Pixel	:	256 x 256

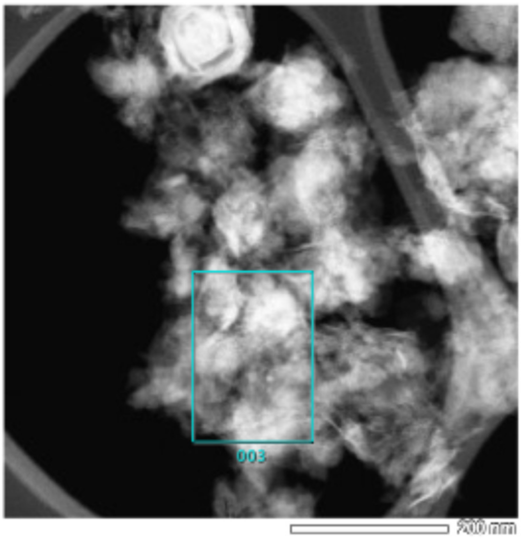

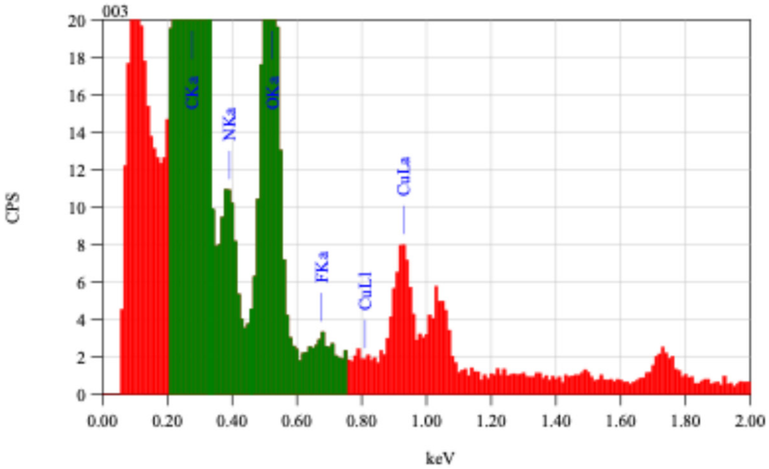

Acquisition Parameter

Instrument	:	JEM-F200(HRP)
Acc. Voltage	:	200.0 kV
Probe Current	:	7.47500 nA
PHA mode	:	T3
Real Time	:	52.37 sec
Live Time	:	50.00 sec
Dead Time	:	4 %
Counting Rate	:	4094 cps
Energy Range	:	0 - 40 keV

Thin Film Standardless Standardless Quantitative Analysis

Fitting Coefficient : 0.0983

Element	(keV)	Mass%	Counts	Sigma	Atom%	Compound	Mass%	Cation	K
C K (Ref.)	0.277	97.01	113022.36	0.29	97.75				1.0000
N K*		ND			ND				
O K !	0.525	2.90	6612.22	0.06	2.19				0.5110
F K*	0.677	0.09	292.67	0.01	0.06				0.3650
Cu K* (Excluded)									
Total		100.00			100.00				

JED-2300 AnalysisStation JEOL

Figure S24. STEM-EDX elemental mapping of pristine B60. No significant fluorine signal is detected. The low-level signal (0.06 atom %) is attributed to background or instrumental contamination.

View001

JEM Administrator 1/1

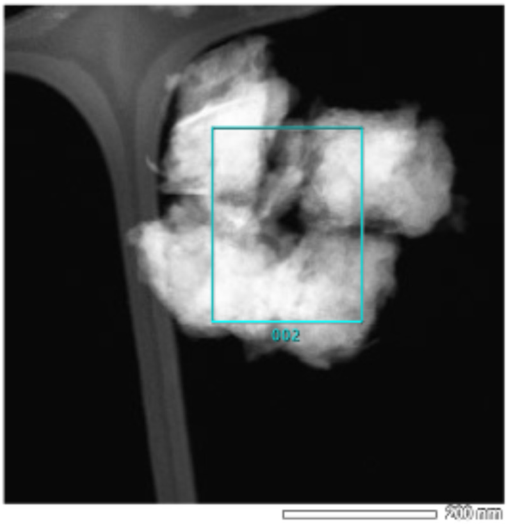


```
Title          : IMG1
----------------------------
Instrument     : JEM-F200(HRP)
Volt           : 200.00 kV
Mag.           : x 300,000
Date           : 2025/03/03
Pixel          : 256 x 256
```

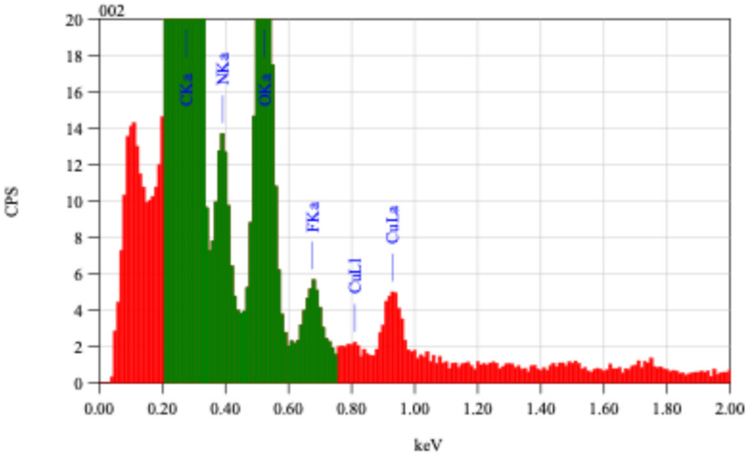


```
Acquisition Parameter
Instrument     : JEM-F200(HRP)
Acc. Voltage : 200.0 kV
Probe Current: 7.47500 nA
PHA mode       : T4
Real Time      : 57.23 sec
Live Time      : 50.00 sec
Dead Time      : 12 %
Counting Rate: 4358 cps
Energy Range :  0 - 40 keV
```

Thin Film Standardless Standardless Quantitative Analysis
Fitting Coefficient : 0.0708

Element	(keV)	Mass%	Counts	Sigma	Atom%	Compound	Mass%	Cation	K
C K (Ref.)	0.277	96.27	109347.93	0.29	97.16				1.0000
N K !	0.392	0.46	766.81	0.02	0.39				0.6760
O K !	0.525	2.99	6645.25	0.06	2.27				0.5110
F K	0.677	0.29	896.64	0.02	0.18				0.3650
Cu K* (Excluded)									
Total		100.00			100.00				

JED-2300 AnalysisStation JEOL

Figure S25. STEM-EDX elemental mapping of **8-F**@B60. A detectable fluorine signal (0.18 atom %) confirms loading of the fluorinated compound onto the graphene nanoparticle surface.

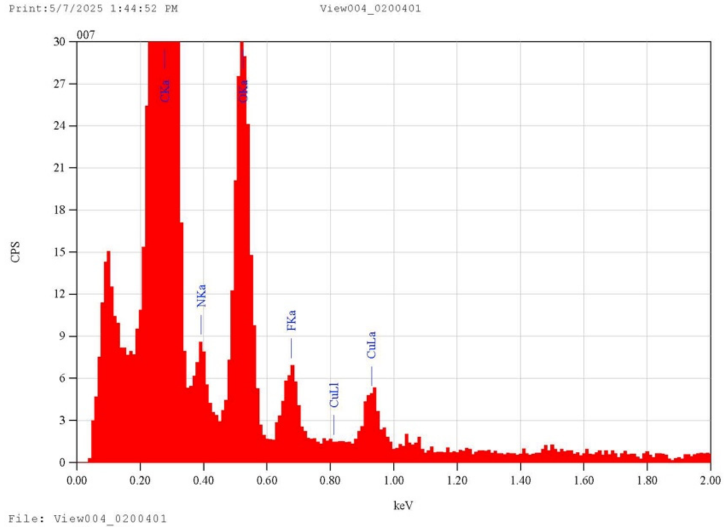

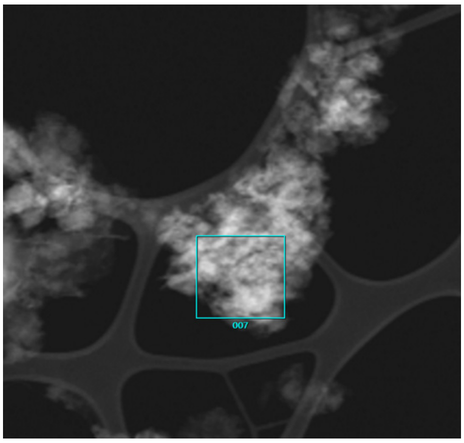


```
File: View004_0200401
Specimen ID: 007
Date: 5/7/2025 1:43:19 PM

Acquisition Parameter
Acc. Voltage    :      200.0 kV          Probe Current   :     7.47500 nA
Real Time       :      30.18 sec         Dead Time       :       10 %
Live Time       :      26.74 sec         Counting Rate   :      3321 Counts/sec
Preset          : Real Time 10000000 sec
Energy Range    :  0 - 40 keV            PHA Mode        : T4
```

Thin Film Standardless Standardless Quantitative Analysis
Fitting Coefficient : 0.0765

Element	(keV)	Mass%	Counts	Sigma	Atom%	Compound	Mass%	Cation	K
C K (Ref.)	0.277	95.86	45112.18	0.45	96.90				1.0000
N K !	0.392	0.07	48.43	0.02	0.06				0.6760
O K !	0.525	3.60	3311.89	0.10	2.73				0.5110
F K	0.677	0.48	618.02	0.04	0.31				0.3650
Cu K* (Excluded)									
Total		100.00			100.00				

Figure S26. STEM-EDX elemental mapping of **8-CF3**@B60. The analysis reveals an increased fluorine load (0.31 atom %), consistent with the higher fluorine content of the trifluoromethylated derivative onto the graphene nanoparticle surface.

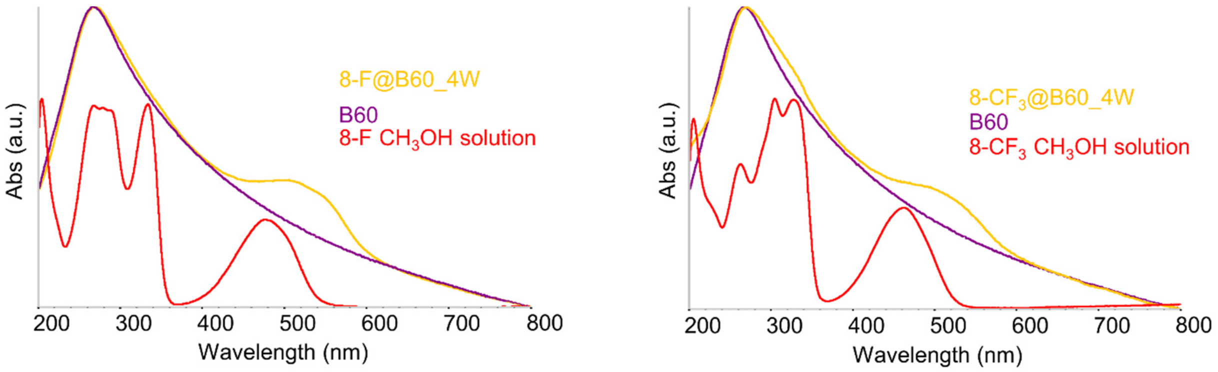

(a) **(b)**

Figure S27. UV-vis spectra of aqueous dispersions of B60 conjugates, compared with the spectrum of bare B60 in water (violet line) and of the free molecules in CH_3OH (red line). **(a)** spectrum of **8-F**@B60 after four washing steps; **(b)** spectrum of **8-CF3**@B60 after four washing steps.

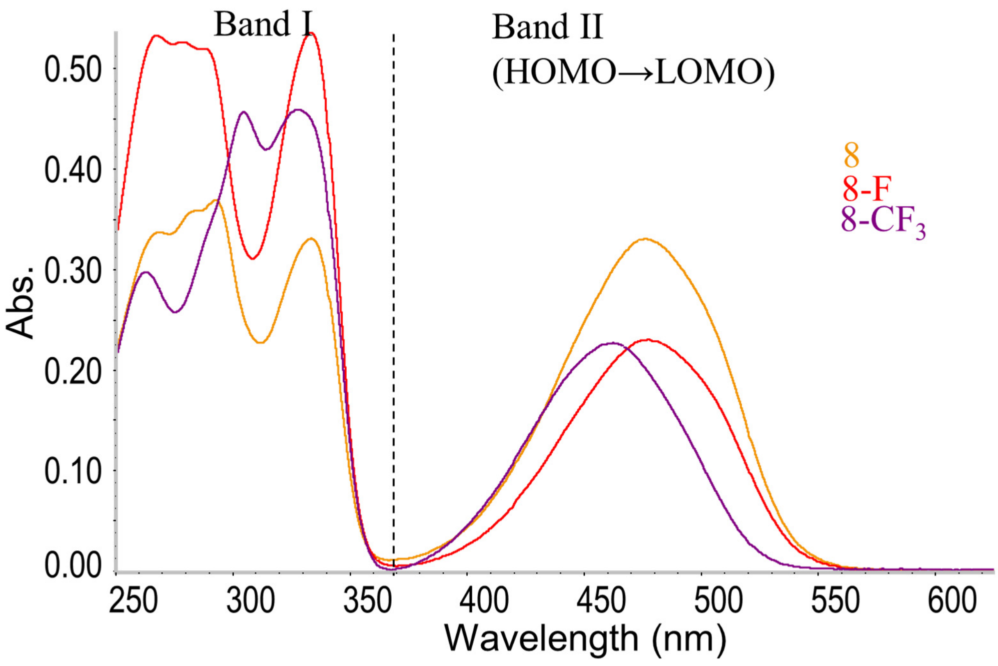

Figure S28. UV-vis spectra of compounds **8**, **8-F** and **8-CF$_3$** in CH_3OH.

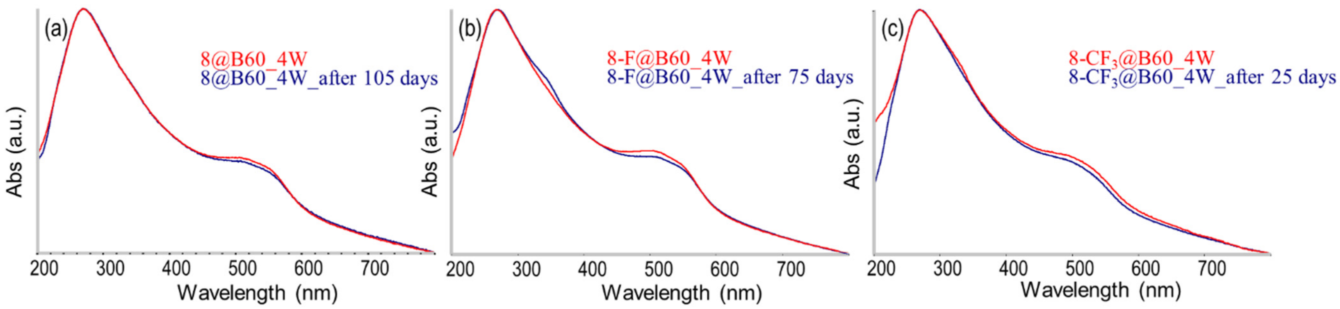

Figure S29. UV-vis spectra of **8**@B60, **8-F**@B60, and **8-CF$_3$**@B60 water dispersions: evolution over time.

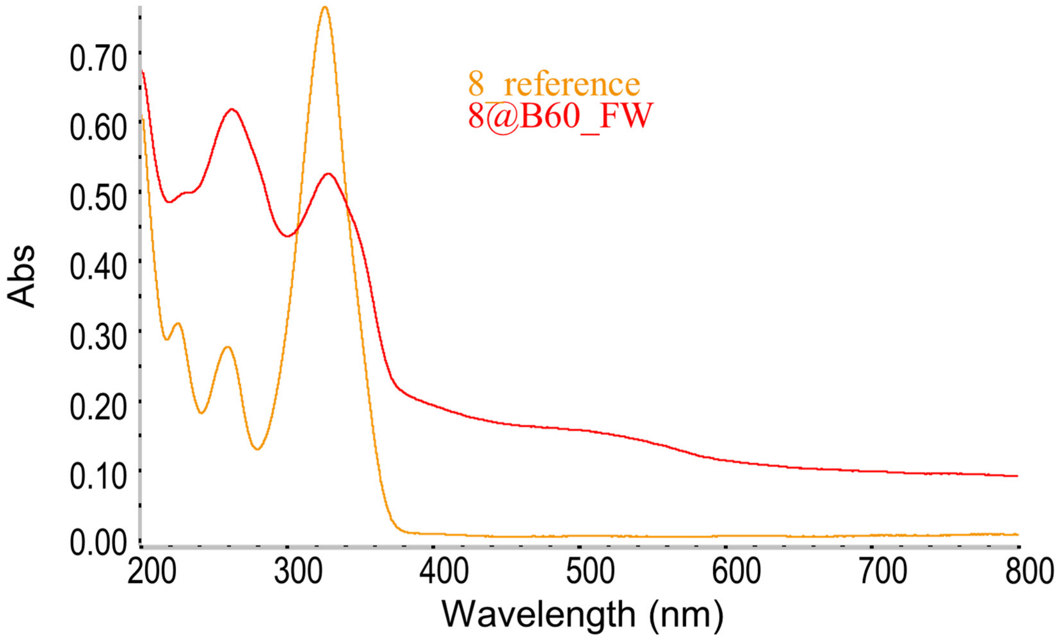

Figure S30. UV-vis spectrum of the supernatant (FW) after centrifugation of the **8**@B60 dispersion, before the washing steps. Comparison with the spectrum of the CH_3OH/H_2O solution of compound **8**, subjected to the same procedure.

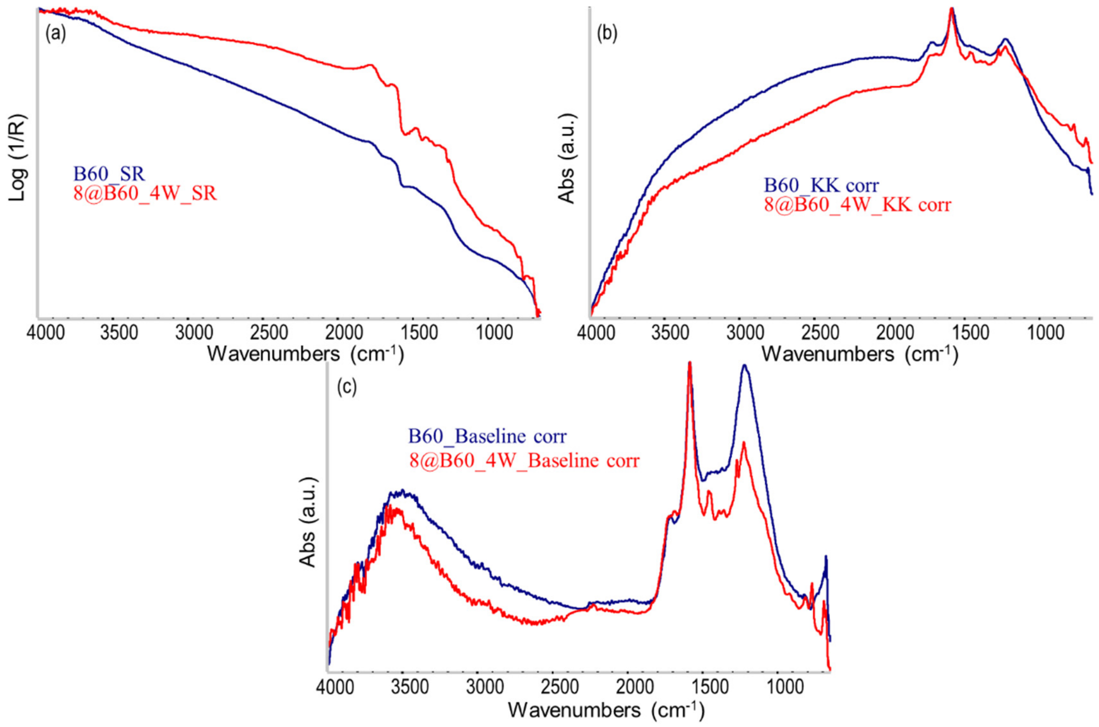

Figure S31. IR spectrum of B60 and **8**@B60: **(a)** Specular Reflection (SR) spectrum of the powder; **(b)** the absorption spectra obtained after Kramers-Kronig transformation of the SR spectra displayed in panel (a); **(c)** IR absorption spectra after baseline correction of the spectra displayed in (b).

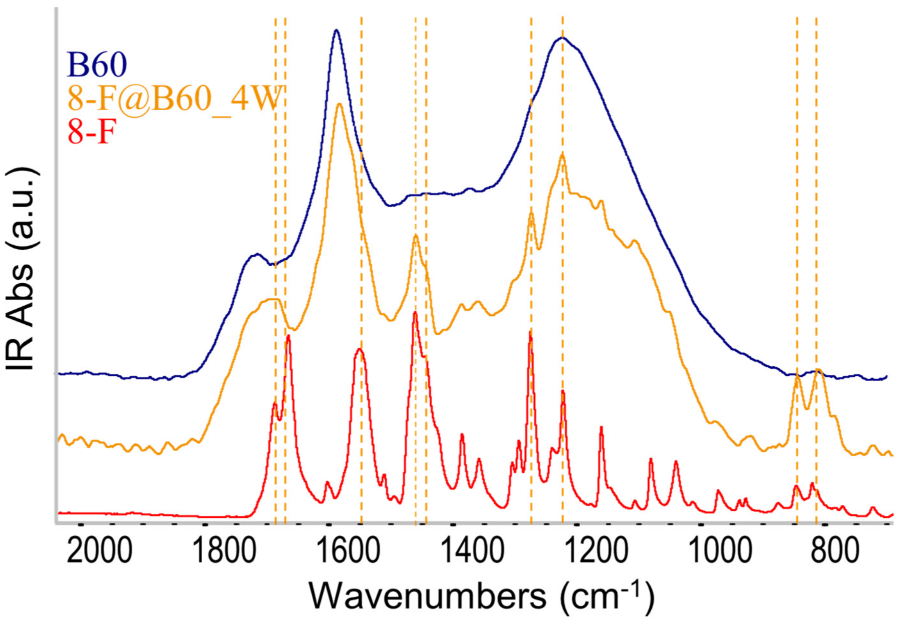

(a)

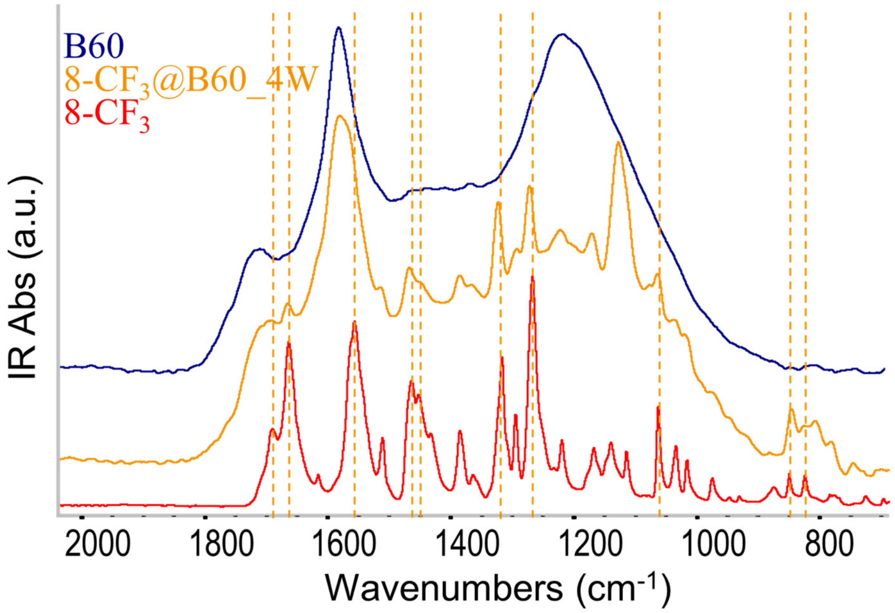

(b)

Figure S32. IR spectra of **8-F**@B60 **(a)** and **8-CF$_3$**@B60 **(b)**: comparison with the IR spectra of **8-F**, **8-CF$_3$** and of bare B60.

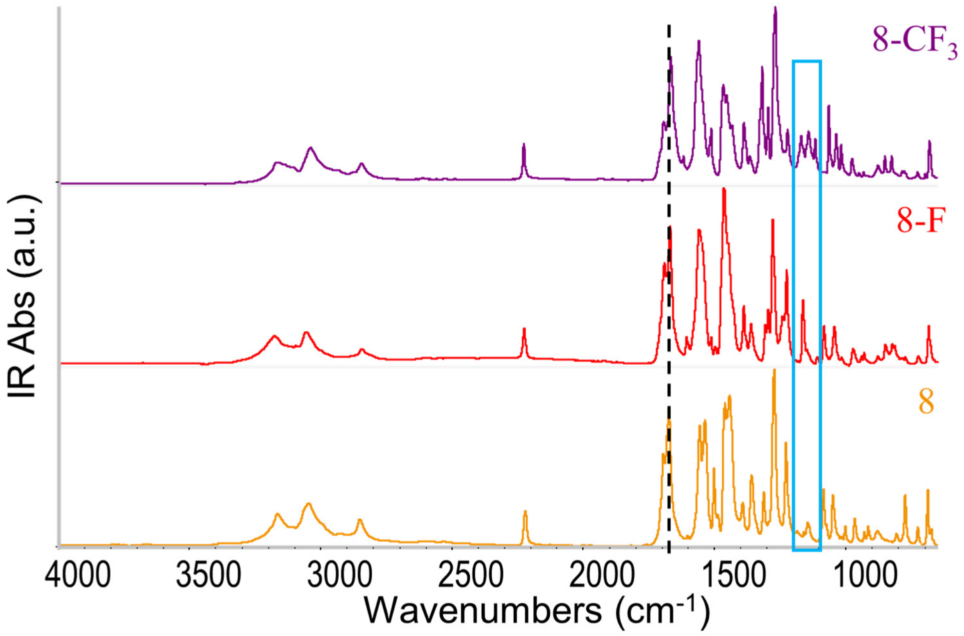

Figure S33. Infrared spectra of **8**, **8-F** and **8-CF_3**. Solid-state samples.

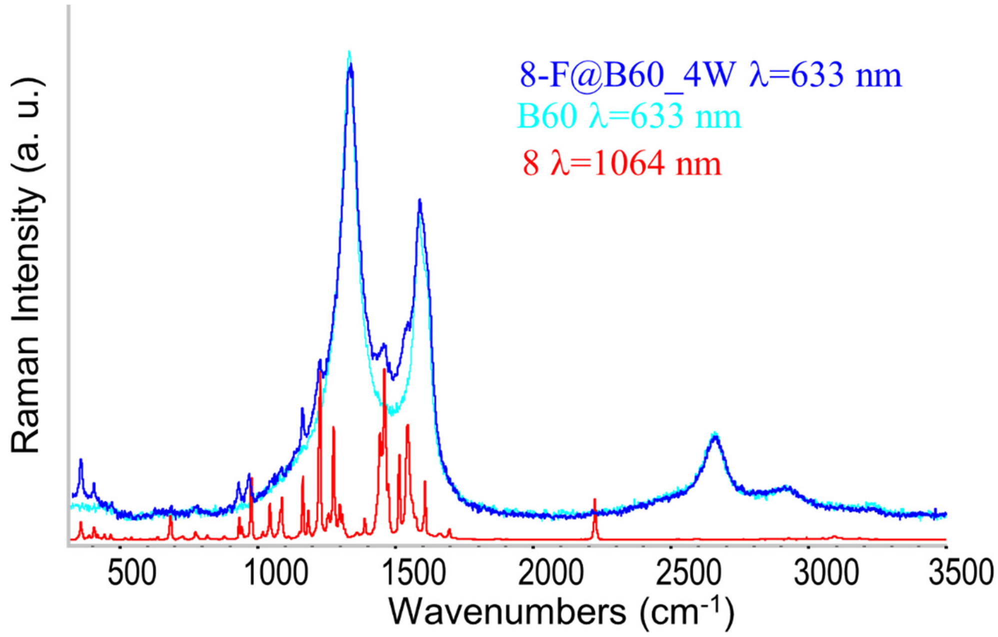

(a)

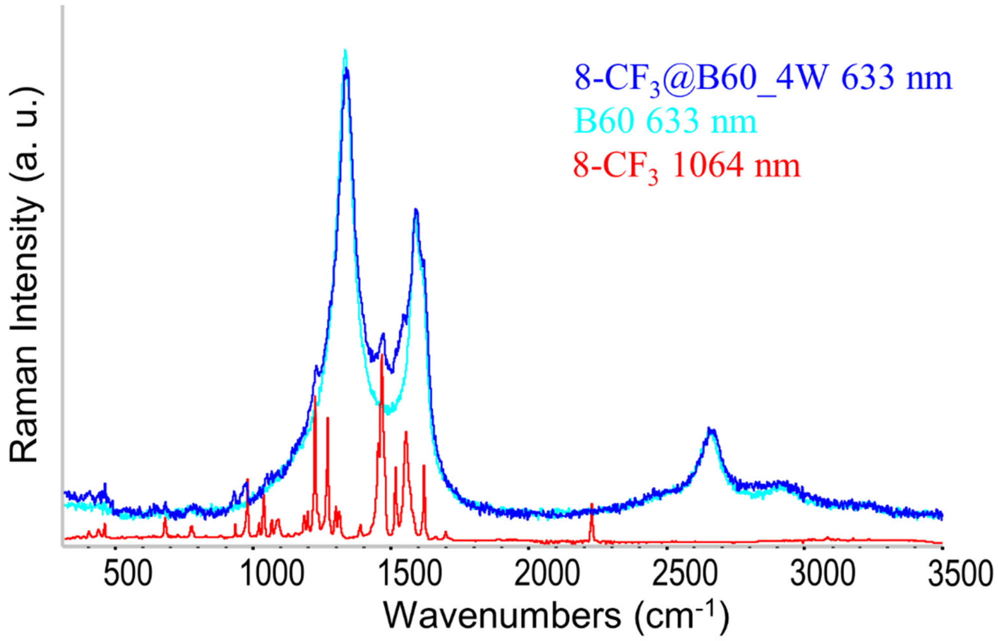

(b)

Figure S34. Raman spectra of **8-F**@B60 **(a)** and **8-CF$_3$**@B60 **(b)**: comparison with the Raman spectra of compounds **8-F** and **8-CF$_3$** and with the Raman spectrum of bare B60.

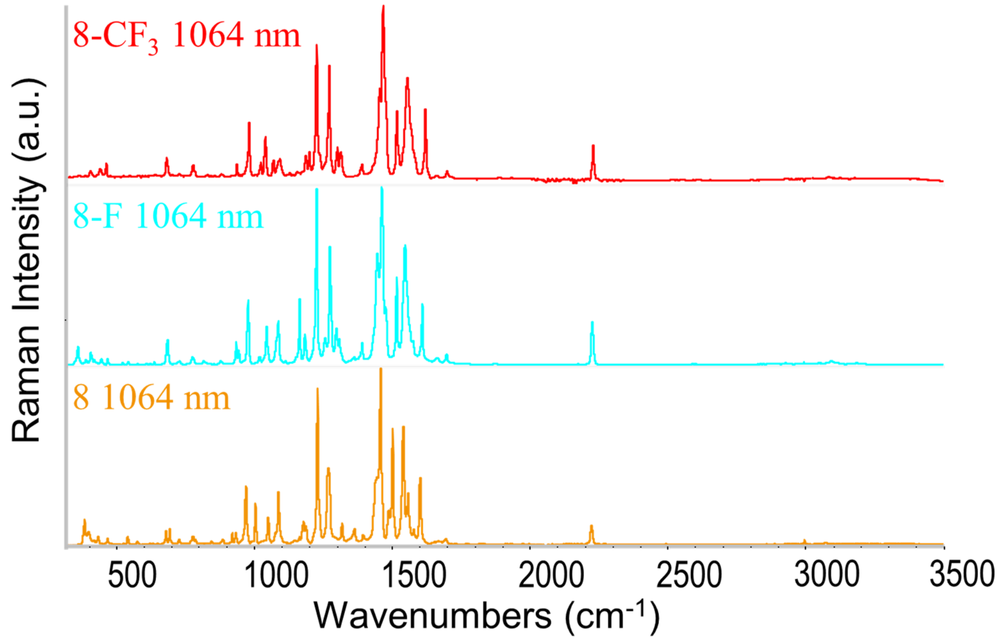

Figure S35. FT-Raman spectra (λ_{exc}= 1064 nm) of **8**, **8-F** and **8-CF$_3$**. Solid-state samples.

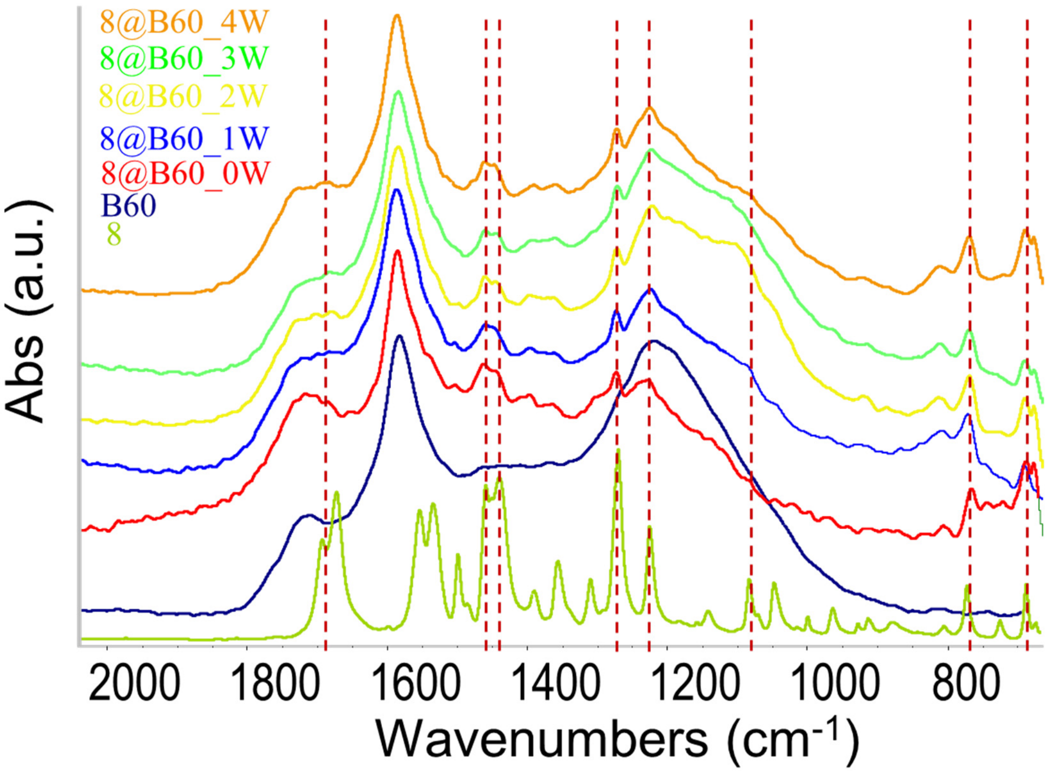

Figure S36. IR spectra of **8**@B60 after multiple washing cycles. For comparison, the spectra of bare B60 and free compound **8** are shown at the bottom. All spectra are vertically offset for clarity.

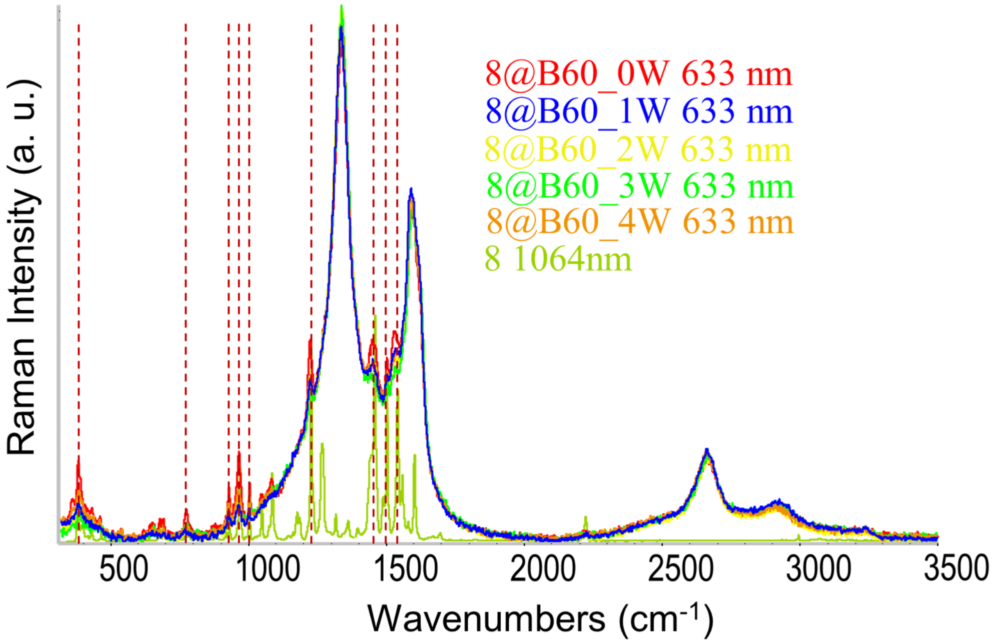

Figure S37. Raman spectra of **8**@B60 after multiple washing cycles. The spectra of bare B60 and free compound **8** are shown for comparison.

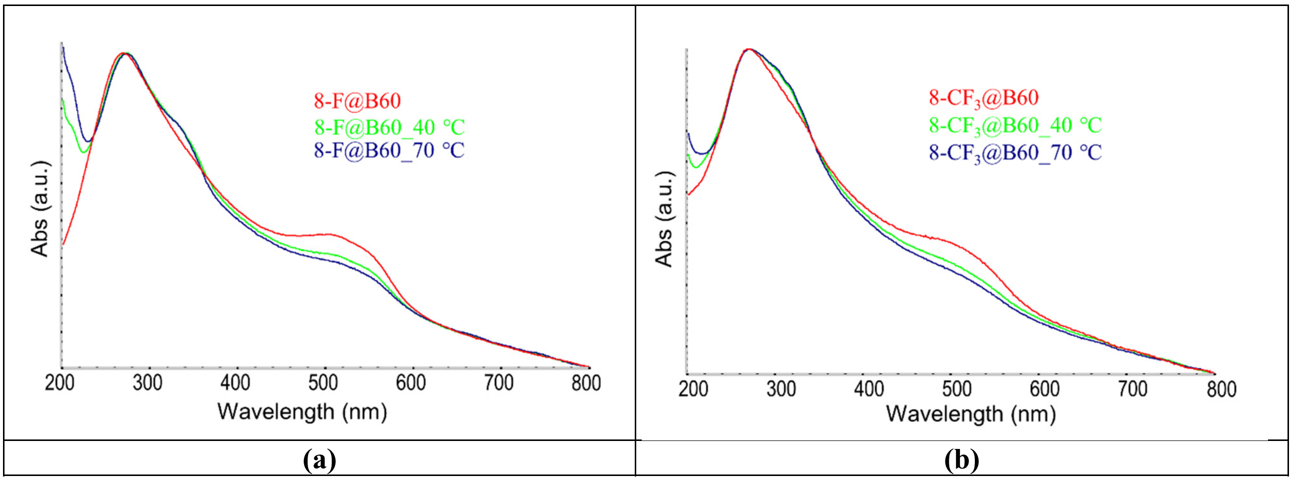

Figure S38. Drug release of: **8-F**@B60 (panel a) and **8-CF$_3$**@B60 (panel b). UV-vis spectra of aqueous dispersions of the conjugates recorded at ambient temperature (red line) and after heating at 40 °C (40 minutes) and at 70 °C (20 minutes).

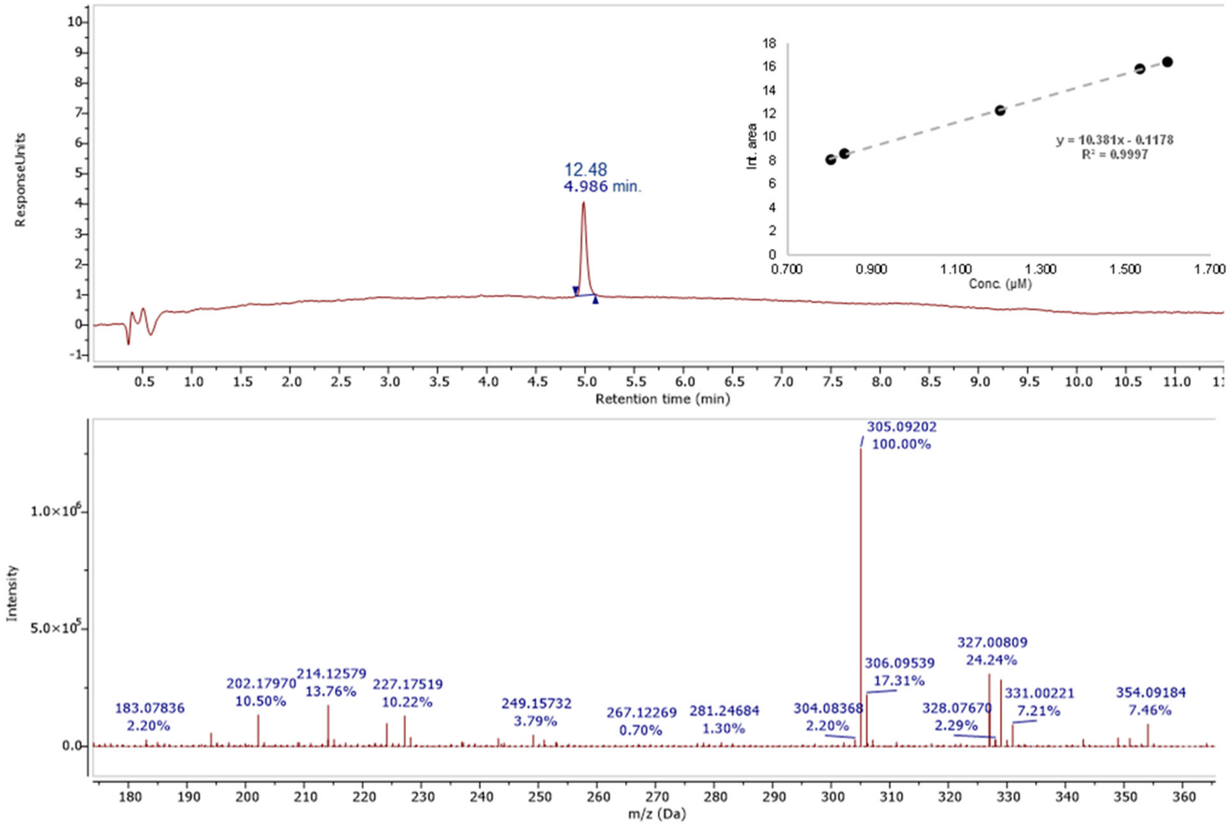

Figure S39. UPLC-HRMS (ESI+) analysis of the supernatant after heating an aqueous dispersion of **8**@B60 at 70 °C for 1 hour followed by centrifugation. Top: UPLC chromatogram recorded at 480 nm with a calibration curve specifically created for **8** reported in the insert; bottom: HRMS spectrum showing the base peak corresponding to **8** released from B60, $[M+H]^+$ found = 305.09202 *m/z* [M+H]+, calc. = 305.0907 *m/z*.

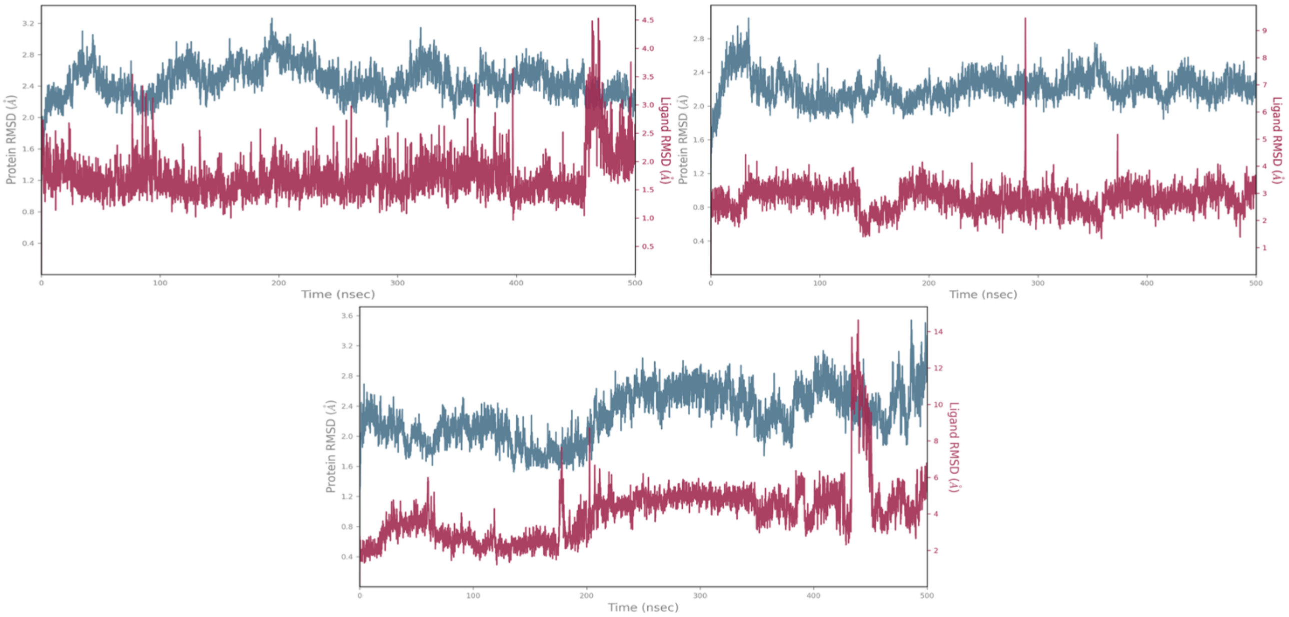

(Z)-6-pyridone

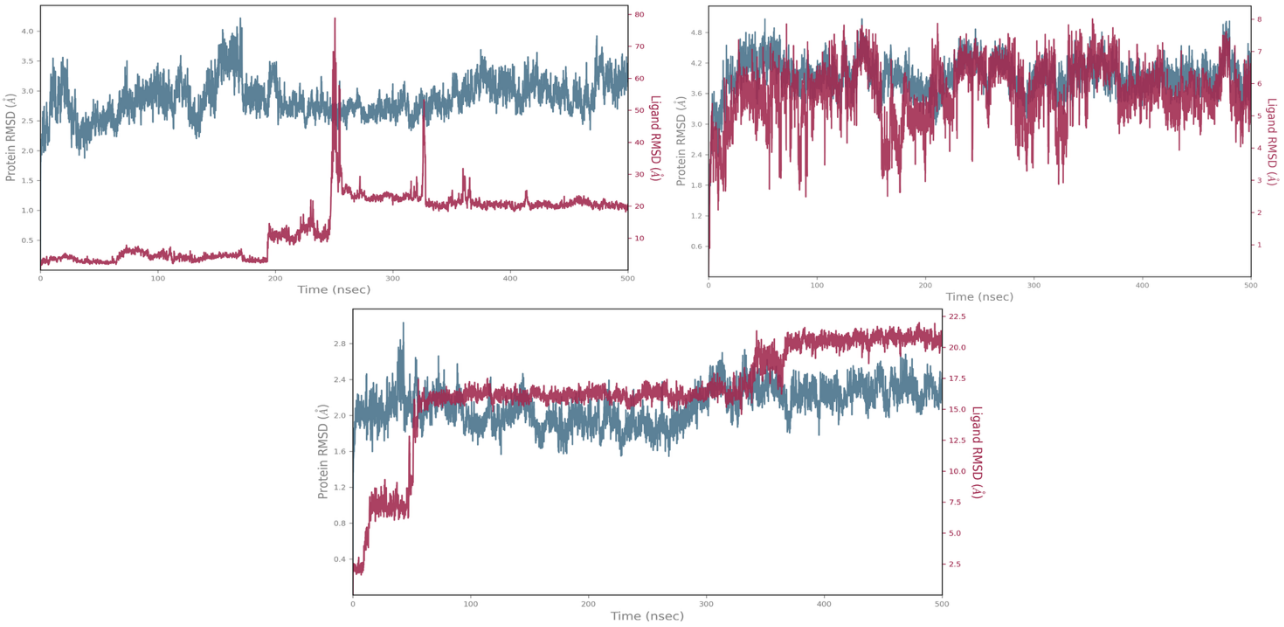

(Z)-2-pyridone

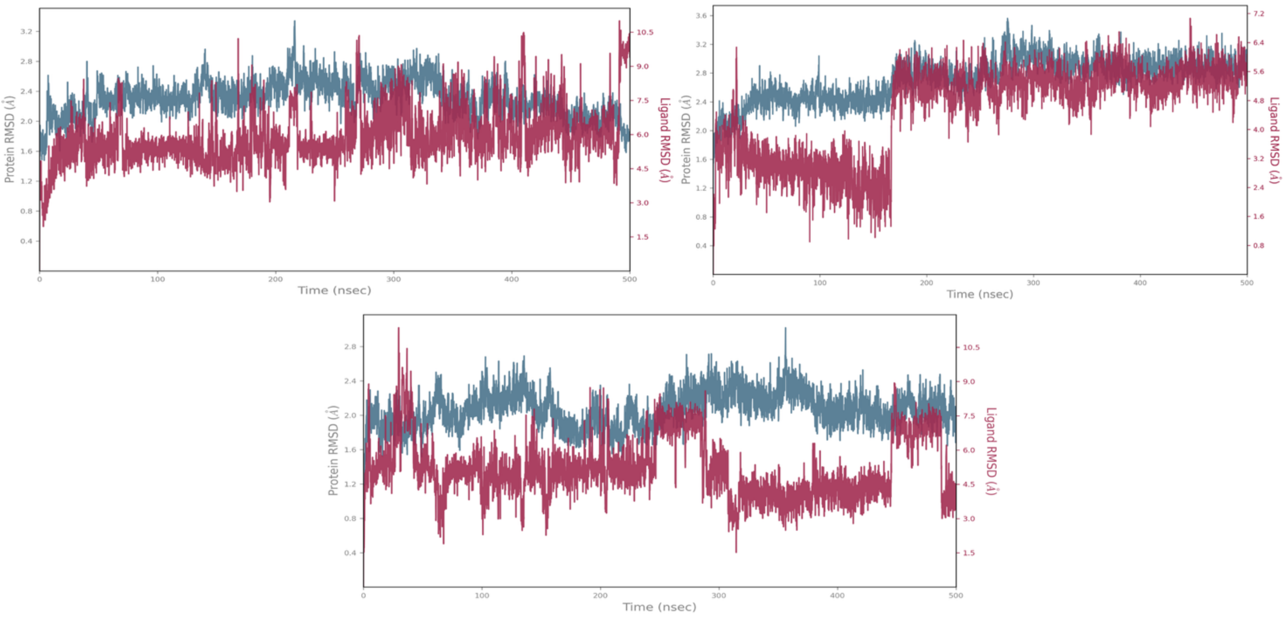

(Z)-2,6-dipyridone

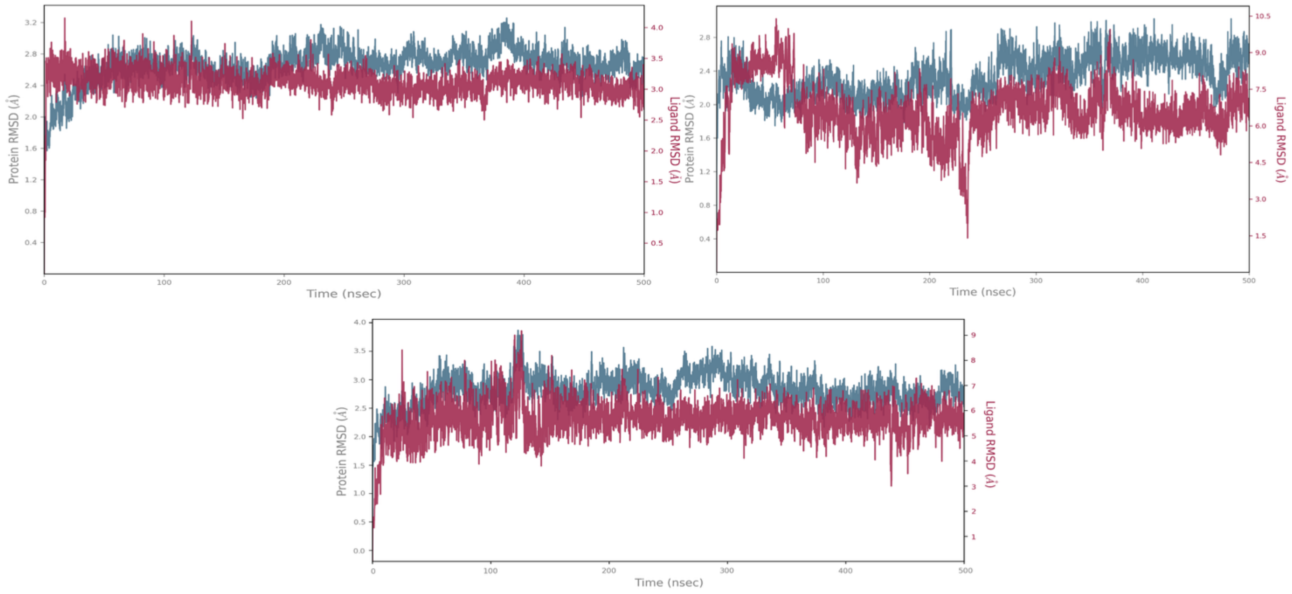

2,6-dihydroxy derivative

Scheme S1. Time evolution of the Cα-RMSD (blue) and $RMSD_{lig}$ (red) of different (*Z*)-tautomers of compound **8** and of the 2,6-dihydroxy water adduct.

Table S1. Wavenumbers of the IR transitions of **8**: experimental data from a $CHCl_3$ solution, solid-state sample, and of the **8**@B60 conjugate. Calculated wavenumbers are reported from DFT modelling of the (*Z*)-2,6-dipyridone monomer and its hydrogen-bonded dimer ((*Z*)-2,6-dipyridone-d_2). DFT wavenumbers are scaled by a factor of 0.98. Both absolute and relative IR intensities are included; only transitions with relative intensity ($I_{IR,rel} \geq 0.1$) compared to the most intense band in the fingerprint region (1800 – 600 cm^{-1}) are shown. Weaker transitions are reported when the corresponding bands are clearly observed in the experimental spectrum. Bold characters highlight very strong absorption intensities, while the grey background highlights major changes between the isolated molecule (in solution) and the hydrogen-bonded dimer (in the solid state).

(*Z*)-2,6-dipyridone	**8** (solution $CHCl_3$)	**8**@B60 (solid)	(*Z*)-2,6-dipyridone		(*Z*)-2,6-dipyridone-d2	**8** (crystal)	(*Z*)-2,6-dipyridone - d2	
DFT	exp	exp	DFT		DFT	exp	DFT	
ν (cm^{-1}) (scaled 0.98)	ν (cm^{-1})	ν (cm^{-1})	I_{IR} (km mol^{-1})	$I_{IR.rel}$	ν (cm^{-1}) (scaled 0.98)	ν (cm^{-1})	I_{IR} (km mol^{-1})	$I_{IR.rel}$
685		690	37	0.04	688	686	119	0.08
751		719	48	0.05	727	724	88	0.05
765		767	58	0.06	767	771	95	0.05
806		807	26	0.03	813	803	44	0.03
					907	877	104	0.08
915	929		6	0.01	915	927	35	0.03
960	967		63	0.06	955	963	118	0.08
1040	1041		120	0.12	1045	1047	149	0.11
1085	1083		32	0.03	1085	1084	58	0.03
1221	solvent	1223	51	0.05	1226	1224	348	0.24
**1276**	**1271**	**1272**	**537**	**0.55**	**1278**	**1269**	**934**	**0.62**
1292	broad		45	0.05	1306	1310	312	0.22
1306	broad		75	0.08	1310		95	0.05
1349	1354		195	0.20	1352	1357	444	0.30
1395	1392		46	0.02	1395	1391	52	0.03
1426	1441	1445	265	0.27	**1434**	**1440**	**1034**	**0.68**
1453			47	0.05	1454		112	0.08
1459	1450		123	0.13	1456	**1459**	231	0.16
					1467		293	0.19
**1472**	**1464**	**1463**	**442**	**0.46**	**1471**		**680**	**0.46**
1506	1503	1503	225	0.23	1503	1499	410	0.27
1546	1546 (+1540)	1540	256	0.26	**1542**	**1535**	**1199**	**0.78**
**1560**	**1560**	**1556**	**972**	**1.00**	**1554**	**1554**	**1506**	**1.00**
**1702**	**1686**	1695-1650	**460**	**0.47**	**1685**	**1669**	**1000**	**0.68**
**1721**	**1704** (+ sh?)		**636**	**0.65**	**1710**	**1693**	**996**	**0.68**
2283	2228		75	0.08	2281	2219	155	0.11

3512	3379		81	0.08	3210	**3164-3045-2849**	**4063**	**2.70**

Table S2. Raman transitions wavenumbers of compound **8**: experimental data are shown for $CHCl_3$ solution, solid-state sample, and the **8**@B60 conjugate. These are compared with DFT-calculated wavenumbers for the (*Z*)-2,6-dipyridone monomer and its hydrogen-bonded dimer. DFT wavenumbers are scaled by a factor of 0.98. Both absolute and relative Raman activities are reported; transitions with relative intensity ($I_{Raman,rel} \geq 0.1$) compared to the most intense band are included. Weaker transitions are also listed when the corresponding band is clearly observed in the experimental spectrum. Bold values indicate very strong absorption intensities.

(*Z*)-2,6-dipyridone	**8** (solution $CHCl_3$)	**8**@B60 (solid)	(*Z*)-2,6-dipyridone		(*Z*)-2,6-dipyridone - d2	**8** (crystal)	(*Z*)-2,6-dipyridone – d2	
DFT	exp	exp	DFT		DFT	exp	DFT	
ν (cm^{-1}) (scaled 0.98)	ν (cm^{-1})	ν (cm^{-1})	I_{Raman} (Å^4 mol^{-1})	$I_{Raman,rel}$	ν (cm^{-1}) (scaled 0.98)	ν (cm^{-1})	I_{Raman} (Å^4 mol^{-1})	$I_{Raman,rel}$
673		685	77	0.01	676	688	83	0.01
765		771	106	0.02	773	771. 782	146	0.01
929	931	924	196	0.03	929	927	403	0.03
960	965	958	433	0.07	954	964	810	0.06
997	999	998	614	0.09	997	998	1390	0.10
1040	1043	1041	335	0.05	1044	1044	672	0.05
1073	1082	1078	402	0.06	1074	1081	650	0.05
1087			209	0.03	1088	1172	687	0.05
1184	1182		569	0.09	1183	1182	1207	0.09
**1221**	**solvent**	**1218**	**2544**	**0.38**	1226	1224	3272	0.24
1234			265	0.04	1251	1262	2592	0.19
1276	1269		341	0.05	1279	1267	246	0.02
1292			705	0.11	1313	1312	308	0.02
1349	1356		1062	0.16	1351	1357	2221	0.16
1391			254	0.02	1394	1391	91	0.01
1426	1434	1423	1476	0.04	1435	1434 +sh	1018	0.08
1453	1454	1469	1494	0.22	1453	1454	3346	0.25
1459				0.23	1457		2051	0.15
					**1465**		**5197**	**0.38**
1472	**1484**		**4473**	**0.68**	1473	1483	4391	0.32
**1506**	**1499**	**1496**	**6625**	**1.00**	**1504**	**1496**	**10764**	**0.79**
**1546**	**1536**	**1523**	**4966**	**0.75**	**1543**	**1535**	**13557**	**1.00**
**1560**	**1551-1576**		**3368**	**0.51**	1557	1553-1573	3572	0.26
**1610**	**1597**		**4952**	**0.75**	**1610**	**1596**	**12239**	**0.90**
**1702**	1689		586	0.09	1672	1690	799	0.06
1721			427	0.06	1714		866	0.06
2283	**2216**		**2410**	**0.36**	**2281**	**2217**	**3808**	**0.28**

Comments on the DFT-predicted vibrational spectra of the Z tautomers of compound 8

The computed IR spectra (Figure S3 and S20) of the (*Z*)-dipyridone and (*Z)*-pyridone species reveal the following key differences:

- The presence of an O–H stretching band in the (*Z*)-pyridone species and N–H stretching bands in the (*Z*)-dipyridone species is evident. These bands are clearly distinguishable due to their well-separated frequencies; an expected result based on standard empirical spectroscopic correlations.

- The stretching band of the CN triple bond occurs at lower wavenumbers in the case of the (*Z*)-pyridone tautomers compared to the (*Z*)-dipyridone forms.

- Two normal modes, both involving C=O stretching coupled with stretching of the adjacent C–N bonds, are characteristic of the (Z)-pyridone tautomers. This coupling leads to a marked red shift and a distinct intensity pattern, clearly different from the two separate C=O stretching transitions observed in the (*Z*)-dipyridone species. See vibrational eigenvectors in Scheme S2.

Several other marker bands corroborate the conclusion that the analysis of the IR spectra should allow to identify the tautomer/s in the sample. More subtle, but clear differences in the spectral pattern – both IR and Raman (Figures S3, S4) - should allow the identification of the different conformers of the same tautomer (e.g. (*Z*)-2,6-dipyridone vs. (*Z*)-2,6-dipyridone-c1).

(*Z*)-6-dipyridone	(*Z*)-2,6-dipyridone-c1
mode #1: 1665 cm^{-1} (1632 cm^{-1}); I_{IR} = 910 km/mol	mode #1: 1736 cm^{-1} (1701 cm^{-1}); I_{IR} = 460 km/mol
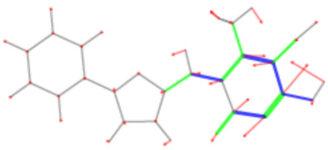	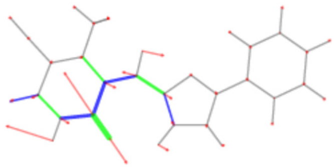
mode #2: 1717 cm^{-1} (1683 cm^{-1}); I_{IR} = 143 km/mol	mode #2: 1756 cm^{-1} (1721 cm^{-1}); I_{IR} = 636 km/mol
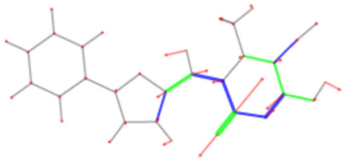	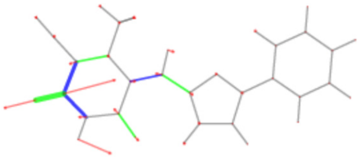

Scheme S2. Sketch of the DFT computed vibrational eigenvectors associated to C=O stretching modes of (*Z*)-6-dipyridone and (*Z*)-2,6-dipyridone-c1 tautomers of compound **8**: green/blue segments correspond to bond stretching/shrinking. Wavenumber (scaled values in parenthesis) and IR intensities are reported for each mode.
